# MLcps: machine learning cumulative performance score for classification problems

**DOI:** 10.1093/gigascience/giad108

**Published:** 2023-12-13

**Authors:** Akshay Akshay, Masoud Abedi, Navid Shekarchizadeh, Fiona C Burkhard, Mitali Katoch, Alex Bigger-Allen, Rosalyn M Adam, Katia Monastyrskaya, Ali Hashemi Gheinani

**Affiliations:** Functional Urology Research Group, Department for BioMedical Research DBMR, University of Bern, 3008 Bern, Switzerland; Graduate School for Cellular and Biomedical Sciences, University of Bern, 3012 Bern, Switzerland; Department of Medical Data Science, Leipzig University Medical Centre, 04107 Leipzig, Germany; Department of Medical Data Science, Leipzig University Medical Centre, 04107 Leipzig, Germany; Center for Scalable Data Analytics and Artificial Intelligence (ScaDS.AI) Dresden/Leipzig, 04105 Leipzig, Germany; Functional Urology Research Group, Department for BioMedical Research DBMR, University of Bern, 3008 Bern, Switzerland; Department of Urology, Inselspital University Hospital, 3010 Bern, Switzerland; Institute of Neuropathology, Universitätsklinikum Erlangen, Friedrich-Alexander-Universität Erlangen-Nürnberg (FAU), 91054 Erlangen, Germany; Biological & Biomedical Sciences Program, Division of Medical Sciences, Harvard Medical School, 02115 Boston, MA, USA; Urological Diseases Research Center, Boston Children's Hospital, 02115 Boston, MA, USA; Department of Surgery, Harvard Medical School, 02115 Boston, MA, USA; Broad Institute of MIT and Harvard, 02142 Cambridge, MA, USA; Urological Diseases Research Center, Boston Children's Hospital, 02115 Boston, MA, USA; Department of Surgery, Harvard Medical School, 02115 Boston, MA, USA; Broad Institute of MIT and Harvard, 02142 Cambridge, MA, USA; Functional Urology Research Group, Department for BioMedical Research DBMR, University of Bern, 3008 Bern, Switzerland; Department of Urology, Inselspital University Hospital, 3010 Bern, Switzerland; Functional Urology Research Group, Department for BioMedical Research DBMR, University of Bern, 3008 Bern, Switzerland; Department of Urology, Inselspital University Hospital, 3010 Bern, Switzerland; Urological Diseases Research Center, Boston Children's Hospital, 02115 Boston, MA, USA; Department of Surgery, Harvard Medical School, 02115 Boston, MA, USA; Broad Institute of MIT and Harvard, 02142 Cambridge, MA, USA

**Keywords:** machine learning, classification problems, model evaluation, unified evaluation score, Python package

## Abstract

**Background:**

Assessing the performance of machine learning (ML) models requires careful consideration of the evaluation metrics used. It is often necessary to utilize multiple metrics to gain a comprehensive understanding of a trained model’s performance, as each metric focuses on a specific aspect. However, comparing the scores of these individual metrics for each model to determine the best-performing model can be time-consuming and susceptible to subjective user preferences, potentially introducing bias.

**Results:**

We propose the Machine Learning Cumulative Performance Score (MLcps), a novel evaluation metric for classification problems. MLcps integrates several precomputed evaluation metrics into a unified score, enabling a comprehensive assessment of the trained model’s strengths and weaknesses. We tested MLcps on 4 publicly available datasets, and the results demonstrate that MLcps provides a holistic evaluation of the model’s robustness, ensuring a thorough understanding of its overall performance.

**Conclusions:**

By utilizing MLcps, researchers and practitioners no longer need to individually examine and compare multiple metrics to identify the best-performing models. Instead, they can rely on a single MLcps value to assess the overall performance of their ML models. This streamlined evaluation process saves valuable time and effort, enhancing the efficiency of model evaluation. MLcps is available as a Python package at https://pypi.org/project/MLcps/.

Key points

- Evaluating machine learning models involves considering multiple metrics. Comparing scores of individual metrics to determine the best model can be time-consuming and subjective, potentially introducing bias.
- The proposed Machine Learning Cumulative Performance Score (MLcps) is a novel evaluation metric for classification problems. It integrates multiple evaluation metrics into a unified score, providing a holistic understanding of model performance.
- MLcps outperforms standard metric-based rankings, offering a more reliable and consistent assessment of model performance.
- MLcps is available as a Python package, making it easily accessible for researchers to incorporate into their evaluation pipelines.

## Introduction

The evaluation of machine learning (ML) models is crucial in the ML workflow as it helps determine their effectiveness. However, it is essential to select the appropriate evaluation metric since the performance of a trained model is only as good as the metric used for evaluation [1–5]. Numerous metrics are available for assessing the performance of ML models, with each metric focusing on a specific aspect of the model’s performance [6, 7]. For example, the “recall” metric effectively measures a model’s ability to predict positive class instances but does not provide insights into the negative class instances. This poses a significant challenge because a model that performs well according to one metric may not exhibit the same level of performance when evaluated using another metric [8–14]. Hence, relying solely on a single performance metric is inadequate in practical scenarios.

Furthermore, the characteristics and composition of the available dataset can influence the behavior and outcomes of various metrics. For instance, when dealing with imbalanced datasets, accuracy becomes an inadequate metric, and relying solely on accuracy can lead to misleading interpretations [15]. Therefore, it is crucial to calculate multiple performance metrics for each model to evaluate its performance comprehensively [7]. By considering various evaluation metrics, we can gain a holistic view of a model’s performance and make informed decisions about the best-performing model for a given task.

When calculating multiple metrics for a model, there is often an assumption that the best model will consistently achieve the highest scores across all metrics. However, this assumption is rarely true in practical scenarios, necessitating the comparison of the individual metrics of different models to identify the best-performing model. However, comparing metric scores for many models can be labor-intensive and susceptible to user preference bias [16]. As a result, the complexity of finding the best model increases exponentially when considering the comparison of different metrics.

Apart from these limitations, some methods prevent users from evaluating model performance with multiple metrics simultaneously. For example, in the field of biology, the wrapper-based feature selection method is commonly used to identify important features from a large set of original attributes. This method trains a model with different feature subsets and selects the subset that shows the best performance compared to the other subsets. Unfortunately, these methods are limited to evaluating model performance using only one metric at a time. This constraint can potentially lead to overfitting to a specific metric, resulting in the selection of suboptimal feature subsets that lack generalizability.

In the realm of information retrieval (IR), Chakrabarti et al. [17] previously introduced novel algorithms designed to merge multiple ranking criteria into a unified approach, ultimately enhancing the optimization of search results. Building upon this research, Geng and Cheng [18] further investigated learning to rank, considering multiple evaluation metrics, and proposed the combination of multiple metrics to optimize IR metrics.

Here, we introduce a novel evaluation metric called the Machine Learning Cumulative Performance Score (MLcps) to address the challenges associated with model evaluation in the field of machine learning. MLcps is a unified score that follows a similar methodology compared to the previously mentioned study related to IR. MLcps combines precomputed performance metrics into a single score while preserving their distinct characteristics. By leveraging multiple metrics, MLcps provides a more comprehensive evaluation of machine learning model performance. To enhance the accessibility of MLcps, we have implemented it as a Python package, enabling direct comparisons of trained ML models to assess their performance.

## Results and Discussion

In this section, the results of the current study are showcased, with a specific focus on evaluating MLcps as a robust measure for assessing ML model performance. The primary objective of this analysis is to shed light on the effectiveness of MLcps in ranking models based on their consistency and excellence across multiple performance metrics. Furthermore, we explore the reliability of MLcps in selecting models that not only excel on training data but also demonstrate the ability to generalize well to unseen datasets.

Additionally, we emphasize the importance of employing a diverse set of performance metrics when evaluating machine learning models. By doing so, we aim to provide a comprehensive understanding of model performance beyond traditional measures and showcase the significance of considering various aspects of model behavior in real-world applications.

### Evaluating MLcps robustness

Each performance metric represents a specific aspect of model performance, and for a model to be considered robust and superior, it should consistently excel across all these metrics. This consistency can be reflected by having the lowest standard deviation (SD) across performance metrics. Therefore, our analysis revolves around understanding the relationship between MLcps and SD. This evaluation helps determine the reliability of MLcps as a performance measure.

**Figure 1:**
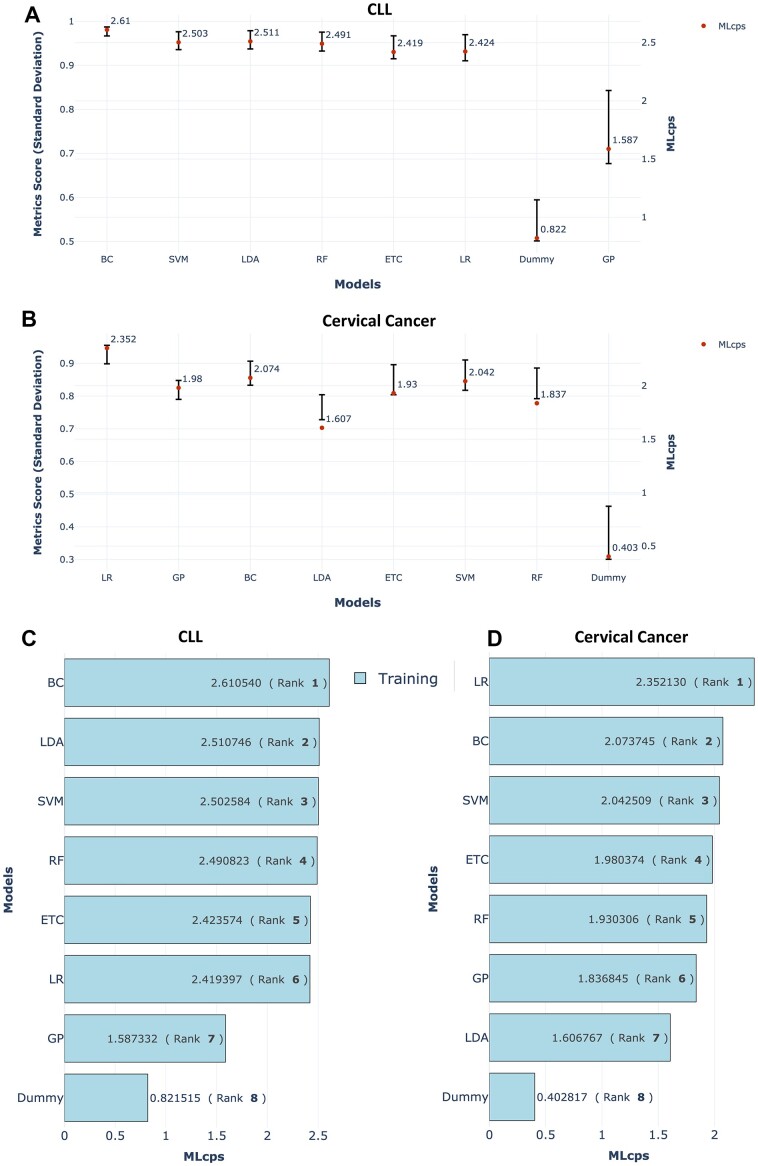
SD of performance metrics and MLcps comparison for CLL and cervical cancer datasets. (A, B) The SD of performance metric scores for ML algorithms trained on the CLL and cervical cancer datasets, respectively. The bars in the plot represent the SD of performance metric scores and are displayed on the left y-axis. The bars are arranged from left to right, with smaller SD values on the left and larger SD values on the right. A red dot on the plot represents the MLcps, which is displayed on the right y-axis. (C, D) MLcps for training data from the CLL and cervical cancer datasets, respectively. The numerical MLcps values are indicated within each bar. Rankings, enclosed in brackets, reflect model performance based on MLcps.

**Figure 2:**
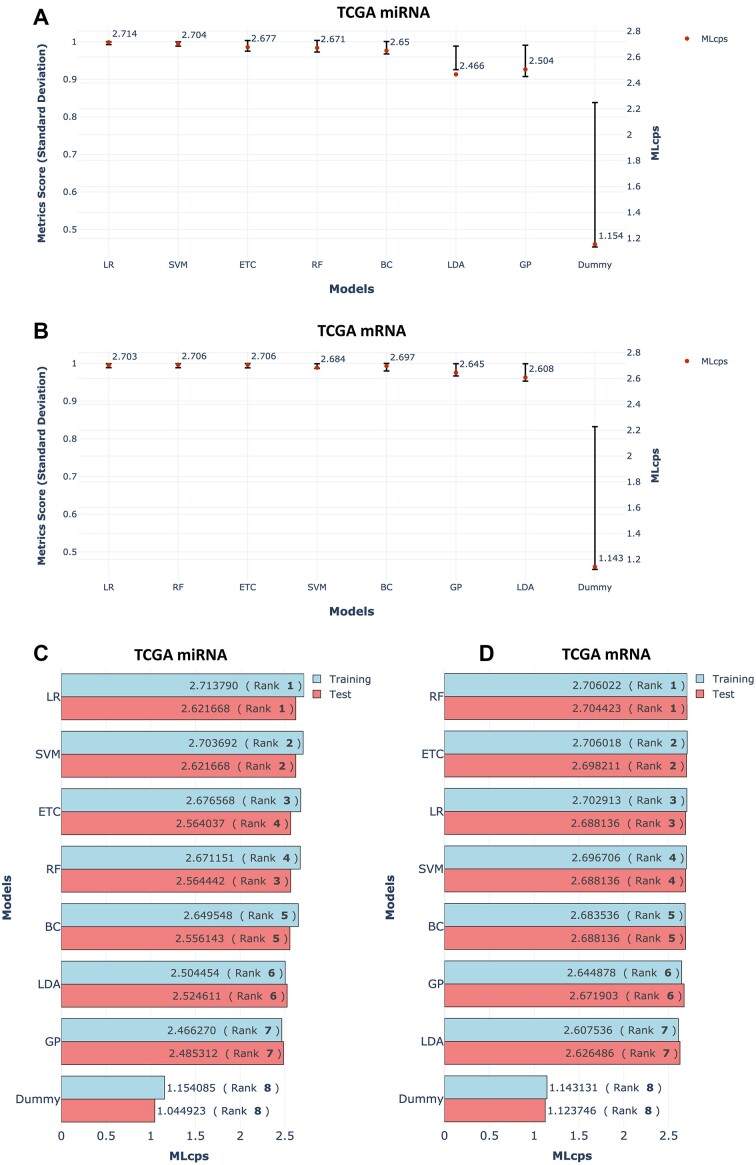
SD of performance metrics and MLcps comparison for TCGA mRNA and miRNA datasets. (A, B) The SD of performance metric scores for ML algorithms trained on the mRNA and miRNA datasets, respectively. The bars in the plot represent the SD of performance metric scores and are displayed on the left y-axis. The bars are arranged from left to right, with smaller SD values on the left and larger SD values on the right. A red dot on the plot represents the MLcps, which is displayed on the right y-axis. (C, D) A comparison of MLcps for training and test data from the mRNA and miRNA datasets, respectively. The numerical MLcps values are indicated within each bar. Rankings, enclosed in brackets, reflect model performance based on MLcps, whether computed from the training or test data.

To assess MLcps’ robustness as a model performance measure, we analyzed multiple models across 5 distinct datasets (Table 1). Our findings consistently revealed a strong correlation between the highest MLcps score and the lowest SD in performance metric scores (Fig. 1A, B and Fig. 2A, B). This correlation indicates that MLcps reliably identifies the best-performing model when it consistently excels across all metrics, validating its reliability as a performance measure.

**Table 1:** Example datasets used in this study

Dataset	Data type	Number of samples	Number of features	Target class ratio
CLL	mRNA	136	5,000	Male (*n* = 82)/Female (*n* = 54)
Cervical cancer	miRNA	58	714	Normal (*n* = 29)/Tumor (*n* = 29)
TCGA-BRCA	miRNA	1,207	1,404	Normal (*n* = 104)/Tumor (*n* = 1,104)
TCGA-BRCA	mRNA	1,219	5,520	Normal (*n* = 113)/Tumor (*n* = 1,106)
Body signal	Body signal data (hemoglobin, triglyceride)	100,000	21	**Consume Alcohol** Yes (*n* = 50,173)/No (*n* = 49,827)

However, there are important exceptions that require attention. For instance, in the chronic lymphocytic leukemia (CLL) dataset, the GP model outperforms the dummy model in terms of MLcps score, even though the dummy model has a lower SD (Fig. 1A). Similarly, in the cervical cancer dataset, the MLcps scores of the extra trees classifier (ETC), support vector machine (SVM), and random forest (RF) classifier models surpass that of the linear discriminant analysis (LDA) model, despite the LDA model having a lower SD (Fig. 1B). Similar exceptions were observed in the body signals dataset as well (Supplementary Fig. S4A).

These exceptions can be attributed to the fact that while these models exhibit lower SD compared to others, they also perform poorly for each individual metric. Consequently, their low MLcps scores accurately reflect their subpar performance across all metrics. This observation acknowledges that a model with poor performance metrics may still have a smaller SD when compared to other models. These exceptions underscore that MLcps takes into account not only the SD but also the overall magnitude of performance metric scores, thereby providing a comprehensive evaluation of ML models’ performance.

### Consistency in model performance across training and test datasets

To evaluate the reliability of MLcps in selecting the best-performing models, we examined the consistency of model performance between the training and test datasets. Among the 5 datasets, the The Cancer Genome Atlas (TCGA) breast invasive carcinoma (BRCA) and body signals datasets offered a larger sample size, allowing us to create an independent test set comprising 30% of the data. When analyzing these 3 datasets, we found that the model identified as the best performer based on MLcps also demonstrated the best performance on the independent test set (Fig. 2C, D).

Furthermore, it is noteworthy that if we solely relied on the SD to rank the models, the Logistic Regression (LR) model would have been chosen as the best performer on the training dataset of TCGA-BRCA mRNA (Fig. 2B). However, when evaluating its performance on the test dataset, LR did not even rank among the top 2 (Fig. 2D). Similarly, in the body signals dataset, the bagging classifier model would have been considered the best performer based on the SD criteria (Supplementary Fig. S4A). However, it is important to note that on the test dataset, this model ranked fourth in terms of performance (Supplementary Fig. S4B).

In contrast, when sorting the model performance based on MLcps, the ranking remained consistent across both training and test datasets, providing a more robust measure of model performance (Supplementary Fig. S4B). These findings indicate that MLcps effectively identifies models that not only perform well on the training data but also generalize well to unseen data, highlighting its comprehensive ability to assess model performance across different datasets.

### Importance of utilizing multiple performance metrics

To emphasize the significance of using multiple performance metrics in evaluating ML model performance, we employed a visual representation of the metric scores using a 2-dimensional polar coordinate system for each ML algorithm trained on different datasets. Our results demonstrated that both precision and average precision metrics consistently yielded high scores (>90%) for all the trained models in the TCGA miRNA (Supplementary Fig. S1B, C) and mRNA datasets (Supplementary Fig. S2B, C). However, relying solely on these metrics would have resulted in mistakenly selecting the dummy model as the best-performing one. This highlights the crucial importance of incorporating multiple performance metrics to obtain a more accurate assessment of ML model performance. Importantly, this phenomenon was not observed in the CLL and cervical cancer datasets (Supplementary Figs. S1A, S2A), indicating that the interpretation of performance metrics is dataset dependent. By considering a diverse range of metrics, researchers and practitioners can make more informed decisions regarding the usefulness and reliability of ML models.

## Materials and Methods

### MLcps methodology

**Figure 3:**
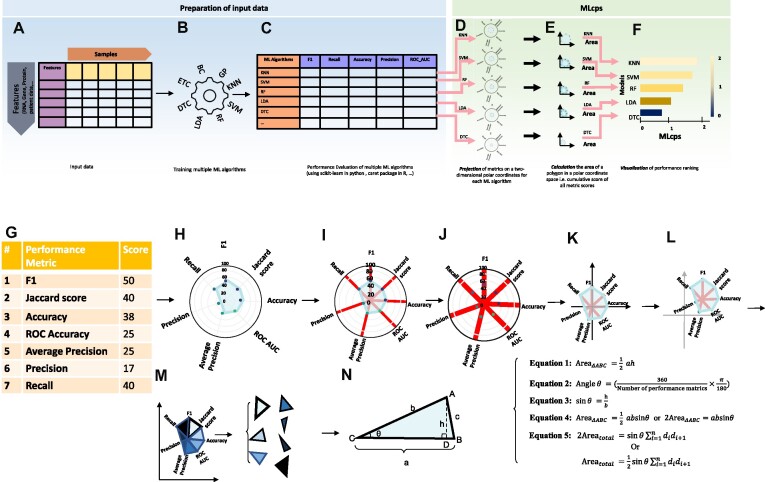
Schematic overview of the complete analysis process for MLcps Python package. Before using the MLcps Python package, one needs to prepare the raw data (A). This input table can be RNA sequencing, proteomics, patients’ profile, molecular data, and so on (normally these data are in txt or csv format). Next step is to perform multiple ML algorithms (B). Performing this step can be done by any package or programming language of choice. The next step is to evaluate the performance of the ML algorithms. We recommend the use of multiple metrics such as F1, recall, and so on (C). The performance metric scores then need to be arranged in a tabular format, as depicted in (C). This table will be used as an input for the MLcps package. From here on, the MLcps will process the data. MLcps involves 3 steps: projection, calculation, and visualization (PCV). To calculate the cumulative score of each ML algorithm in the input data, MLcps first projects the performance metric onto the 2-dimensional polar coordinates system (D). Next, the projected polygon’s area is calculated (E). Finally, the user can visualize this MLcps to rank the performance of given ML algorithms (F). The lower panel (G–N) visualizes the procedure to calculate the surface area as a cumulative score in detail. The names of the algorithms are just mentioned as an example and other algorithms can be used too. BC: bagging classifier; DTC: decision tree classifier; ETC: extra trees classifier; GP: Gaussian process classifier; KNN: k-nearest neighbors; LDA: linear discriminant analysis; RF: random forest classifier; SVM: support vector machine.

The MLcps algorithm requires an input table consisting of columns that hold various performance metrics, such as F1, accuracy, and recall. The rows in the table represent different machine learning methods, such as k-nearest neighbors (KNN) and SVM. Typically, this table is generated as the output of a standard machine learning pipeline (Fig. 3A–C). In principle, MLcps can be calculated for any evaluation metric. However, it is highly recommended that all of them are on the same scale; for example, if accuracy ranges between 0 and 1, then the F1 metric should also be in the same range, not in percentages.

To calculate MLcps, the first step involves plotting the precalculated performance metrics on a 2-dimensional polar coordinate system (Fig. 3D). In this polar coordinate system, each metric is represented as a ray, and the length of the ray corresponds to the metric value. This representation allows the polar plane to be divided into multiple triangles, with the number of triangles being equal to the available evaluation metrics. The combined area of these individual triangles represents the total area of the polar plane and serves as the MLcps (Fig. 3E).

Finally, the MLcps can be visually represented using a bar chart, as shown in Fig. 3F. It provides a clear and visually informative depiction of the relative performance of different machine learning methods. By examining the bar chart, one can easily identify the performance differences between various ML methods.

### Area calculation of a 2-dimensional polar plane

The projection of multiple evaluation metrics onto a 2-dimensional polar coordinate system divides the polar plane into several triangles. Therefore, the total sum of the areas of these triangles is equal to the total area of the polar plane generated by the multiple performance scores. In order to calculate the area of each individual triangle, as described in Equation (1), we need to multiply half the length of base by the height drawn to that side (Fig. 3G–N).


(1) \begin{eqnarray*}
{\rm Area}_{\Delta ABC} = \frac{1}{2} \, ah
\end{eqnarray*}

where

*a* = represents the side (base), and
*h* = represents the height drawn to that side.

However, to apply this formula, we require the value for the height (*h*) variable, which cannot be controlled in a polar plane. Nonetheless, we do have control over the angles ($\theta $) of all the triangles, which can be calculated by dividing 360 degrees by the number of performance metrics used, as described in Equation (2).


(2) \begin{eqnarray*}
{\mathrm{Angle}}\ \theta &=& \frac{{360}}{{{\mathrm{Number\ of\ performance\ metrics}}}} \times \frac{\pi }{{180}}\\ &=& \frac{{2\pi }}{{{\mathrm{Number\ of\ performance\ metrics}}}}
\end{eqnarray*}

Now, by employing trigonometry, as outlined in Equation (3), we can calculate the height (*h*) based on the known angles ($\theta $). Therefore, the height of the triangle can be expressed as $h = b\ sin\theta $.


(3) \begin{eqnarray*}
\sin \theta \ = \frac{{\mathrm{h}}}{b}
\end{eqnarray*}

By substituting the new expression for the height (*h*) variable into the general formula for the area of a triangle, we obtain a new formula, as shown in Equation (4), where values for all the required variables are available.


(4) \begin{eqnarray*}
{\mathrm{Are}}{{\mathrm{a}}}_{\Delta ABC} = \frac{1}{2}ab\,\,\sin \theta \,\,{\mathrm{\ or}}\,\,\ 2{\mathrm{Are}}{{\mathrm{a}}}_{\Delta ABC} = ab\sin \theta
\end{eqnarray*}

In Equation (4), the parameters *a* and *b* represent any 2 sides of a triangle, while $\theta $ denotes the included angle. It is important to note that in this context, the values *a* and *b* correspond to the actual measurements for each performance metric.

Finally, by utilizing Equation (5), derived from Equation (4), the total area of the polar plane can be determined by summing the areas of all triangles formed within the polar coordinate system.


(5) \begin{eqnarray*}
2{\mathrm{Are}}{{\mathrm{a}}}_{{\mathrm{total}}} = \sin \theta \mathop \sum \limits_{i = 1}^n {d}_i{d}_{i + 1}\ \to \ {\mathrm{Are}}{{\mathrm{a}}}_{{\mathrm{total}}} = \frac{1}{2}\sin \theta \mathop \sum \limits_{i = 1}^n {d}_i{d}_{i + 1}
\end{eqnarray*}

where

${d}_i$ = length of the *i*th ray (the value of *i*th metric score) (Fig. 3L), and
*n* = number of triangles point of collapse (Fig. 3M).

### Weighted MLcps

In specific situations, certain metrics hold more significance than others. For instance, when dealing with an imbalanced dataset, achieving a high F1 score may be prioritized over higher accuracy [19, 20]. In such cases, users have the option to assign weight variables to the metrics of interest during the calculation of MLcps. A weight variable assigns a value (referred to as the weight) to each precomputed metric, and the respective metric scores are adjusted using these weights in the following manner:


(6) \begin{eqnarray*}
{{\mathrm{S}}}_{{\mathrm{weightedmetric}}}{\mathrm{\ }} = {\mathrm{\ }}{{\mathrm{S}}}_{{\mathrm{metric}}}{\mathrm{\ }} \times {\mathrm{\ }}{{\mathrm{W}}}_{{\mathrm{metric}}}
\end{eqnarray*}

where

${{\mathrm{S}}}_{{\mathrm{weightedmetric}}}$ = weighted metric score,

${{\mathrm{S}}}_{{\mathrm{metric}}}$ = raw metric score, and

${{\mathrm{W}}}_{{\mathrm{metric}}}$ = weight.

It is essential to note that the assigned weight for a metric must always be greater than or equal to zero. A weight of zero indicates that the user intends to exclude that metric from the MLcps calculation. Metrics with higher weights have a more significant contribution to the MLcps compared to metrics with lower weights. In the case where no weights are assigned (unweighted MLcps), it is equivalent to conducting a weighted analysis where all weights are set to 1.

### Datasets

In this study, 4 distinct datasets were employed to evaluate MLcps (Table 1). The initial dataset comprises mRNA data (*n* = 136) derived from a CLL study, which examined transcriptome profiles in individuals affected by blood cancer [21]. Our objective was to develop a model capable of distinguishing between male and female patients using their transcriptomic profiles. To achieve this, we focused on the top 5,000 most variably expressed mRNAs, excluding genes from the Y chromosome.

The second set of data was obtained from a study on cervical cancer, where the expression levels of 714 miRNAs were measured in human samples (*n* = 58) [22]. The third and fourth datasets were collected from TCGA and involved mRNA (*n* = 1,219) and miRNA (*n* = 1,207) sequencing of BRCA. The TCGAbiolinks package in R was used to retrieve these datasets [23]. For the BRCA mRNA dataset, we focused on genes that showed differential expression according to edgeR analysis (False discovery rate (FDR) ⇐ 0.001 and Fold Change log(FC) > ±2) [24]. Our objective was to develop a model capable of distinguishing between normal and tumor samples for both the cervical cancer and TCGA-BRCA datasets.

The fifth dataset in our study comprises body signal data collected from 100,000 individuals through the National Health Insurance Service in Korea [25]. This dataset includes 21 essential biological signals related to health, such as measurements of systolic blood pressure and total cholesterol levels. Our main goal with this dataset was to determine whether individuals consume alcohol based on the available biological signal information.

Among these datasets, 2 were relatively small (CLL and the cervical cancer study), while the other 2 (TCGA datasets) were imbalanced (Table 1). We utilized an in-house ML pipeline (Supplementary Fig. S5) to train and evaluate 8 different models (Supplementary Table S1) to identify the best-performing model for CLL, cervical cancer, and the TCGA datasets. For the biological signal dataset, we utilized the “customML” feature from the Machine Learning Made Easy [26] tool to train and evaluate 6 different models and identify the best-performing one for classifying alcohol consumers and nonconsumers.

### Implementation

MLcps is developed using Python [27] and R [28] programming languages. Pandas [29, 30] is used to store and process the data. Plotly [31] is used to generate the figures. The radarchart [32] package in R was used for surface area calculation of the polar plane. The R packages tibble [33] and dplyr [34] were utilized for data wrangling in the computation of MLcps during the analysis.

## Conclusions

Our article introduces MLcps, a novel evaluation metric implemented as a Python package. MLcps is a robust evaluation metric designed specifically for classification problems. Its ability to integrate multiple evaluation metrics into a single score makes it an efficient and reliable approach for evaluating model performance and selecting the most successful model. This is especially valuable when multiple evaluation metrics are necessary to fully comprehend a model’s strengths and weaknesses.

However, it is essential to understand that the reliability of MLcps depends on the quality of the metrics used in its calculation. Therefore, it is of utmost importance to employ appropriate evaluation metrics, which depend on various factors such as the specific domain, stakeholder preferences, and data characteristics. Similarly, assigning weights to evaluation metrics in machine learning offers a valuable technique for prioritizing specific aspects of model performance, but it comes with potential drawbacks and complexities. For example, heavily weighting one metric can overshadow the overall evaluation, possibly resulting in suboptimal models. Additionally, the assignment of metric weights often depends on subjective judgments regarding their relative significance. Various stakeholders may hold differing perspectives on how much weight to allocate to each metric, potentially leading to evaluation bias.

While the allocation of weights to evaluation metrics can enhance the customization of the evaluation process for specific objectives, it must be executed judiciously, considering the possible downsides and challenges associated with this approach. Striking a balance between highlighting key metrics and maintaining a comprehensive view of model performance is paramount. Therefore, we strongly discourage relying on MLcps without considering the context in which it is applied.

## Availability of Supporting Source Code and Requirements

Project name: Machine Learning Cumulative Performance Score (MLcps)

Project homepage: https://github.com/FunctionalUrology/MLcps

Operating system(s): Platform independent

Programming language: Python ≥3.8 and R ≥4.0

Other requirements: radarchart, tibble, and dplyr R packages

License: GNU GPL

BioTool ID: mlcps

RRID: SCR_024716

## Supplementary Material

giad108_GIGA-D-23-00187_Original_Submission

giad108_GIGA-D-23-00187_Revision_1

giad108_GIGA-D-23-00187_Revision_2

giad108_Response_to_Reviewer_Comments_Original_Submission

giad108_Response_to_Reviewer_Comments_Revision_1

giad108_Reviewer_1_Report_Original_Submission Ryan J. Urbanowicz -- 7/24/2023 Reviewed

giad108_Reviewer_1_Report_Revision_1 Ryan J. Urbanowicz -- 10/9/2023 Reviewed

giad108_Reviewer_2_Report_Original_Submission Kamil Dimililer -- 9/4/2023 Reviewed

giad108_Supplemental_Files

## Data Availability

An archival copy of the code and supporting data is available via the *GigaScience* repository, GigaDB [35]. DOME-ML (Data, Optimisation, Model, and Evaluation in Machine Learning) annotations, supporting the current study, are available via the supporting data in GigaDB.

## Additional Files

**Supplementary Fig. S1**. Projection of metric scores onto a two-dimensional (2D) polar coordinate system for each ML algorithm trained on different example datasets. The plots represent A) CLL dataset, B) TCGA miRNA Training dataset, C) TCGA miRNA test dataset.

**Supplementary Fig. S2**. Projection of metric scores onto a two-dimensional (2D) polar coordinate system for each ML algorithm trained on different example datasets. The plots represent A) Cervical cancer dataset, B) TCGA mRNA Training dataset, C) TCGA mRNA test dataset.

**Supplementary Fig. S3**. Projection of metric scores onto a two-dimensional (2D) polar coordinate system for each ML algorithm trained on Body SIgnal dataset. The plots represent model performance on A) Training Data, and B) Test Data.

**Supplementary Fig. S4**. Body Signal Dataset Results. A) Standard deviation (SD) in ML Algorithm Performance. This plot displays the SD of performance metric scores for ML algorithms trained on body signal datasets. Bars represent the SD of performance metric scores, as shown on the left y-axis. The bars are arranged from left to right, with smaller SD on the left and larger SD on the right. A red dot on the plot represents MLcps, displayed on the right y-axis. B) MLcps Comparison for Training and Test Data. This bar charts represents MLcps obtained from the training and test data of the body signal dataset. Each bar is color-coded, and the numerical MLcps values are shown within each bar. Rankings, enclosed in brackets, reflect model performance based on MLcps, whether computed from the training or test data within the body signal dataset.

**Supplementary Fig. S5**. Flowchart describing ML Pipeline:  Firstly, the dataset is divided into k (3) equal-sized bins in a stratified manner, with k-1 bins used for training and the remaining bin for testing. Next, the pipeline applies the univariate feature selection method to select relevant features from the dataset. Data resampling is then performed using the SMOTETomek method, which combines synthetic data generation for the minority class and removal of majority class samples identified as Tomek links. Eight ML algorithms are trained on the pre-processed dataset. The model performance is evaluated using k-fold cross-validation (CV) and nested CV (k=3), calculating seven different performance metrics. This process is repeated for each unique bin within the k-fold CV method, ensuring comprehensive evaluation across subsets of the dataset. The entire pipeline is repeated ten times, and the average performance is considered the final model performance. Finally, the pipeline provides a list of selected features, derived from the intersection of features chosen by the top 10 best-performing models based on the F1 score.

**Supplementary Table S1**. ML algorithms used in this study.

## Abbreviations

BRCA: breast invasive carcinoma; CLL: chronic lymphocytic leukemia; ETC: extra trees classifier; IR: information retrieval; KNN: k-nearest neighbors; LDA: linear discriminant analysis; ML: machine learning; MLcps: Machine Learning Cumulative Performance Score; RF: random forest classifier; SD: standard deviation; SVM: support vector machine; TCGA: The Cancer Genome Atlas.
