# Supplementary material for: MLcps: machine learning cumulative performance score for classification problems
Source: Gigascience. 2023 Dec 13;12:giad108. doi: 10.1093/gigascience/giad108 (PMC10716825; doi:10.1093/gigascience/giad108)
Supplement: giad108_GIGA-D-23-00187_Original_Submission [file giad108_giga-d-23-00187_original_submission.pdf]

GigaScience

MLcps: Machine Learning Cumulative Performance Score for Classification Problems

--Manuscript Draft--

**Manuscript Number:**	GIGA-D-23-00187
**Full Title:**	MLcps: Machine Learning Cumulative Performance Score for Classification Problems
**Article Type:**	Technical Note
**Funding Information:**	Schweizerischer Nationalfonds zur Förderung der Wissenschaftlichen Forschung (310030_175773) — Prof Fiona C. Burkhard<br>Schweizerischer Nationalfonds zur Förderung der Wissenschaftlichen Forschung (212298) — Prof Fiona C. Burkhard<br>Wings for Life (WFL-AT-06/19) — Prof Katia Monastyrskaya<br>Else Kröner-Fresenius-Stiftung (EKFS 2021_EKeA.33) — Not applicable<br>Sächsisches Staatsministerium für Wissenschaft und Kunst (ScaDS.AI) — Not applicable
**Abstract:**	Background<br>Assessing the performance of machine learning (ML) models requires careful consideration of the evaluation metrics used. It is often necessary to utilize multiple metrics to gain a comprehensive understanding of a trained model's performance, as each metric focuses on a specific aspect. However, comparing the scores of these individual metrics for each model to determine the best-performing model can be time-consuming and susceptible to subjective user preferences, potentially introducing bias.<br>Results<br>We propose Machine Learning Cumulative Performance Score (MLcps), a novel evaluation metric for classification problems. MLcps integrates several pre-computed evaluation metrics into a unified score, enabling a comprehensive assessment of the trained model's strengths and weaknesses. We tested MLcps on four publicly available datasets, and the results demonstrate that MLcps provides a holistic evaluation of the model's robustness, ensuring a thorough understanding of its overall performance.<br>Conclusion<br>By utilizing MLcps, researchers and practitioners no longer need to individually examine and compare multiple metrics to identify the best-performing models. Instead, they can rely on a single MLcps value to assess the overall performance of their ML models. This streamlined evaluation process saves valuable time and effort, enhancing the efficiency of model evaluation. MLcps is available as a python package at https://pypi.org/project/MLcps/ and examples of its use can be found at https://mybinder.org/v2/gh/FunctionalUrology/MLcps.git/main.
**Corresponding Author:**	Ali Hashemi Gheinani<br>Boston Children's Hospital<br>SWITZERLAND
**Corresponding Author Secondary Information:**	
**Corresponding Author's Institution:**	Boston Children's Hospital
**Corresponding Author's Secondary Institution:**	
**First Author:**	Akshay Akshay
**First Author Secondary Information:**	
**Order of Authors:**	Akshay Akshay<br>Masoud Abedi

Powered by Editorial Manager® and ProduXion Manager® from Aries Systems Corporation

	Navid Shekarchizadeh
	Fiona C. Burkhard
	Mitali Katoch
	Alex Bigger-Allen
	Rosalyn M. Adam
	Katia Monastyrskaya
	Ali Hashemi Gheinani
**Order of Authors Secondary Information:**	
**Additional Information:**	
**Question**	**Response**
Are you submitting this manuscript to a special series or article collection?	No
**Experimental design and statistics**<br><br>Full details of the experimental design and statistical methods used should be given in the Methods section, as detailed in our Minimum Standards Reporting Checklist. Information essential to interpreting the data presented should be made available in the figure legends.<br><br>Have you included all the information requested in your manuscript?	Yes
**Resources**<br><br>A description of all resources used, including antibodies, cell lines, animals and software tools, with enough information to allow them to be uniquely identified, should be included in the Methods section. Authors are strongly encouraged to cite Research Resource Identifiers (RRIDs) for antibodies, model organisms and tools, where possible.<br><br>Have you included the information requested as detailed in our Minimum Standards Reporting Checklist?	Yes
**Availability of data and materials**	Yes

Powered by Editorial Manager® and ProduXion Manager® from Aries Systems Corporation

All datasets and code on which the conclusions of the paper rely must be either included in your submission or deposited in publicly available repositories (where available and ethically appropriate), referencing such data using a unique identifier in the references and in the "Availability of Data and Materials" section of your manuscript.<br><br>Have you have met the above requirement as detailed in our Minimum Standards Reporting Checklist?	

Powered by Editorial Manager® and ProduXion Manager® from Aries Systems Corporation

Manuscript

Click here to access/download;Manuscript;main_KM230705.docx

MLcps: Machine Learning Cumulative Performance Score for Classification Problems

Akshay Akshay [1,2], Masoud Abedi[3], Navid Shekarchizadeh[3,4], Fiona C. Burkhard[1,5], Mitali Katoch[6], Alex Bigger-Allen [7,8,9,10], Rosalyn M. Adam [8,9,10], Katia Monastyrskaya [1, 5] and Ali Hashemi Gheinani [1,5,8,9,10*]

[1] Functional Urology Research Group, Department for BioMedical Research DBMR, University of Bern, Switzerland

[2] Graduate School for Cellular and Biomedical Sciences, University of Bern, Switzerland

[3]Department of Medical Data Science, Leipzig University Medical Centre, 04107 Leipzig, Germany

[4]Center for Scalable Data Analytics and Artificial Intelligence (ScaDS.AI) Dresden/Leipzig, 04105 Leipzig, Germany

[5] Department of Urology, Inselspital University Hospital, 3010 Bern, Switzerland

[6] Institute of Neuropathology, Universitätsklinikum Erlangen, Friedrich-Alexander-Universität Erlangen-Nürnberg (FAU), Erlangen, Germany

[7] Biological & Biomedical Sciences Program, Division of Medical Sciences, Harvard Medical School, Boston, MA.

[8] Urological Diseases Research Center, Boston Children’s Hospital, MA, USA

[9] Harvard Medical School, Boston, Department of Surgery MA, USA

[10] Broad Institute of MIT and Harvard, Cambridge, MA, USA

* Corresponding author:

Ali Hashemi Gheinani, Urological Diseases Research Center, Boston Children’s Hospital, Harvard Medical School and Broad Institute of MIT and Harvard, Cambridge, MA, USA

e-mail: Ali.HashemiGheinani@childrens.harvard.edu

Keywords

- Machine Learning
- Classification Problems
- Model Evaluation
- Unified Evaluation Score
- Python Package

Key Points

- Evaluating machine learning models involves considering multiple metrics. Comparing scores of individual metrics to determine the best model can be time-consuming and subjective, potentially introducing bias.

- The proposed Machine Learning Cumulative Performance Score (MLcps) is a novel evaluation metric for classification problems. It integrates multiple evaluation metrics into a unified score, providing a holistic understanding of model performance.

- MLcps outperforms standard metric-based rankings, offering a more reliable and consistent assessment of model performance.

- MLcps is available as a Python package, making it easily accessible for researchers to incorporate into their evaluation pipelines.

Abstract

Background

Assessing the performance of machine learning (ML) models requires careful consideration of the evaluation metrics used. It is often necessary to utilize multiple metrics to gain a comprehensive understanding of a trained model's performance, as each metric focuses on a specific aspect. However, comparing the scores of these individual metrics for each model to determine the best-performing model can be time-consuming and susceptible to subjective user preferences, potentially introducing bias.

Results

We propose Machine Learning Cumulative Performance Score (MLcps), a novel evaluation metric for classification problems. MLcps integrates several pre-computed evaluation metrics into a unified score, enabling a comprehensive assessment of the trained model's strengths and weaknesses. We tested MLcps on four publicly available datasets, and the results demonstrate that MLcps provides a holistic evaluation of the model's robustness, ensuring a thorough understanding of its overall performance.

Conclusion

By utilizing MLcps, researchers and practitioners no longer need to individually examine and compare multiple metrics to identify the best-performing models. Instead, they can rely on a single MLcps value to assess the overall performance of their ML models. This streamlined evaluation process saves valuable time and effort, enhancing the efficiency of model evaluation. MLcps is available as a python package at https://pypi.org/project/MLcps/ and examples of its use can be found at https://mybinder.org/v2/gh/FunctionalUrology/MLcps.git/main.

Introduction

The evaluation of machine learning (ML) models is crucial in the ML workflow as it helps determine their effectiveness. However, it is essential to select the appropriate evaluation metric since the performance of a trained model is only as good as the metric used for evaluation [1]. Numerous metrics are available for assessing the performance of ML models, with each metric focusing on a specific aspect of the model's performance. For example, the "recall" metric effectively measures a model's ability to predict positive class instances but does not provide insights into the negative class instances. This poses a significant challenge because a model that performs well according to one metric may not exhibit the same level of performance when evaluated using another metric [2-4]. Hence, relying solely on a single performance metric is inadequate in practical scenarios.

Furthermore, the characteristics and composition of the available dataset can influence the behaviour and outcomes of various metrics. For instance, when dealing with imbalanced datasets, accuracy becomes an inadequate metric, and relying solely on accuracy can lead to misleading interpretations [5]. Therefore, it is crucial to calculate multiple performance metrics for each model to evaluate its performance comprehensively. By considering various evaluation metrics, we can gain a holistic view of a model's performance and make informed decisions about the best-performing model for a given task.

When calculating multiple metrics for a model, there is often an assumption that the best model will consistently achieve the highest scores across all metrics. However, this assumption is rarely true in practical scenarios, necessitating the comparison of the individual metrics of different models to identify the best-performing model. However, comparing metric scores for many models can be labour-intensive and susceptible to user preference bias [6]. As a result, the complexity of finding the best model increases exponentially when considering the comparison of different metrics.

Apart from these limitations, some methods prevent users from evaluating model performance with multiple metrics simultaneously. For example, in the field of biology, the wrapper-based feature selection method is commonly used to identify important features from a large set of original attributes. This method trains a model with different feature subsets and selects the subset that shows the best performance compared to the other subsets. Unfortunately, these methods are limited to evaluating model performance using only one metric at a time. This constraint can potentially lead to overfitting to a specific metric, resulting in the selection of suboptimal feature subsets that lack generalizability.

To address these challenges, we propose a new metric called the Machine Learning Cumulative Performance Score (MLcps). MLcps is a unified score that combines several precomputed performance metrics while preserving their distinct characteristics. To make it easily accessible, we have implemented MLcps as a Python package, enabling direct comparison of trained ML models to assess their performance.

Results and Discussion

Evaluation of MLcps Robustness

Each performance metric represents a specific aspect of model performance. For a model to be considered robust or the best performing one, it must consistently achieve high and similar scores across all computed metrics. Therefore, the best performing model should have the lowest standard deviation (SD) across the performance metrics. Consequently, it is crucial to examine the relationship between MLcps and SD to evaluate the reliability of MLcps as a measure of model performance.

During the evaluation of MLcps, we assessed the performance of multiple models across four distinct datasets (Table 1). Our findings consistently indicate that the model with the highest MLcps score typically exhibits the lowest SD in performance metric scores (Figures 1A-D, 2A-F). This suggests that MLcps tends to rank a model as the best performing one when it consistently performs well across all metrics.

However, there are a few noteworthy exceptions that should be highlighted. In the Chronic Lymphocytic Leukemia (CLL) dataset, the GP model (refer to Table S1 for abbreviation expansions) outperforms the Dummy model in terms of MLcps score, despite the Dummy model having a lower SD (Figures 1A & 2A). Similarly, in the cervical cancer dataset, the MLcps scores of the ETC, SVM, and RF models are higher than that of the LDA model, even though the LDA model has a lower SD (Figures 1B & 2B). These exceptions can be explained by the fact that while these models may have a lower SD compared to other models, they also have poor performance scores for each metric. Consequently, their low MLcps scores reflect their poor performance across all the metrics. This acknowledges the possibility that a model with poor performance metrics could have a smaller SD when compared to other models. These exceptions highlight the fact that MLcps takes into account not only the SD but also the overall magnitude of performance metric scores, providing a comprehensive evaluation of ML models' performance.

Consistency in Model Performance across Training and Test Datasets

To evaluate the reliability of MLcps in selecting the best-performing models, we examined the consistency of model performance between the training and test datasets. Among the four datasets, the The Cancer Genome Atlas (TCGA) - Breast Invasive Carcinoma (BRCA) datasets offered a larger sample size, allowing us to create an independent test set comprising 30% of the data. When analyzing these two datasets, we found that the model identified as the best performer based on MLcps also demonstrated the best performance on the independent test set (Figures 2C-F).

Furthermore, it is important to note that if the SD alone was used to rank the models, the LR model would have been selected as the best performer on the training dataset of TCGA-BRCA mRNA (Figure 1D). However, when considering its performance on the test dataset (Figure 2F), LR did not even rank in the top three. In contrast, when sorting the model performance based on MLcps, the ranking remained similar between the training and test datasets (Figures 2C-F). These findings indicate that MLcps effectively identifies models that not only perform well on the training data but also

generalize well to unseen data, highlighting its comprehensive ability to assess model performance across different datasets.

Importance of Using Multiple Performance Metrics

To emphasize the significance of using multiple performance metrics in evaluating ML model performance, we employed a visual representation of the metric scores using a two-dimensional polar coordinate system for each ML algorithm trained on different datasets. Our results demonstrated that both precision and average precision metrics consistently yielded high scores (>90%) for all the trained models in the TCGA miRNA (Figures S1 B-C) and mRNA datasets (Figure S2 B-C). However, relying solely on these metrics would have resulted in mistakenly selecting the dummy model as the best-performing one. This highlights the crucial importance of incorporating multiple performance metrics to obtain a more accurate assessment of ML model performance. Importantly, this phenomenon was not observed in the CLL and cervical cancer datasets (Figure S1A, Figure S2A), indicating that the interpretation of performance metrics is dataset dependent. By considering a diverse range of metrics, researchers and practitioners can make more informed decisions regarding the usefulness and reliability of ML models.

Material and Methods

MLcps Methodology

The MLcps algorithm requires an input table consisting of columns that hold various performance metrics, such as F1, Accuracy, and Recall. The rows in the table represent different machine learning methods, such as K-Nearest Neighbours (KNN) and Support-Vector Machine (SVM). Typically, this table is generated as the output of a standard machine learning pipeline (Figure 3.A - 3.C).

To calculate MLcps, the first step involves plotting the pre-calculated performance metrics on a two-dimensional polar coordinate system (Figure 3.D). In this polar coordinate system, each metric is represented as a ray, and the length of the ray corresponds to the metric value. This representation allows the polar plane to be divided into multiple triangles, with the number of triangles being equal to the available evaluation metrics. The combined area of these individual triangles represents the total area of the polar plane and serves as the MLcps (Figure 3.E).

Finally, the MLcps can be visually represented using a bar chart, as shown in Figure 3.F. It provides a clear and visually informative depiction of the relative performance of different machine learning methods. By examining the bar chart, one can easily identify the performance differences between various ML methods.

Area calculation of a two-dimensional polar plane

The projection of multiple evaluation metrics onto a two-dimensional polar coordinate system divides the polar plane into several triangles. Therefore, the total sum of the areas of these triangles is equal to the total area of the polar plane generated by the multiple performance scores. In order to calculate the area of each individual triangle,

as described in Equation 1, we need to multiply half the length of base by the height drawn to that side (Figure 3.G-N).

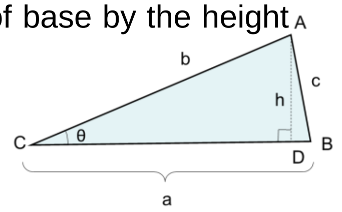

Equation 1: $\text{Area}_{\Delta ABC} = \frac{1}{2}\, ah$

Where:
a = represents the side (base).
h = represents the height drawn to that side.

However, to apply this formula, we require the value for the height (h) variable, which cannot be controlled in a polar plane. Nonetheless, we do have control over the angles (θ) of all the triangles, which can be calculated by dividing 360 degrees by the number of performance metrics used, as described in Equation 2.

Equation 2: $\text{Angle } \theta = \frac{360}{\text{Number of performance metrics}} \times \frac{\pi}{180} = \frac{2\pi}{\text{Number of performance metrics}}$

Now, by employing trigonometry, as outlined in Equation 3, we can calculate the height (h) based on the known angles (θ). Therefore, the height of the triangle can be expressed as $h = b\, sin\, \theta$.

Equation 3: $\sin\theta = \frac{\text{h}}{b}$

By substituting the new expression for the height (h) variable into the general formula for the area of a triangle, we obtain a new formula, as shown in Equation 4, where values for all the required variables are available.

Equation 4: $\text{Area}_{\Delta ABC} = \frac{1}{2}\, ab\text{sin}\theta$ or $2\text{Area}_{\Delta ABC} = absin\theta$

In Equation 4, the parameters a and b represent any two sides of a triangle, while θ denotes the included angle. It is important to note that in this context, the values a and b correspond to the actual measurements for each performance metric.

Finally, by utilizing Equation 5, derived from Equation 4, the total area of the polar plane can be determined by summing the areas of all triangles formed within the polar coordinate system.

Equation 5: $2\text{Area}_{\text{total}} = \sin\theta \sum_{i=1}^{n} d_i d_{i+1} \rightarrow \text{Area}_{\text{total}} = \frac{1}{2}\sin\theta \sum_{i=1}^{n} d_i d_{i+1}$

Where:
d_i = length of the ith ray (the value of ith metric score) (Figure 3L)
n = number of triangles point of collapse (Figure 3M)

Weighted MLcps

In specific situations, certain metrics hold more significance than others. For instance, when dealing with an imbalanced dataset, achieving a high F1 score may be prioritized over higher accuracy [7]. In such cases, users have the option to assign weight variables to the metrics of interest during the calculation of MLcps. A weight variable assigns a value (referred to as the weight) to each pre-computed metric, and the respective metric scores are adjusted using these weights in the following manner:

Equation 6: $S_{\text{weightedmetric}} = S_{\text{metric}} \times W_{\text{metric}}$

Where:

$S_{\text{weightedmetric}}$ = Weighted metric Score
S_{metric} = Raw metric Score
W_{metric} = Weight

It is essential to note that the assigned weight for a metric must always be greater than or equal to zero. A weight of zero indicates that the user intends to exclude that metric from the MLcps calculation. Metrics with higher weights have a more significant contribution to the MLcps compared to metrics with lower weights. In the case where no weights are assigned (unweighted MLcps), it is equivalent to conducting a weighted analysis where all weights are set to 1.

Datasets

In this study, four distinct datasets were employed to evaluate MLcps (Table 1). The initial dataset comprises mRNA data (n=136) derived from a CLL study, which examined transcriptome profiles in individuals affected by blood cancer [8]. Our objective was to develop a model capable of distinguishing between male and female patients using their transcriptomic profiles. To achieve this, we focused on the top 5,000 most variably expressed mRNAs, excluding genes from the Y chromosome.

The second set of data was obtained from a study on cervical cancer, where the expression levels of 714 miRNAs were measured in human samples (n=58) [9]. The third and fourth datasets were collected from TCGA and involved mRNA (n=1219) and miRNA (n=1207) sequencing of BRCA. The TCGAbiolinks package in R was used to retrieve these datasets [10]. For the BRCA mRNA dataset, we focused on genes that showed differential expression according to edgeR analysis (FDR <= 0.001 and logFC > ±2) [11]. Our objective was to develop a model capable of distinguishing between normal and tumor samples for both the cervical cancer and TCGA-BRCA datasets. Among these datasets, two were relatively small (CLL and the cervical cancer study), while the other two were imbalanced (Table 1). We employed an in-house ML pipeline (Figure S3) to train and evaluate eight different models (Table S1) and identify the best-performing one.

Table 1: Example datasets used in this study.

Dataset	Data type	Number of Samples	Number of Features	Target Class ratio
CLL	mRNA	136	5000	Male (n=82): Female (n=54)
Cervical cancer	miRNA	58	714	Normal (n=29): Tumor (n=29)
TCGA-BRCA	miRNA	1207	1404	Normal (n=104): Tumor (n=1104)
TCGA-BRCA	mRNA	1219	5520	Normal (n=113): Tumor (n=1106)

Implementation

MLcps is developed using Python [12] and R [13] programming languages. Pandas [14] is used to store and process the data. Plotly is used to generate the figures. The radarchart [15] package in R was used for surface area calculation of the polar plane.

Conclusion

Our paper introduces MLcps, a new evaluation metric implemented as a Python package. MLcps is a robust evaluation metric specifically designed for classification problems. Its ability to integrate multiple evaluation metrics into a single score makes it an efficient and reliable approach for evaluating model performance and selecting the most successful model. This is especially valuable when multiple evaluation metrics are needed to fully comprehend a model's strengths and weaknesses.

Availability of supporting source code and requirements

Project name: Machine Learning cumulative performance score (MLcps)

Project home page: https://github.com/FunctionalUrology/MLcps

Operating system(s): Platform independent

Programming language: Python >=3.8 and R >=4.0

Other requirements: radarchart, tibble, and dplyr R packages.

License: GNU GPL

Authors' Contributions

K.M., A.H.G, and A.A. conceived the idea for the manuscript. A.A. and M.K. wrote the source code in addition to carrying out testing and debugging of the MLcps. K.M., F.C.B, and A.H.G tested the MLcps and provided scientific inputs throughout the development phase. F.C.B, R.M.A and A.B.A provided the feedback on biological application of the tool. N.S and M.A provided the mathematical support and did the testing and debugging. All authors contributed to writing, proofreading, and correcting the manuscript.

Funding

We gratefully acknowledge the financial support of the Swiss National Science Foundation (SNF Grant 310030_175773 to FCB and KM, 212298 to FCB and AHG) and the Wings for Life Spinal Cord Research Foundation (WFL-AT-06/19 to KM). AHG and RMA are supported by R01 DK 077195 and R01 DK127673. MK is supported by the Else Kröner-Fresenius-Stiftung (EKFS 2021_EKeA.33). The authors acknowledge the financial support from the Federal Ministry of Education and Research of Germany and by the Sächsische Staatsministerium für Wissenschaft Kultur und Tourismus in the program Center of Excellence for AI-research "Center for Scalable Data Analytics and Artificial Intelligence Dresden/Leipzig" (project identification number: ScaDS.AI).

Competing Interests

The authors have declared no competing interests.

Data availability

Not applicable. DOME-ML (Data, Optimisation, Model, and Evaluation in Machine Learning) annotation, supporting the current study, is available through DOME Wizard.

Acknowledgment

We express our sincere gratitude to Dr. Nezhla Aghaei for their invaluable inspiration, assistance in guiding us through the mathematical formulation and providing expert consultation in the calculation of the planar surface area.

References

1. Sun, Y.M., A.K.C. Wong, and M.S. Kamel, *Classification of Imbalanced Data: A Review.* International Journal of Pattern Recognition and Artificial Intelligence, 2009. **23**(4): p. 687-719.
2. Huang, J. and C.X. Ling, *Using AUC and accuracy in evaluating learning algorithms.* Ieee Transactions on Knowledge and Data Engineering, 2005. **17**(3): p. 299-310.
3. Huang, J., J.J. Lu, and C.X. Ling, *Comparing naive bayes, decision trees, and SVM with AUC and, accuracy.* Third Ieee International Conference on Data Mining, Proceedings, 2003: p. 553-556.
4. Provost, F. and P. Domingos, *Tree induction for probability-based ranking.* Machine Learning, 2003. **52**(3): p. 199-215.
5. Racz, A., D. Bajusz, and K. Heberger, *Multi-Level Comparison of Machine Learning Classifiers and Their Performance Metrics.* Molecules, 2019. **24**(15).
6. Branco, P., L. Torgo, and R.P. Ribeiro, *A Survey of Predictive Modeling on Im balanced Domains.* Acm Computing Surveys, 2016. **49**(2).
7. Galar, M., et al., *A Review on Ensembles for the Class Imbalance Problem: Bagging-, Boosting-, and Hybrid-Based Approaches.* Ieee Transactions on Systems Man and Cybernetics Part C-Applications and Reviews, 2012. **42**(4): p. 463-484.
8. Dietrich, S., et al., *Drug-perturbation-based stratification of blood cancer.* Journal of Clinical Investigation, 2018. **128**(1): p. 427-445.
9. Witten, D., et al., *Ultra-high throughput sequencing-based small RNA discovery and discrete statistical biomarker analysis in a collection of cervical tumours and matched controls.* Bmc Biology, 2010. **8**.
10. Colaprico, A., et al., *TCGAbiolinks: an R/Bioconductor package for integrative analysis of TCGA data.* Nucleic Acids Research, 2016. **44**(8).
11. Robinson, M.D., D.J. McCarthy, and G.K. Smyth, *edgeR: a Bioconductor package for differential expression analysis of digital gene expression data.* Bioinformatics, 2010. **26**(1): p. 139-140.
12. Van Rossum, G., & Drake, F. L. , *Python 3 Reference Manual.* 2009.
13. Team, R.C., *R: A language and environment for statistical computing. R Foundation for Statistical Computing.* 2013.
14. McKinney, *Data Structures for Statistical Computing in Python.* Proceedings of the 9th Python in Science Conference,, 2010.
15. Porter, D.A.a.S., *radarchart: Radar Chart from 'Chart.js'. R package version 0.3.1.* 2016.

Figure
Click here to access/download;Figure;main.pdf

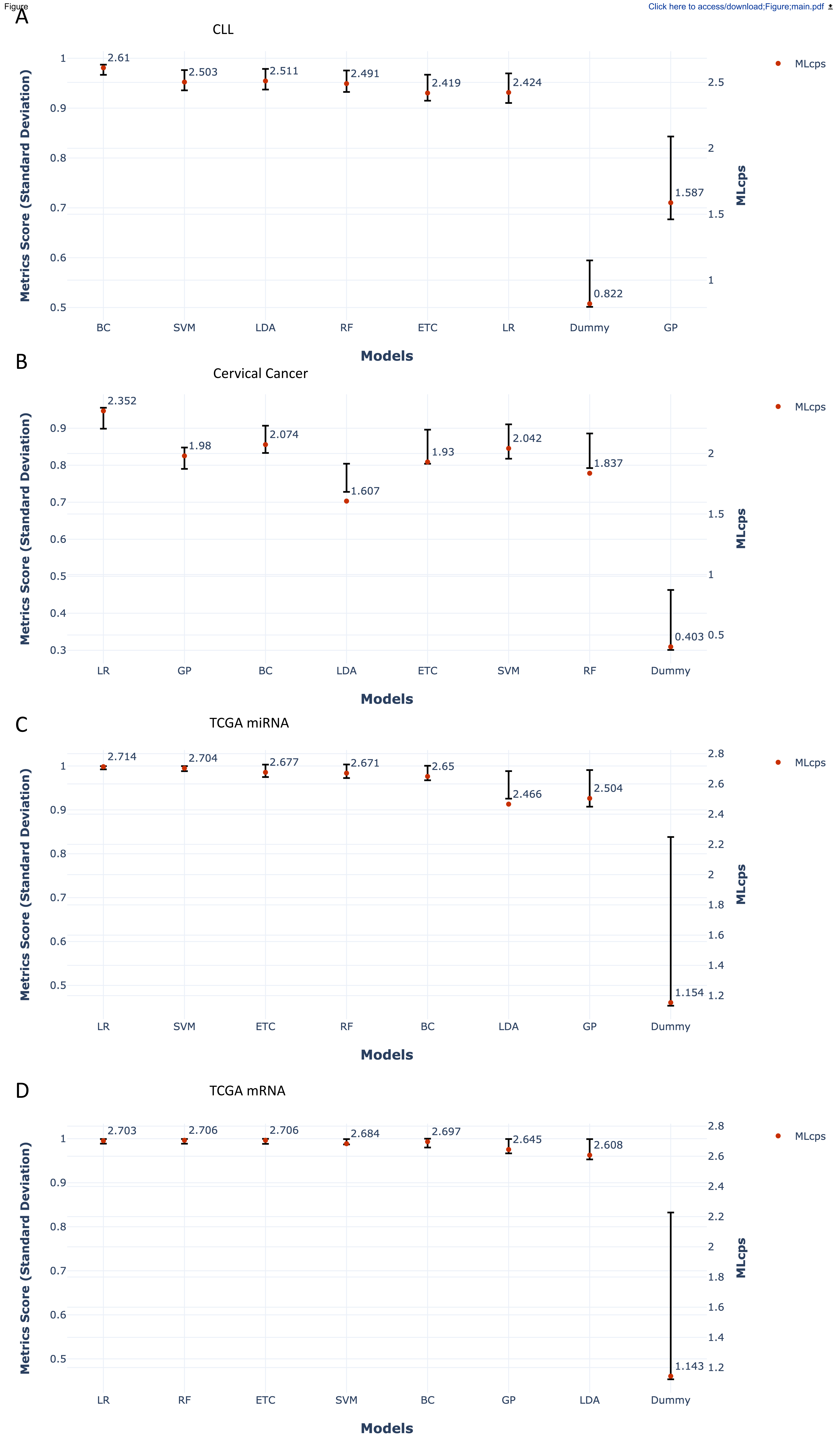

Figure 1. Standard deviation (SD) of performance metrics score of ML algorithms trained on different example datasets. The plots in the figure represent A) CLL dataset, B) Cervical cancer dataset, C) TCGA miRNA dataset, and D) TCGA mRNA dataset. Each bar in the plot represents the standard deviation (SD) of the performance metric scores, shown on the left y-axis. The bars are arranged from left to right, with smaller SD on the left and larger SD on the right. Furthermore, a red dot is placed on the plot to represent MLcps, which is displayed on the right y-axis.

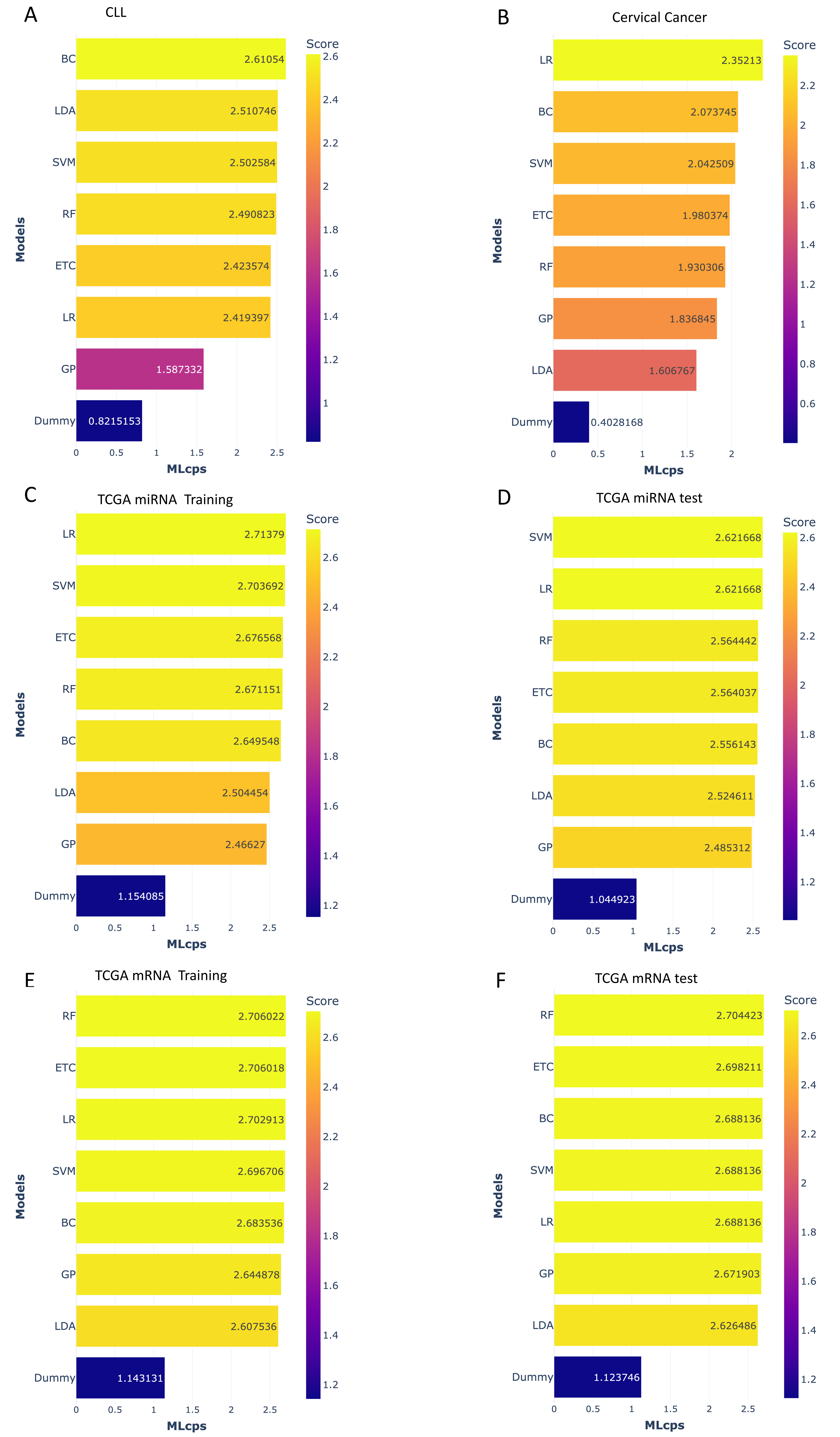

Figure 2 Bar charts visualizing the MLcps for different example datasets. Plots are representing A) CLL dataset, B) Cervical cancer dataset, C) TCGA miRNA training dataset, D) TCGA miRNA test dataset, E) TCGA mRNA training dataset, and F) TCGA mRNA test dataset. The color and the length of the bars represent the MLcps. Darker colors indicate lower scores, while lighter colors indicate higher scores.

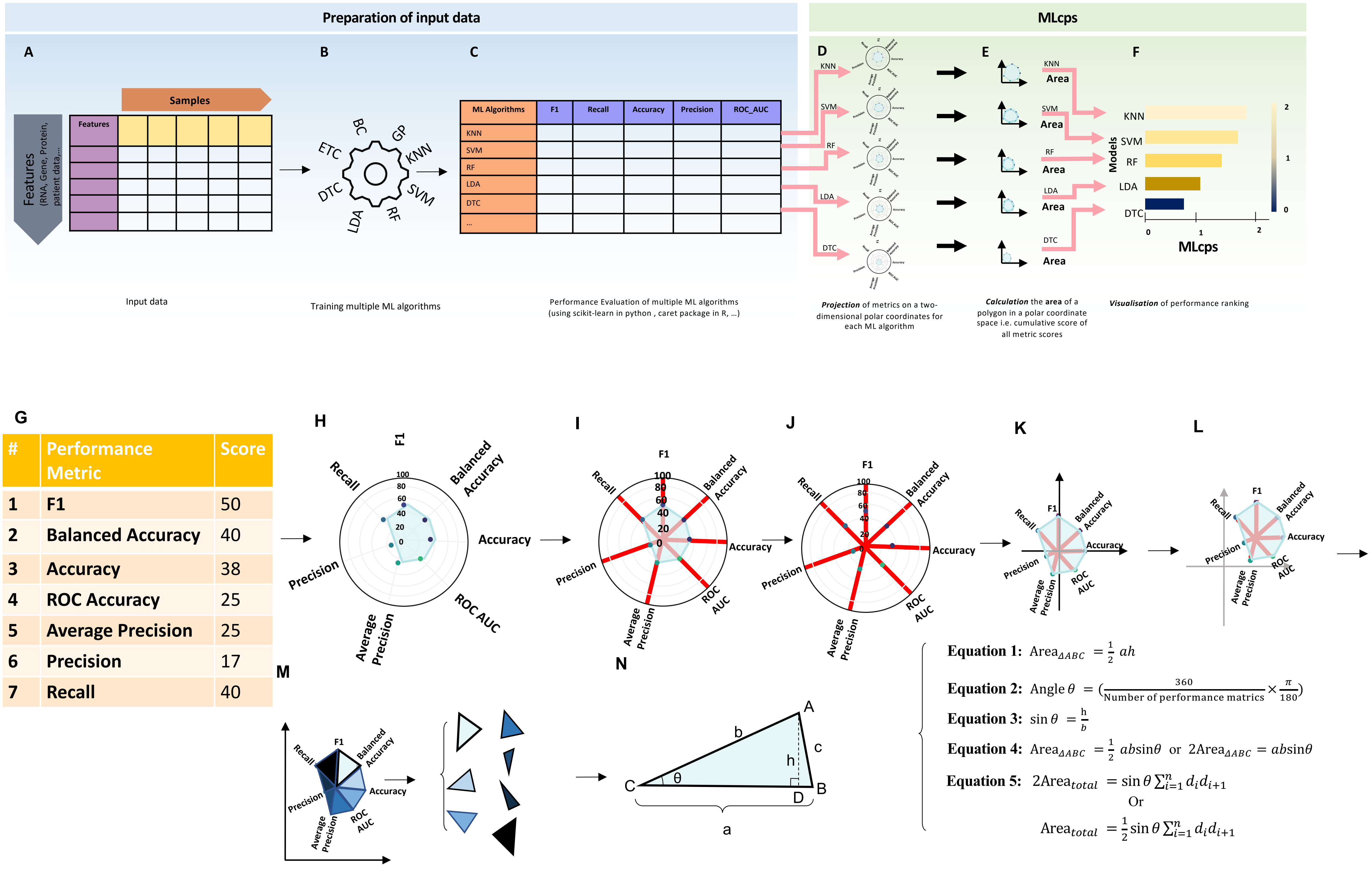

Figure 3 Schematic overview of the complete analysis process for MLcps Python package. Before using the MLcps Python package, one needs to prepare the raw data (A). This input table can be RNA sequencing, proteomics, patients' profile, molecular data, etc (normally this data is in txt or csv format). Next step is to perform multiple ML algorithms (B). Performing this step can be done by any package or programming language of choice. The next step is to evaluate the performance of the ML algorithms. We recommend the use of multiple metrics such as F1, Recall, etc (C). The performance metric scores then need to be arranged in a tabular format as depicted in (C). This table will be used as an input for the MLcps package. From here on the MLcps will process the data. MLcps involves three steps: projection, calculation, and visualization (PCV). To calculate the cumulative score of each ML algorithm in the input data, MLcps first projects the performance metric onto the two-dimensional polar coordinates system (D). Next, the projected polygon's area is calculated (E). Finally, the user can visualize this MLcps to rank the performance of given ML algorithms (F). The lower panel (G-N) visualises the procedure to calculate the surface area as cumulative score in detail. The names of the algorithms are just mentioned as example and other algorithm can be used too. ETC: Extra Trees Classifier, BC: Bagging Classifier, GP: Gaussian Process Classifier, KNN: K-Nearest Neighbors, SVM: Support Vector Machine, RF: Random Forest Classifier, LDA: Linear Discriminant Analysis, DTC: Decision Tree Classifier.

Click here to access/download
Supplementary Material
suppl.docx

Supplementary Figures

Click here to access/download
Supplementary Material
suppl.pdf
