# Supplementary material for: MLcps: machine learning cumulative performance score for classification problems
Source: Gigascience. 2023 Dec 13;12:giad108. doi: 10.1093/gigascience/giad108 (PMC10716825; doi:10.1093/gigascience/giad108)
Supplement: giad108_GIGA-D-23-00187_Revision_1 [file giad108_giga-d-23-00187_revision_1.pdf]

GigaScience

MLcps: Machine Learning Cumulative Performance Score for Classification Problems

--Manuscript Draft--

**Manuscript Number:**	GIGA-D-23-00187R1
**Full Title:**	MLcps: Machine Learning Cumulative Performance Score for Classification Problems
**Article Type:**	Technical Note
**Funding Information:**	Schweizerischer Nationalfonds zur Förderung der Wissenschaftlichen Forschung (310030_175773) — Prof Fiona C. Burkhard<br>Schweizerischer Nationalfonds zur Förderung der Wissenschaftlichen Forschung (212298) — Prof Fiona C. Burkhard<br>Wings for Life (WFL-AT-06/19) — Prof Katia Monastyrskaya<br>Else Kröner-Fresenius-Stiftung (EKFS 2021_EKeA.33) — Not applicable<br>Sächsisches Staatsministerium für Wissenschaft und Kunst (ScaDS.AI) — Not applicable
**Abstract:**	Background<br>Assessing the performance of machine learning (ML) models requires careful consideration of the evaluation metrics used. It is often necessary to utilize multiple metrics to gain a comprehensive understanding of a trained model's performance, as each metric focuses on a specific aspect. However, comparing the scores of these individual metrics for each model to determine the best-performing model can be time-consuming and susceptible to subjective user preferences, potentially introducing bias.<br>Results<br>We propose Machine Learning Cumulative Performance Score (MLcps), a novel evaluation metric for classification problems. MLcps integrates several pre-computed evaluation metrics into a unified score, enabling a comprehensive assessment of the trained model's strengths and weaknesses. We tested MLcps on four publicly available datasets, and the results demonstrate that MLcps provides a holistic evaluation of the model's robustness, ensuring a thorough understanding of its overall performance.<br>Conclusion<br>By utilizing MLcps, researchers and practitioners no longer need to individually examine and compare multiple metrics to identify the best-performing models. Instead, they can rely on a single MLcps value to assess the overall performance of their ML models. This streamlined evaluation process saves valuable time and effort, enhancing the efficiency of model evaluation. MLcps is available as a python package at https://pypi.org/project/MLcps/ and examples of its use can be found at https://mybinder.org/v2/gh/FunctionalUrology/MLcps.git/main.
**Corresponding Author:**	Ali Hashemi Gheinani<br>Harvard Medical School<br>SWITZERLAND
**Corresponding Author Secondary Information:**	
**Corresponding Author's Institution:**	Harvard Medical School
**Corresponding Author's Secondary Institution:**	
**First Author:**	Akshay Akshay
**First Author Secondary Information:**	
**Order of Authors:**	Akshay Akshay<br>Masoud Abedi

Powered by Editorial Manager® and ProduXion Manager® from Aries Systems Corporation

Navid Shekarchizadeh

Fiona C. Burkhard

Mitali Katoch

Alex Bigger-Allen

Rosalyn M. Adam

Katia Monastyrskaya

Ali Hashemi Gheinani

Order of Authors Secondary Information:

Response to Reviewers:

######################
Reviewer #1
######################

<<---- Reviewer comment ---->>
Reviewer #1: Overall, I like the author's idea to combine metrics as another way to conduct model evaluation, in a way that takes both the magnitude and standard deviation of metrics scores into account. I think it's great that the authors have implemented this and shared it via gitub as an installable package. It's an interesting and potentially useful idea, but I believe this paper really needs to be expanded and improved before publication based on the comments laid out below.

! °°°° Response to Reviewer comment °°°°!
Thank you for taking the time to review our paper and for your valuable feedback. We appreciate your positive comments about MLcps. We understand your concerns about the need for further expansion and improvement before publication, and we are committed to addressing them to in the following text and manuscript to enhance the quality of our work. Your feedback is instrumental in helping us refine our manuscript. Below, you will find our response to the concerns you raised.

<<---- Reviewer comment ---->>
-This paper makes a lot of assumptions about how classification metrics are used to pick a best performing model. One being that minimizing standard deviation between metrics is always best, if this is the case, some citation or demonstration of this would be expected. In my experience, an model that maximizes some specific metric is often most desirable even at the expense of another metric not performing as well. This in itself is the foundation of using different decision thresholds in a model to (for example) maximize False Negatives for a given modeling task even at the expense of increased False Positives. Also while I agree that examinine multiple metrics is essential for picking a best model, creating a single meta-metric seems fundamentally risky and likely to lead to unforeseen issues in picking a best model under different use cases. This paper does not address this possibility.

! °°°° Response to Reviewer comment °°°°!
We appreciate your insightful feedback.
We'd like to clarify that we did not assert that minimizing standard deviation is the only optimal method for selecting the best-performing model. Rather the whole idea behind suggesting MLcps is to cautiously suggest another cumulative score that can compensate the shortcomings of SD. In this manuscript, we highlight that SD is one of the criteria commonly used in the field for such a purpose. We have also explicitly shown why it may not always be the most appropriate choice. We introduced the standard deviation criterion to emphasize how MLcps can identify the truly best performing model, even in situations, where relying solely on standard deviation criteria for model selection proves inadequate (please refer to lines 189-218).

We totally agree with your second comment, which underscores the standard practice of prioritizing specific metrics depending on the domain or stakeholders. Therefore, we have already integrated a weighting feature into MLcps, enabling users to specify which metric holds greater value for them. MLcps will then take this preference into account during the calculation process. For example, in some medical diagnosis tasks, sensitivity (true positive rate) might be more critical than specificity (true negative rate).

Powered by Editorial Manager® and ProduXion Manager® from Aries Systems Corporation

Please refer to the lines 342-365.

Regarding the comment about the risks associated with combining metric scores, while there are valid concerns about creating a single meta-metric, the key is to use MLcps judiciously and in conjunction with domain knowledge. It's not a replacement for individual metrics or domain-specific considerations but rather a tool to aid in comprehensive model evaluation. When used wisely, it can provide a valuable additional perspective on model performance, especially in cases where multiple stakeholders with different metric preferences are involved.

We cannot envision a use case where MLcps inherently poses risks, as it combines precomputed metrics into a unified score. However, it's essential to understand that MLcps is only as good as the metrics used to calculate it. Recognizing which metrics are relevant to your models, a consideration influenced by factors such as domain, stakeholder preferences, and data characteristics, remains of paramount importance. Your comment has prompted us to emphasize this point to our readers and users. As a result, we have expanded the conclusion section to underscore the significance of selecting an appropriate evaluation metric based on specific domain or problem. Please refer to lines 425-436.

<<---- Reviewer comment ---->>
-Many of the figures are not using the space effectively to communicate the results. I'd suggest considering other ways to present/visualize these results more clearly. Figure 2 in particular is not a very effective way to clearly illustrate consistency in performance between training and testing sets on the individual datasets.

! °°°° Response to Reviewer comment °°°°!
We appreciate your input and have made significant changes based on your suggestions. We have revamped Figure 2 (previously) to improve clarity and space utilization. By combining the training and testing scores into a single plot, we have created a more concise representation of model performance. Additionally, we have incorporated model performance ranking information directly within the plot to enhance the comprehensibility of the results. We believe these modifications will better convey the consistency in performance between the training and testing sets across different datasets. Furthermore, we have updated the figure order. We have grouped all the plots related to CLL and Cervical datasets into figure 1, and the TCGA-related plots into figure 2. We believe these amendments will address your concerns effectively.

<<---- Reviewer comment ---->>
-The paper order of sections makes it difficult to understand the content presentation. I found it necessary to jump between sections to understand what the authors were doing and to try and understand their arguments.

! °°°° Response to Reviewer comment °°°°!
To address this issue, we have reorganized the order of the figures in the paper to enhance the logical flow of information. This adjustment should make it easier for readers to follow the sequence of our arguments and grasp the context. Additionally, we have incorporated introductory text into the results section. This introductory text is included to provide readers with a clear understanding of what to anticipate in the results section, thus facilitating a smoother transition between sections (lines 173-185).

We believe these changes will significantly enhance the readability and coherence of our paper, enabling readers to more effectively follow our arguments and gain a better understanding of the content.

<<---- Reviewer comment ---->>
-One of the points/observations of this paper is that low SD alone would not be a good meta metric, which seems like an obvious conclusion that could have been drawn without these experiments. I.e. a poor model with consistently low evaluation metric scores would have a low SD

! °°°° Response to Reviewer comment °°°°!
Thank you for your comment. We included this information to illustrate that MLcps,

Powered by Editorial Manager® and ProduXion Manager® from Aries Systems Corporation

unlike SD criteria, can handle these kinds of situations and can help identify the truly best-performing model.

<<---- Reviewer comment ---->>
-The comparison of training and testing scores for the metric doesn't seem very useful given that training evaluations are largely not useful to begin with (as they really only indicate if an algorithm is learning anything or not from the data, whether it be signal or noise). Thus differences in metric values really only reflect potential overfitting which is a reflection on the algorithm rather than the metric.

! °°°° Response to Reviewer comment °°°°!
We agree about the limitations of relying solely on training results, and we appreciate your insight. In certain research domains, like the biological field with Proteomics/metabolomics/RNA-Seq data, obtaining a substantial amount of data can be particularly challenging due to sample scarcity. As a result, researchers often resort to using all available data for training and employ evaluation methods such as k-fold cross-validation to assess model performance.

In such situations, the luxury of having an independent test dataset to cross-check for overfitting may not be available. It's in these scenarios that we emphasize the importance of our findings. We showcased that MLcps tends to favor models that demonstrate robust generalization, not only excelling on the training data but also performing well on unseen datasets. This observation underscores the significance of using a comprehensive metric like MLcps, especially when researchers are compelled to rely on training data for their evaluations.

<<---- Reviewer comment ---->>
-Which metrics were included in MLcps, is not really addressed in the body of the paper and only appears to be laid out in Figure 3. It sounds like the authors are suggesting that the user can pick any number of initial metrics they want to include in MLcps, however it is not made explicitly clear which metrics were used in this papers's experiments (presumably the ones specified in Figure 3). This study would have benefitted from more specificity for reproducibility of these results, and further if the authors are suggesting that MLcps can be applied with whatever metrics a user wants to combine into the meta-metric, this paper should included evaluations examining different numbers and combinations of metrics into the MLcps calculation. How does the selection of different metrics and the diversity of what these metrics are good at capturing impact the MLcps scoring?

! °°°° Response to Reviewer comment °°°°!

Thank you for your comment. We would like to clarify that MLcps can be calculated using any evaluation metric. It is indeed a key aspect of MLcps that users have the flexibility to select metrics that align with their specific classification problem. However, it is highly recommended that all metrics are on the same scale (Normalised). For example, if accuracy ranges from 0 to 1, then the F1 metric should also be within the same range, rather than in percentage format. We have also included this information in the manuscript. Please refer to lines 271-274 for the details. Figure 3 is using a Schematic to describe the complete analysis process for MLcps Python package and in panel G we mentioned that for example F1, Jaccard score, Accuracy, ROC Accuracy, Average Precision, Precision, Recall can be used.

Regarding the exploration of "different numbers and combinations of metrics into the MLcps calculation," please refer to Figure S2, which showcases the use of a comprehensive set of seven distinct metrics, and Figure S3, where MLcps calculation is performed using five different metrics.

Figure S2: This figure presents the projection of metric scores onto a two-dimensional (2D) polar coordinate system for multiple machine learning algorithms trained on different example datasets, incorporating a total of seven metrics.

Figure S3: Similarly, Figure S3 provides a visual representation of metric scores projected onto a two-dimensional (2D) polar coordinate system, this time utilizing a set

Powered by Editorial Manager® and ProduXion Manager® from Aries Systems Corporation

of five different metrics. These figures not only demonstrate the flexibility of MLcps but also highlight its adaptability to diverse metric combinations, ensuring robust performance evaluation across various datasets and scenarios.

<<---- Reviewer comment ---->>
-Furthermore, in Figure 3, it doesn't make any sense to include both accuracy and balanced accuracy into this cumulative score (as it is effectively weighting accuracy twice, since accuracy = balanced accuracy when the class balance is equal, and standard accuracy is a biased metric to begin with in contrast with balanced accuracy when the class balance is not equal.

! °°°° Response to Reviewer comment °°°°!

Thank you for your comment. Figure 3 is intended to illustrate the methodology of MLcps, and the metrics used are merely examples. However, to alleviate any potential confusion and address your concern, we have implemented your suggestion by replacing balanced accuracy with the Jaccard score in Figure 3.

<<---- Reviewer comment ---->>
-Metric weighting is discussed in this paper, but no evaluations or discussion of the risks of weighting is given.

! °°°° Response to Reviewer comment °°°°!
Thank you for your comment. We have extended the conclusion section to discuss the potential drawbacks associated with weighting. Please refer to lines 438-443 .

<<---- Reviewer comment ---->>
-Given the potential generalizability of this topic to machine learning problems in all sorts of application domains, it would be much more convincing to see analyses conducted on a much broader range of target problems/datasets (i.e. outside of the just the 4 RNA datasets presented).

! °°°° Response to Reviewer comment °°°°!

To demonstrate the generalizability of MLcps, we have included another dataset consisting of biometric information from 100,000 individuals, gathered from the National Health Insurance Service in Korea (line 385-397). Our objective was to predict alcohol consumption status using this extensive biometric dataset. We have observed a consistent trend in the results with this dataset as well (line 208,235-242).

<<---- Reviewer comment ---->>
-I believe there must be other related work dealing with combining evaluation metrics that the authors should cite and contrast their approach with. It would also be interesting for them to discuss metrics that evaluate a model using the same decision threshold vs. metrics that consider a range of decision thresholds (e.g. ROC AUC). Can this approach combine both? What would the implication of this be?

! °°°° Response to Reviewer comment °°°°!
Thank you for your valuable feedback.

We have conducted a thorough literature review and identified two studies as the most closely related work to our approach. In their studies, they employed a similar methodology to enhance Information Retrieval (IR) metrics by combining multiple evaluation measures (lines 155-159). It's worth noting that their work primarily outlined the methodology without providing a direct implementation, which limits our ability to make a direct comparison with MLcps. Nevertheless, apart from this study, we have not encountered any other research that introduces analogous methods for metric combination within classification domain.

Regarding your second comment, we appreciate your suggestion to incorporate both single-threshold and multi-threshold metrics MLcps calculation. While it is feasible to include both types of metrics in the calculation of MLcps, it is important to note that, as it stands, MLcps currently does not involve threshold criteria during its computation and does not provide control over thresholds. This limitation is primarily due to the nature of

Powered by Editorial Manager® and ProduXion Manager® from Aries Systems Corporation

MLcps, which relies on precomputed metrics. Adjusting thresholds is typically a part of model training, and MLcps operates on the basis of these precomputed metrics. Although the inclusion of threshold control is an intriguing idea, it may fall outside the scope of our current manuscript, as MLcps is specifically designed to work with precomputed metrics and does not engage in threshold manipulation during its calculation. We appreciate your thought-provoking suggestion and will consider it for future research endeavors.

######################
Reviewer #2
######################

<<---- Reviewer comment ---->>
Reviewer #2: This paper presents machine learning cumulative performance score for classification problems. The authors proposed an evaluation metric used in classification. The authors integrated several pre-computed evaluation metrics in to an unified score. The authors tested their proposed system on four types of publicly available datasets. The authors used 15 reference papers considering the years 2003-2018. The authors should increse the number of references to be used for comparison purposes and the authors should consider recent year publications.

! °°°° Response to Reviewer comment °°°°!
We sincerely appreciate your time and effort in reviewing our paper, and we value your constructive feedback. In response to your suggestion, we have expanded our reference list to include recent studies that address various aspects of model evaluation, such as the use of multiple evaluation metrics and the impact of subject bias.

Regarding comparing MLcps, we have conducted a thorough literature review and identified two studies as the most closely related work to our approach. In their studies, they employed a similar methodology to enhance Information Retrieval (IR) metrics by combining multiple evaluation measures (lines 155-159). It's worth noting that their work primarily outlined the methodology without providing a direct implementation, which limits our ability to make a direct comparison with MLcps. Nevertheless, apart from this study, we have not encountered any other research that introduces analogous methods for metric combination within classification domain.

**Additional Information:**	
**Question**	**Response**
Are you submitting this manuscript to a special series or article collection?	No
**Experimental design and statistics**<br><br>Full details of the experimental design and statistical methods used should be given in the Methods section, as detailed in our Minimum Standards Reporting Checklist. Information essential to interpreting the	Yes

Powered by Editorial Manager® and ProduXion Manager® from Aries Systems Corporation

data presented should be made available in the figure legends.<br><br>Have you included all the information requested in your manuscript?	
**Resources**<br><br>A description of all resources used, including antibodies, cell lines, animals and software tools, with enough information to allow them to be uniquely identified, should be included in the Methods section. Authors are strongly encouraged to cite Research Resource Identifiers (RRIDs) for antibodies, model organisms and tools, where possible.<br><br>Have you included the information requested as detailed in our Minimum Standards Reporting Checklist?	Yes
**Availability of data and materials**<br><br>All datasets and code on which the conclusions of the paper rely must be either included in your submission or deposited in publicly available repositories (where available and ethically appropriate), referencing such data using a unique identifier in the references and in the "Availability of Data and Materials" section of your manuscript.<br><br>Have you have met the above requirement as detailed in our Minimum Standards Reporting Checklist?	Yes

Manuscript-Revised Click here to access/download;Manuscript;main_KM230705.pdf

MLcps: Machine Learning Cumulative Performance Score for Classification Problems

Akshay Akshay [1,2], Masoud Abedi[3], Navid Shekarchizadeh[3,4], Fiona C. Burkhard[1,5], Mitali Katoch[6], Alex Bigger-Allen [7,8,9,10], Rosalyn M. Adam [8,9,10], Katia Monastyrskaya [1, 5] and Ali Hashemi Gheinani [1,5,8,9,10*]

[1] Functional Urology Research Group, Department for BioMedical Research DBMR, University of Bern, Switzerland

[2] Graduate School for Cellular and Biomedical Sciences, University of Bern, Switzerland

[3]Department of Medical Data Science, Leipzig University Medical Centre, 04107 Leipzig, Germany

[4]Center for Scalable Data Analytics and Artificial Intelligence (ScaDS.AI) Dresden/Leipzig, 04105 Leipzig, Germany

[5] Department of Urology, Inselspital University Hospital, 3010 Bern, Switzerland

[6] Institute of Neuropathology, Universitätsklinikum Erlangen, Friedrich-Alexander-Universität Erlangen-Nürnberg (FAU), Erlangen, Germany

[7] Biological & Biomedical Sciences Program, Division of Medical Sciences, Harvard Medical School, Boston, MA.

[8] Urological Diseases Research Center, Boston Children's Hospital, MA, USA

[9] Harvard Medical School, Boston, Department of Surgery MA, USA

[10] Broad Institute of MIT and Harvard, Cambridge, MA, USA

* Corresponding author:

Ali Hashemi Gheinani, Urological Diseases Research Center, Boston Children's Hospital, Harvard Medical School and Broad Institute of MIT and Harvard, Cambridge, MA, USA

e-mail: Ali.HashemiGheinani@childrens.harvard.edu

Keywords

- Machine Learning
- Classification Problems
- Model Evaluation
- Unified Evaluation Score
- Python Package

Key Points

- Evaluating machine learning models involves considering multiple metrics. Comparing scores of individual metrics to determine the best model can be time-consuming and subjective, potentially introducing bias.

- The proposed Machine Learning Cumulative Performance Score (MLcps) is a novel evaluation metric for classification problems. It integrates multiple evaluation metrics into a unified score, providing a holistic understanding of model performance.

- MLcps outperforms standard metric-based rankings, offering a more reliable and consistent assessment of model performance.

- MLcps is available as a Python package, making it easily accessible for researchers to incorporate into their evaluation pipelines.

Abstract

Background

Assessing the performance of machine learning (ML) models requires careful consideration of the evaluation metrics used. It is often necessary to utilize multiple metrics to gain a comprehensive understanding of a trained model's performance, as each metric focuses on a specific aspect. However, comparing the scores of these individual metrics for each model to determine the best-performing model can be time-consuming and susceptible to subjective user preferences, potentially introducing bias.

Results

We propose Machine Learning Cumulative Performance Score (MLcps), a novel evaluation metric for classification problems. MLcps integrates several pre-computed evaluation metrics into a unified score, enabling a comprehensive assessment of the trained model's strengths and weaknesses. We tested MLcps on four publicly available datasets, and the results demonstrate that MLcps provides a holistic evaluation of the model's robustness, ensuring a thorough understanding of its overall performance.

Conclusion

By utilizing MLcps, researchers and practitioners no longer need to individually examine and compare multiple metrics to identify the best-performing models. Instead, they can rely on a single MLcps value to assess the overall performance of their ML models. This streamlined evaluation process saves valuable time and effort, enhancing the efficiency of model evaluation. MLcps is available as a python package at https://pypi.org/project/MLcps/ and examples of its use can be found at https://mybinder.org/v2/gh/FunctionalUrology/MLcps.git/main.

Introduction

The evaluation of machine learning (ML) models is crucial in the ML workflow as it helps determine their effectiveness. However, it is essential to select the appropriate evaluation metric since the performance of a trained model is only as good as the metric used for evaluation[1–5]. Numerous metrics are available for assessing the performance of ML models, with each metric focusing on a specific aspect of the model's performance[6,7]. For example, the "recall" metric effectively measures a model's ability to predict positive class instances but does not provide insights into the negative class instances. This poses a significant challenge because a model that performs well according to one metric may not exhibit the same level of performance when evaluated using another metric[8–14]. Hence, relying solely on a single performance metric is inadequate in practical scenarios.

Furthermore, the characteristics and composition of the available dataset can influence the behaviour and outcomes of various metrics. For instance, when dealing with imbalanced datasets, accuracy becomes an inadequate metric, and relying solely on accuracy can lead to misleading interpretations[15]. Therefore, it is crucial to calculate multiple performance metrics for each model to evaluate its performance comprehensively[7]. By considering various evaluation metrics, we can gain a holistic view of a model's performance and make informed decisions about the best-performing model for a given task.

When calculating multiple metrics for a model, there is often an assumption that the best model will consistently achieve the highest scores across all metrics. However, this assumption is rarely true in practical scenarios, necessitating the comparison of the individual metrics of different models to identify the best-performing model. However, comparing metric scores for many models can be labour-intensive and susceptible to user preference bias[16]. As a result, the complexity of finding the best model increases exponentially when considering the comparison of different metrics.

Apart from these limitations, some methods prevent users from evaluating model performance with multiple metrics simultaneously. For example, in the field of biology, the wrapper-based feature selection method is commonly used to identify important features from a large set of original attributes. This method trains a model with different feature subsets and selects the subset that shows the best performance compared to the other subsets. Unfortunately, these methods are limited to evaluating model performance using only one metric at a time. This constraint can potentially lead to overfitting to a specific metric, resulting in the selection of suboptimal feature subsets that lack generalizability.

In the realm of Information Retrieval (IR), Chakrabarti et al. previously introduced novel algorithms designed to merge multiple ranking criteria into a unified approach, ultimately enhancing the optimization of search results[17]. Building upon this research, Geng et al. further investigated learning to rank, considering multiple evaluation metrics, and proposed the combination of multiple metrics to optimize IR metrics[18].

Here, we introduce a novel evaluation metric called the Machine Learning Cumulative Performance Score (MLcps) to address the challenges associated with model evaluation in the

field of machine learning. MLcps is a unified score that follows a similar methodology compared to the previously mentioned study related to IR. MLcps combines precomputed performance metrics into a single score while preserving their distinct characteristics. By leveraging multiple metrics, MLcps provides a more comprehensive evaluation of machine learning model performance. To enhance the accessibility of MLcps, we have implemented it as a Python package, enabling direct comparisons of trained ML models to assess their performance.

Results and Discussion

In this section, the results of the current study are showcased, with a specific focus on evaluating MLcps as a robust measure for assessing ML model performance. The primary objective of this analysis is to shed light on the effectiveness of MLcps in ranking models based on their consistency and excellence across multiple performance metrics. Furthermore, we explore the reliability of MLcps in selecting models that not only excel on training data but also demonstrate the ability to generalize well to unseen datasets.

Additionally, we emphasize the importance of employing a diverse set of performance metrics when evaluating machine learning models. By doing so, we aim to provide a comprehensive understanding of model performance beyond traditional measures and showcase the significance of considering various aspects of model behaviour in real-world applications.

Evaluating MLcps Robustness

Each performance metric represents a specific aspect of model performance, and for a model to be considered robust and superior, it should consistently excel across all these metrics. This consistency can be reflected by having the lowest standard deviation (SD) across performance metrics. Therefore, our analysis revolves around understanding the relationship between MLcps and SD. This evaluation helps determine the reliability of MLcps as a performance measure.

To assess MLcps' robustness as a model performance measure, we analyzed multiple models across five distinct datasets (Table 1). Our findings consistently revealed a strong correlation between the highest MLcps score and the lowest standard deviation (SD) in performance metric scores (Figures 1A-B and 2A-B). This correlation indicates that MLcps reliably identifies the best-performing model when it consistently excels across all metrics, validating its reliability as a performance measure.

However, there are important exceptions that require attention. For instance, in the Chronic Lymphocytic Leukemia (CLL) dataset, the GP model outperforms the Dummy model in terms of MLcps score, even though the Dummy model has a lower SD (Figure 1A). Similarly, in the cervical cancer dataset, the MLcps scores of the ETC, SVM, and RF models surpass that of the LDA model, despite the LDA model having a lower SD (Figure 1B). Similar exceptions were observed in the body signals dataset as well (Figure S4.A).

These exceptions can be attributed to the fact that while these models exhibit lower SD compared to others, they also perform poorly for each individual metric.

Consequently, their low MLcps scores accurately reflect their subpar performance across all metrics. This observation acknowledges that a model with poor performance metrics may still have a smaller SD when compared to other models. These exceptions underscore that MLcps takes into account not only the SD but also the overall magnitude of performance metric scores, thereby providing a comprehensive evaluation of ML models' performance.

Consistency in Model Performance across Training and Test Datasets

To evaluate the reliability of MLcps in selecting the best-performing models, we examined the consistency of model performance between the training and test datasets. Among the five datasets, the The Cancer Genome Atlas (TCGA) - Breast Invasive Carcinoma (BRCA) and Body Signals datasets offered a larger sample size, allowing us to create an independent test set comprising 30% of the data. When analyzing these three datasets, we found that the model identified as the best performer based on MLcps also demonstrated the best performance on the independent test set (Figures 2C-D).

Furthermore, it is noteworthy that if we solely relied on the SD to rank the models, the LR model would have been chosen as the best performer on the training dataset of TCGA-BRCA mRNA (Figure 2B). However, when evaluating its performance on the test dataset, LR did not even rank among the top two (Figure 2D). Similarly, in the Body Signal dataset, the Bagging Classifier model would have been considered the best performer based on the SD criteria (Figure S4.A). However, it's important to note that on the test dataset, this model ranked fourth in terms of performance (Figure S4.B).

In contrast, when sorting the model performance based on MLcps, the ranking remained consistent across both training and test datasets, providing a more robust measure of model performance (Figure S4.B). These findings indicate that MLcps effectively identifies models that not only perform well on the training data but also generalize well to unseen data, highlighting its comprehensive ability to assess model performance across different datasets.

Importance of Utilizing Multiple Performance Metrics

To emphasize the significance of using multiple performance metrics in evaluating ML model performance, we employed a visual representation of the metric scores using a two-dimensional polar coordinate system for each ML algorithm trained on different datasets. Our results demonstrated that both precision and average precision metrics consistently yielded high scores (>90%) for all the trained models in the TCGA miRNA (Figures S1 B-C) and mRNA datasets (Figure S2 B-C). However, relying solely on these metrics would have resulted in mistakenly selecting the dummy model as the best-performing one. This highlights the crucial importance of incorporating multiple performance metrics to obtain a more accurate assessment of ML model performance. Importantly, this phenomenon was not observed in the CLL and cervical cancer datasets (Figure S1.A, Figure S2.A), indicating that the interpretation of performance metrics is dataset dependent. By considering a diverse range of metrics, researchers and practitioners can make more informed decisions regarding the usefulness and reliability of ML models.

Material and Methods

MLcps Methodology

The MLcps algorithm requires an input table consisting of columns that hold various performance metrics, such as F1, Accuracy, and Recall. The rows in the table represent different machine learning methods, such as K-Nearest Neighbours (KNN) and Support-Vector Machine (SVM). Typically, this table is generated as the output of a standard machine learning pipeline (Figure 3.A - 3.C). In principle, MLcps can be calculated for any evaluation metric. However, it is highly recommended that all of them are on the same scale; for example, if accuracy ranges between 0 to 1, then the F1 metric should also be in the same range, not in percentage.

To calculate MLcps, the first step involves plotting the pre-calculated performance metrics on a two-dimensional polar coordinate system (Figure 3.D). In this polar coordinate system, each metric is represented as a ray, and the length of the ray corresponds to the metric value. This representation allows the polar plane to be divided into multiple triangles, with the number of triangles being equal to the available evaluation metrics. The combined area of these individual triangles represents the total area of the polar plane and serves as the MLcps (Figure 3.E).

Finally, the MLcps can be visually represented using a bar chart, as shown in Figure 3.F. It provides a clear and visually informative depiction of the relative performance of different machine learning methods. By examining the bar chart, one can easily identify the performance differences between various ML methods.

Area calculation of a two-dimensional polar plane

The projection of multiple evaluation metrics onto a two-dimensional polar coordinate system divides the polar plane into several triangles. Therefore, the total sum of the areas of these triangles is equal to the total area of the polar plane generated by the multiple performance scores. In order to calculate the area of each individual triangle, as described in Equation 1, we need to multiply half the length of base by the height drawn to that side (Figure 3.G-N).

Equation 1: $$\text{Area}_{\Delta ABC} = \frac{1}{2}\, ah$$

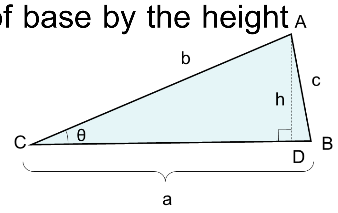

Where:

a = represents the side (base).

h = represents the height drawn to that side.

However, to apply this formula, we require the value for the height (h) variable, which cannot be controlled in a polar plane. Nonetheless, we do have control over the angles (θ) of all the triangles, which can be calculated by dividing 360 degrees by the number of performance metrics used, as described in Equation 2.

$$\text{Equation 2: Angle } \theta = \frac{360}{\text{Number of performance metrics}} \times \frac{\pi}{180} = \frac{2\pi}{\text{Number of performance metrics}}$$

Now, by employing trigonometry, as outlined in Equation 3, we can calculate the height (h) based on the known angles (θ). Therefore, the height of the triangle can be expressed as $h = b\ sin\ \theta$.

$$\text{Equation 3: } \sin\theta = \frac{\mathrm{h}}{b}$$

By substituting the new expression for the height (h) variable into the general formula for the area of a triangle, we obtain a new formula, as shown in Equation 4, where values for all the required variables are available.

$$\text{Equation 4: } \mathrm{Area}_{\Delta ABC} = \frac{1}{2}\, ab\mathrm{sin}\theta \text{ or } 2\mathrm{Area}_{\Delta ABC} = absin\theta$$

In Equation 4, the parameters a and b represent any two sides of a triangle, while θ denotes the included angle. It is important to note that in this context, the values a and b correspond to the actual measurements for each performance metric.

Finally, by utilizing Equation 5, derived from Equation 4, the total area of the polar plane can be determined by summing the areas of all triangles formed within the polar coordinate system.

$$\text{Equation 5: } 2\mathrm{Area}_{\mathrm{total}} = \sin\theta \sum_{i=1}^{n} d_i d_{i+1} \rightarrow \mathrm{Area}_{\mathrm{total}} = \frac{1}{2}\sin\theta \sum_{i=1}^{n} d_i d_{i+1}$$

Where:

d_i = length of the ith ray (the value of ith metric score) (Figure 3.L)

n = number of triangles point of collapse (Figure 3.M)

Weighted MLcps

In specific situations, certain metrics hold more significance than others. For instance, when dealing with an imbalanced dataset, achieving a high F1 score may be prioritized over higher accuracy[19,20]. In such cases, users have the option to assign weight variables to the metrics of interest during the calculation of MLcps. A weight variable assigns a value (referred to as the weight) to each pre-computed metric, and the respective metric scores are adjusted using these weights in the following manner:

$$\text{Equation 6: } \mathrm{S}_{\mathrm{weightedmetric}} = \mathrm{S}_{\mathrm{metric}} \times \mathrm{W}_{\mathrm{metric}}$$

Where:

$\mathrm{S}_{\mathrm{weightedmetric}}$ = Weighted metric Score

$\mathrm{S}_{\mathrm{metric}}$ = Raw metric Score

$\mathrm{W}_{\mathrm{metric}}$ = Weight

It is essential to note that the assigned weight for a metric must always be greater than or equal to zero. A weight of zero indicates that the user intends to exclude that metric from the MLcps calculation. Metrics with higher weights have a more significant contribution to the MLcps compared to metrics with lower weights. In the case where no weights are assigned (unweighted MLcps), it is equivalent to conducting a weighted analysis where all weights are set to 1.

Datasets

In this study, four distinct datasets were employed to evaluate MLcps (Table 1). The initial dataset comprises mRNA data (n=136) derived from a CLL study, which examined transcriptome profiles in individuals affected by blood cancer[21]. Our objective was to develop a model capable of distinguishing between male and female patients using their transcriptomic profiles. To achieve this, we focused on the top 5,000 most variably expressed mRNAs, excluding genes from the Y chromosome.

The second set of data was obtained from a study on cervical cancer, where the expression levels of 714 miRNAs were measured in human samples (n=58)[22]. The third and fourth datasets were collected from TCGA and involved mRNA (n=1219) and miRNA (n=1207) sequencing of BRCA. The TCGAbiolinks package in R was used to retrieve these datasets[23]. For the BRCA mRNA dataset, we focused on genes that showed differential expression according to edgeR analysis (FDR <= 0.001 and logFC > ±2)[24]. Our objective was to develop a model capable of distinguishing between normal and tumor samples for both the cervical cancer and TCGA-BRCA datasets.

The fifth dataset in our study comprises body signal data collected from 100,000 individuals through the National Health Insurance Service in Korea[25]. This dataset includes 21 essential biological signals related to health, such as measurements of systolic blood pressure and total cholesterol levels. Our main goal with this dataset was to determine whether individuals consume alcohol based on the available biological signal information.

Among these datasets, two were relatively small (CLL and the cervical cancer study), while the other two (TCGA datasets) were imbalanced (Table 1). We utilized an in-house ML pipeline (Figure S5) to train and evaluate eight different models (Table S1) to identify the best-performing model for CLL, cervical cancer, and the TCGA datasets. For the biological signal dataset, we utilized the 'customML' feature from the Machine Learning Made Easy (MLme)[26] tool to train and evaluate six different models and identify the best-performing one for classifying alcohol consumers and non-consumers.

Implementation

MLcps is developed using Python[27] and R[28] programming languages. Pandas[29,30] is used to store and process the data. Plotly[31] is used to generate the figures. The radarchart[32] package in R was used for surface area calculation of the polar plane. The R packages tibble[33] and dplyr[34] were utilized for data wrangling in the computation of MLcps during the analysis.

Table 1: Example datasets used in this study.

Dataset	Data type	Number of Samples	Number of Features	Target Class ratio
CLL	mRNA	136	5000	Male (n=82): Female (n=54)
Cervical cancer	miRNA	58	714	Normal (n=29): Tumor (n=29)
TCGA-BRCA	miRNA	1207	1404	Normal (n=104): Tumor (n=1104)
TCGA-BRCA	mRNA	1219	5520	Normal (n=113): Tumor (n=1106)
Body signal	Body signal data (hemoglobin, triglyceride)	100,000	21	**Consume Alcohol** Yes (n=50173): No (n= 49827)

Conclusion

Our paper introduces MLcps, a novel evaluation metric implemented as a Python package. MLcps is a robust evaluation metric designed specifically for classification problems. Its ability to integrate multiple evaluation metrics into a single score makes it an efficient and reliable approach for evaluating model performance and selecting the most successful model. This is especially valuable when multiple evaluation metrics are necessary to fully comprehend a model's strengths and weaknesses.

However, it's essential to understand that the reliability of MLcps depends on the quality of the metrics used in its calculation. Therefore, it is of utmost importance to employ appropriate evaluation metrics, which depend on various factors such as the specific domain, stakeholder preferences, and data characteristics. Similarly, assigning weights to evaluation metrics in machine learning offers a valuable technique for prioritizing specific aspects of model performance, but it comes with potential drawbacks and complexities. For example, heavily weighting one metric can overshadow the overall evaluation, possibly resulting in suboptimal models. Additionally, the assignment of metric weights often depends on subjective judgments regarding their relative significance. Various stakeholders may hold differing perspectives on how much weight to allocate to each metric, potentially leading to evaluation bias.

While the allocation of weights to evaluation metrics can enhance the customization of the evaluation process for specific objectives, it must be executed judiciously,

considering the possible downsides and challenges associated with this approach. Striking a balance between highlighting key metrics and maintaining a comprehensive view of model performance is paramount. Therefore, we strongly discourage relying on MLcps without considering the context in which it is applied.

Availability of supporting source code and requirements

Project name: Machine Learning cumulative performance score (MLcps)

Project home page: https://github.com/FunctionalUrology/MLcps

Operating system(s): Platform independent

Programming language: Python >=3.8 and R >=4.0

Other requirements: radarchart, tibble, and dplyr R packages.

License: GNU GPL

Authors' Contributions

K.M., A.H.G, and A.A. conceived the idea for the manuscript. A.A. and M.K. wrote the source code in addition to carrying out testing and debugging of the MLcps. K.M., F.C.B, and A.H.G tested the MLcps and provided scientific inputs throughout the development phase. F.C.B, R.M.A and A.B.A provided the feedback on biological application of the tool. N.S and M.A provided the mathematical support and did the testing and debugging. All authors contributed to writing, proofreading, and correcting the manuscript.

Funding

We gratefully acknowledge the financial support of the Swiss National Science Foundation (SNF Grant 310030_175773 to FCB and KM, 212298 to FCB and AHG) and the Wings for Life Spinal Cord Research Foundation (WFL-AT-06/19 to KM). AHG and RMA are supported by R01 DK 077195 and R01 DK127673. MK is supported by the Else Kröner-Fresenius-Stiftung (EKFS 2021_EKeA.33). The authors acknowledge the financial support from the Federal Ministry of Education and Research of Germany and by the Sächsische Staatsministerium für Wissenschaft Kultur und Tourismus in the program Center of Excellence for AI-research "Center for Scalable Data Analytics and Artificial Intelligence Dresden/Leipzig" (project identification number: ScaDS.AI).

Competing Interests

The authors have declared no competing interests.

Data availability

Not applicable. DOME-ML (Data, Optimisation, Model, and Evaluation in Machine Learning) annotation, supporting the current study, is available through DOME Wizard.

Acknowledgment

We express our sincere gratitude to Dr. Nezhla Aghaei for their invaluable inspiration, assistance in guiding us through the mathematical formulation and providing expert consultation in the calculation of the planar surface area.

References

1. Sun, Y., Wong, A. K. C. & Kamel, M. S. Classification of imbalanced data: a review. *Int. J. Patt. Recogn. Artif. Intell.* **23**, 687–719 (2009).
2. Russo, D. P., Zorn, K. M., Clark, A. M., Zhu, H. & Ekins, S. Comparing Multiple Machine Learning Algorithms and Metrics for Estrogen Receptor Binding Prediction. *Mol. Pharmaceutics* **15**, 4361–4370 (2018).
3. Stevens, L. M., Mortazavi, B. J., Deo, R. C., Curtis, L. & Kao, D. P. Recommendations for Reporting Machine Learning Analyses in Clinical Research. *Circulation: Cardiovascular Quality and Outcomes* **13**, e006556 (2020).
4. Biswas, A., Saran, I. & Wilson, F. P. Introduction to Supervised Machine Learning. *Kidney360* **2**, 878 (2021).
5. Rashidi, H. H., Albahra, S., Robertson, S., Tran, N. K. & Hu, B. Common statistical concepts in the supervised Machine Learning arena. *Front Oncol* **13**, 1130229 (2023).
6. Hicks, S. A. *et al.* On evaluation metrics for medical applications of artificial intelligence. *Sci Rep* **12**, 5979 (2022).
7. Ahmadzadeh, A., Kempton, D. J., Martens, P. C. & Angryk, R. A. Contingency Space: A Semimetric Space for Classification Evaluation. *IEEE Transactions on Pattern Analysis and Machine Intelligence* **45**, 1501–1513 (2023).
8. Huang, J., Lu, J. & Ling, C. X. Comparing naive Bayes, decision trees, and SVM with AUC and accuracy. in *Third IEEE International Conference on Data Mining* 553–556 (2003). doi:10.1109/ICDM.2003.1250975.
9. Provost, F. & Domingos, P. Tree Induction for Probability-Based Ranking. *Machine Learning* **52**, 199–215 (2003).
10. Huang, J. & Ling, C. X. Using AUC and accuracy in evaluating learning algorithms. *IEEE Transactions on Knowledge and Data Engineering* **17**, 299–310 (2005).
11. Jeni, L. A., Cohn, J. F. & De La Torre, F. Facing Imbalanced Data–Recommendations for the Use of Performance Metrics. in *2013 Humaine Association Conference on Affective Computing and Intelligent Interaction* 245–251 (2013). doi:10.1109/ACII.2013.47.
12. Stafford, I. S. *et al.* A systematic review of the applications of artificial intelligence and machine learning in autoimmune diseases. *NPJ Digit Med* **3**, 30 (2020).
13. Zhou, J., Gandomi, A. H., Chen, F. & Holzinger, A. Evaluating the Quality of Machine Learning Explanations: A Survey on Methods and Metrics. *Electronics* **10**, 593 (2021).
14. Adhikari, S., Normand, S.-L., Bloom, J., Shahian, D. & Rose, S. Revisiting performance metrics for prediction with rare outcomes. *Stat Methods Med Res* **30**, 2352–2366 (2021).
15. Rácz, A., Bajusz, D. & Héberger, K. Multi-Level Comparison of Machine Learning Classifiers and Their Performance Metrics. *Molecules* **24**, 2811 (2019).
16. Branco, P., Torgo, L. & Ribeiro, R. P. A Survey of Predictive Modeling on Imbalanced Domains. *ACM Comput. Surv.* **49**, 31:1-31:50 (2016).
17. Chakrabarti, S., Khanna, R., Sawant, U. & Bhattacharyya, C. Structured learning for non-smooth ranking losses. in *Proceedings of the 14th ACM SIGKDD international conference on Knowledge discovery and data mining* 88–96 (Association for Computing Machinery, 2008). doi:10.1145/1401890.1401906.

18. Geng, X. & Cheng, X.-Q. Learning multiple metrics for ranking. *Front. Comput. Sci. China* **5**, 259–267 (2011).
19. Galar, M., Fernandez, A., Barrenechea, E., Bustince, H. & Herrera, F. A Review on Ensembles for the Class Imbalance Problem: Bagging-, Boosting-, and Hybrid-Based Approaches. *IEEE Transactions on Systems, Man, and Cybernetics, Part C (Applications and Reviews)* **42**, 463–484 (2012).
20. Uzun Ozsahin, D., Onakpojeruo, E. P., Uzun, B., Mustapha, M. T. & Ozsahin, I. Mathematical Assessment of Machine Learning Models Used for Brain Tumor Diagnosis. *Diagnostics (Basel)* **13**, 618 (2023).
21. Dietrich, S. *et al.* Drug-perturbation-based stratification of blood cancer. *J Clin Invest* **128**, 427–445 (2018).
22. Witten, D., Tibshirani, R., Gu, S. G., Fire, A. & Lui, W.-O. Ultra-high throughput sequencing-based small RNA discovery and discrete statistical biomarker analysis in a collection of cervical tumours and matched controls. *BMC Biology* **8**, 58 (2010).
23. Colaprico, A. *et al.* TCGAbiolinks: an R/Bioconductor package for integrative analysis of TCGA data. *Nucleic Acids Research* **44**, e71 (2016).
24. Robinson, M. D., McCarthy, D. J. & Smyth, G. K. edgeR: a Bioconductor package for differential expression analysis of digital gene expression data. *Bioinformatics* **26**, 139–140 (2010).
25. Her, S. Smoking and Drinking Dataset with body signal. *Kaggle* https://www.kaggle.com/datasets/sooyoungher/smoking-drinking-dataset.
26. Akshay, A. *et al.* Machine Learning Made Easy (MLme): A Comprehensive Toolkit for Machine Learning-Driven Data Analysis. 2023.07.04.546825 Preprint at https://doi.org/10.1101/2023.07.04.546825 (2023).
27. van Rossum, G. Python reference manual. (1995).
28. R Core Team. *R: A Language and Environment for Statistical Computing*. (R Foundation for Statistical Computing, 2022).
29. McKinney, W. Data Structures for Statistical Computing in Python. *Proceedings of the 9th Python in Science Conference* 56–61 (2010) doi:10.25080/Majora-92bf1922-00a.
30. The pandas development team. pandas-dev/pandas: Pandas. (2020) doi:10.5281/zenodo.3509134.
31. Inc, P. T. Collaborative data science. https://plot.ly (2015).
32. Porter, D. A. S. radarchart: Radar Chart from ‘Chart.js’. *R Package* **0.3.1**, (2016).
33. Müller, K. & Wickham, H. *tibble: Simple Data Frames*. (2023).
34. Wickham, H., François, R., Henry, L., Müller, K. & Vaughan, D. *dplyr: A Grammar of Data Manipulation*. (2023).

Figure-Revised
Click here to access/download;Figure;figures.pdf

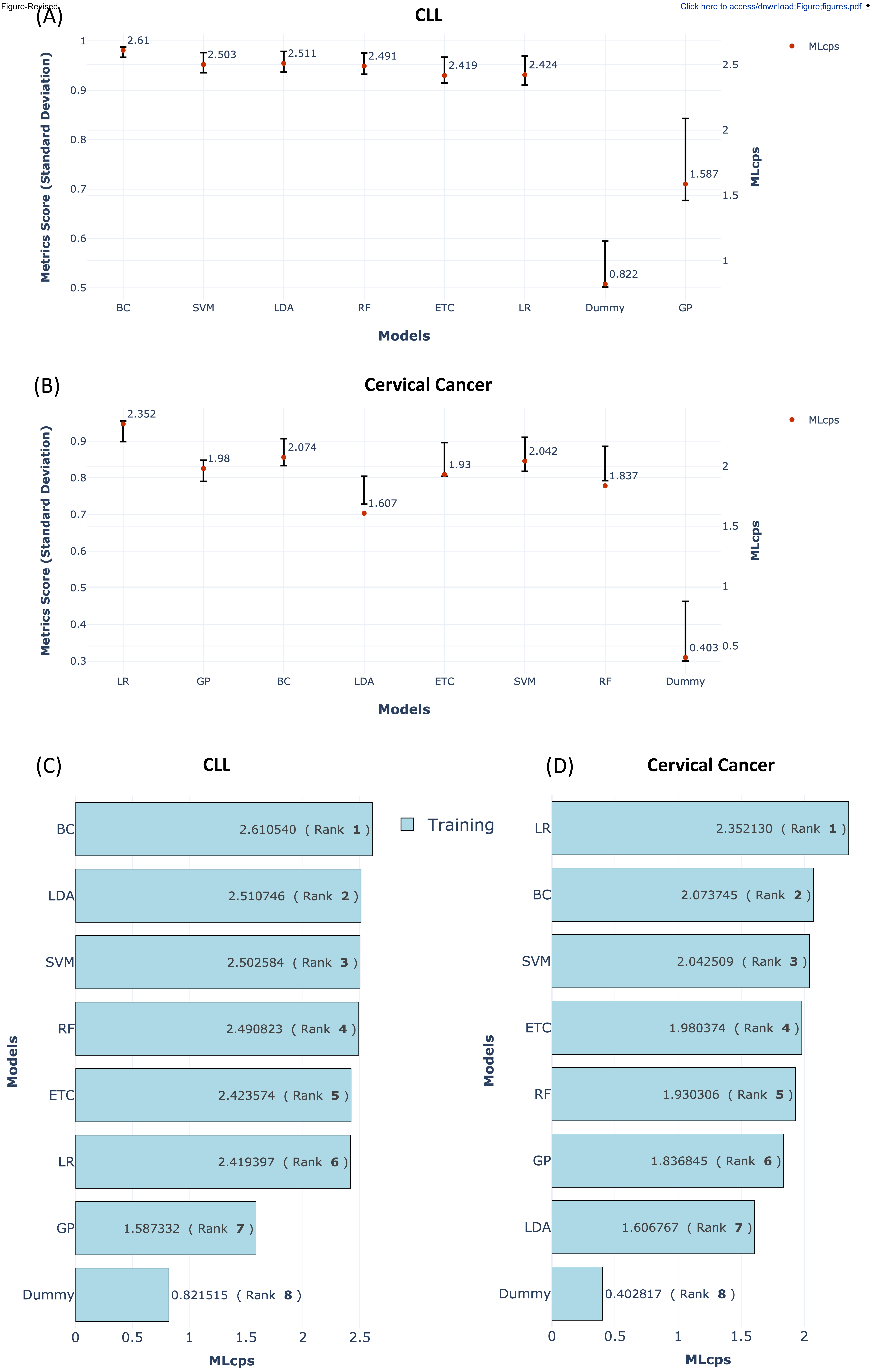

Figure 1. Standard deviation (SD) of Performance Metrics and MLcps Comparison for CLL and Cervical Cancer Datasets. Figure (A) and (B) illustrate the SD of performance metric scores for ML algorithms trained on the CLL and Cervical Cancer datasets, respectively. The bars in the plot represent the SD of performance metric scores and are displayed on the left y-axis. The bars are arranged from left to right, with smaller SD values on the left and larger SD values on the right. A red dot on the plot represents the MLcps which is displayed on the right y-axis. Figure (C) and (D), represent MLcps for training data from the CLL and Cervical Cancer datasets, respectively. The numerical MLcps values are indicated within each bar. Rankings, enclosed in brackets, reflect model performance based on MLcps.

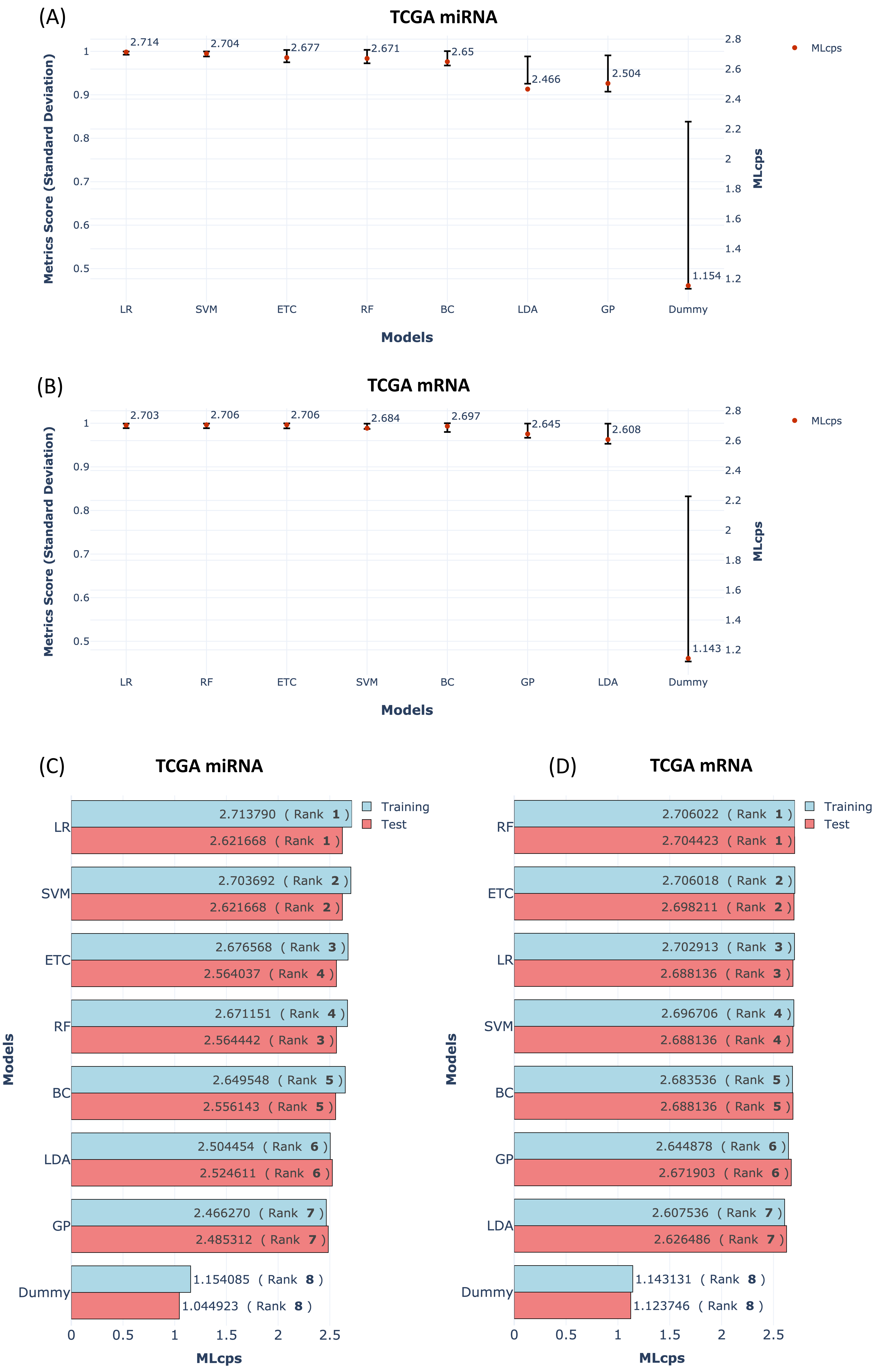

Figure 2. Standard deviation (SD) of Performance Metrics and MLcps Comparison for TCGA mRNA and miRNA Datasets. Figure (A) and (B) illustrate the SD of performance metric scores for ML algorithms trained on the mRNA and miRNA datasets, respectively. The bars in the plot represent the SD of performance metric scores and are displayed on the left y-axis. The bars are arranged from left to right, with smaller SD values on the left and larger SD values on the right. A red dot on the plot represents the MLcps which is displayed on the right y-axis. Figure (C) and (D), represent a comparison of MLcps for training and test data from the mRNA and miRNA datasets, respectively. The numerical MLcps values are indicated within each bar. Rankings, enclosed in brackets, reflect model performance based on MLcps, whether computed from the training or test data.

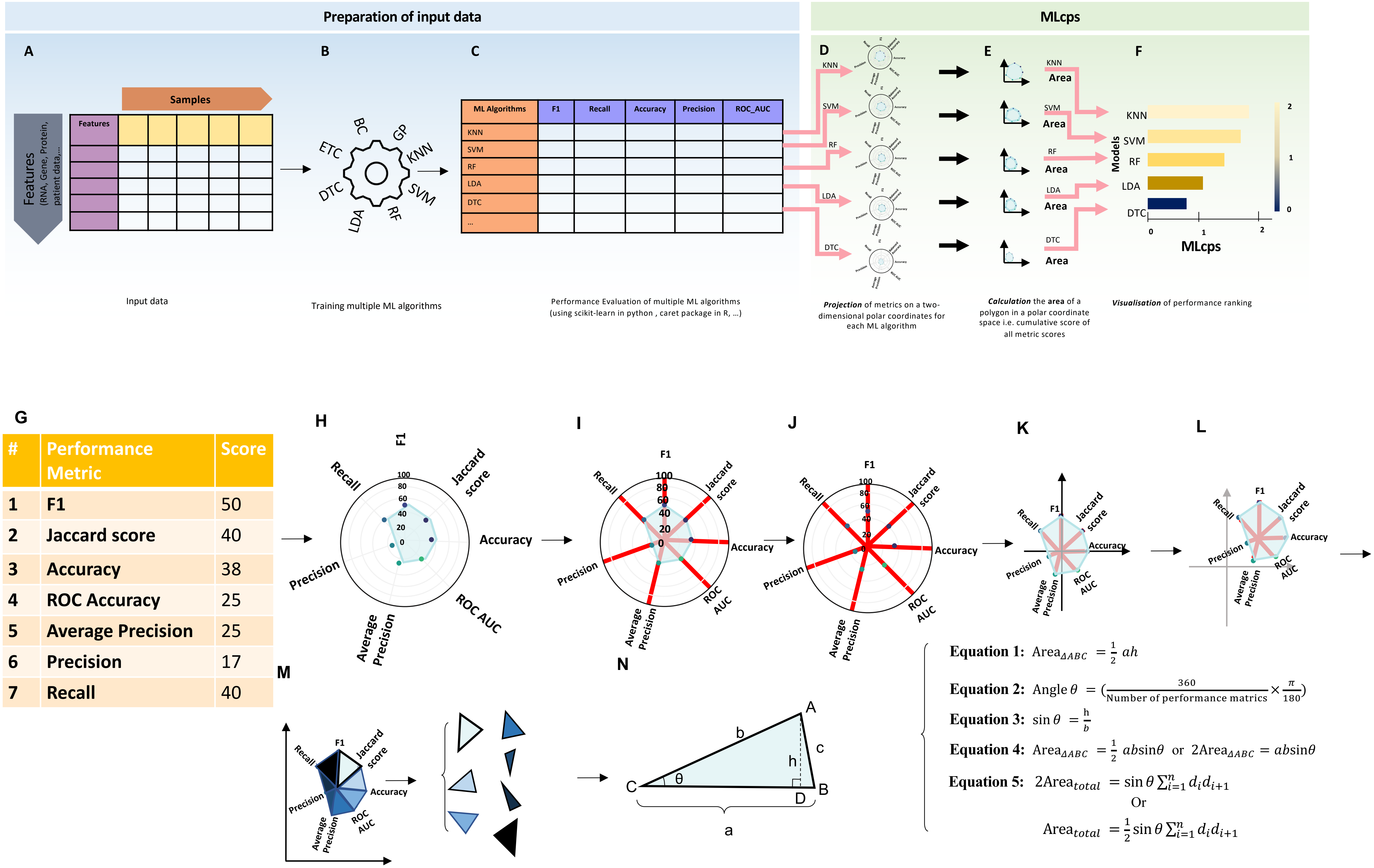

Figure 3 Schematic overview of the complete analysis process for MLcps Python package. Before using the MLcps Python package, one needs to prepare the raw data (A). This input table can be RNA sequencing, proteomics, patients' profile, molecular data, etc (normally this data is in txt or csv format). Next step is to perform multiple ML algorithms (B). Performing this step can be done by any package or programming language of choice. The next step is to evaluate the performance of the ML algorithms. We recommend the use of multiple metrics such as F1, Recall, etc (C). The performance metric scores then need to be arranged in a tabular format as depicted in (C). This table will be used as an input for the MLcps package. From here on the MLcps will process the data. MLcps involves three steps: projection, calculation, and visualization (PCV). To calculate the cumulative score of each ML algorithm in the input data, MLcps first projects the performance metric onto the two-dimensional polar coordinates system (D). Next, the projected polygon's area is calculated (E). Finally, the user can visualize this MLcps to rank the performance of given ML algorithms (F). The lower panel (G-N) visualises the procedure to calculate the surface area as cumulative score in detail. The names of the algorithms are just mentioned as example and other algorithm can be used too. ETC: Extra Trees Classifier, BC: Bagging Classifier, GP: Gaussian Process Classifier, KNN: K-Nearest Neighbors, SVM: Support Vector Machine, RF: Random Forest Classifier, LDA: Linear Discriminant Analysis, DTC: Decision Tree Classifier.

Click here to access/download
Supplementary Material
suppl.pdf

Click here to access/download
Supplementary Material
figures_supp.pdf
