# Supplementary material for: MLcps: machine learning cumulative performance score for classification problems
Source: Gigascience. 2023 Dec 13;12:giad108. doi: 10.1093/gigascience/giad108 (PMC10716825; doi:10.1093/gigascience/giad108)
Supplement: giad108_GIGA-D-23-00187_Revision_2 [file giad108_giga-d-23-00187_revision_2.pdf]

GigaScience

MLcps: Machine Learning Cumulative Performance Score for Classification Problems

--Manuscript Draft--

**Manuscript Number:**	GIGA-D-23-00187R2
**Full Title:**	MLcps: Machine Learning Cumulative Performance Score for Classification Problems
**Article Type:**	Technical Note
**Funding Information:**	Schweizerischer Nationalfonds zur Förderung der Wissenschaftlichen Forschung (310030_175773) — Prof Fiona C. Burkhard<br>Schweizerischer Nationalfonds zur Förderung der Wissenschaftlichen Forschung (212298) — Prof Fiona C. Burkhard<br>Wings for Life (WFL-AT-06/19) — Prof Katia Monastyrskaya<br>Else Kröner-Fresenius-Stiftung (EKFS 2021_EKeA.33) — Not applicable<br>Sächsisches Staatsministerium für Wissenschaft und Kunst (ScaDS.AI) — Not applicable
**Abstract:**	Background<br>Assessing the performance of machine learning (ML) models requires careful consideration of the evaluation metrics used. It is often necessary to utilize multiple metrics to gain a comprehensive understanding of a trained model's performance, as each metric focuses on a specific aspect. However, comparing the scores of these individual metrics for each model to determine the best-performing model can be time-consuming and susceptible to subjective user preferences, potentially introducing bias.<br>Results<br>We propose Machine Learning Cumulative Performance Score (MLcps), a novel evaluation metric for classification problems. MLcps integrates several pre-computed evaluation metrics into a unified score, enabling a comprehensive assessment of the trained model's strengths and weaknesses. We tested MLcps on four publicly available datasets, and the results demonstrate that MLcps provides a holistic evaluation of the model's robustness, ensuring a thorough understanding of its overall performance.<br>Conclusion<br>By utilizing MLcps, researchers and practitioners no longer need to individually examine and compare multiple metrics to identify the best-performing models. Instead, they can rely on a single MLcps value to assess the overall performance of their ML models. This streamlined evaluation process saves valuable time and effort, enhancing the efficiency of model evaluation. MLcps is available as a python package at https://pypi.org/project/MLcps/ and examples of its use can be found at https://mybinder.org/v2/gh/FunctionalUrology/MLcps.git/main.
**Corresponding Author:**	Ali Hashemi Gheinani<br>Harvard Medical School<br>SWITZERLAND
**Corresponding Author Secondary Information:**	
**Corresponding Author's Institution:**	Harvard Medical School
**Corresponding Author's Secondary Institution:**	
**First Author:**	Akshay Akshay
**First Author Secondary Information:**	
**Order of Authors:**	Akshay Akshay<br>Masoud Abedi

Powered by Editorial Manager® and ProduXion Manager® from Aries Systems Corporation

	Navid Shekarchizadeh
	Fiona C. Burkhard
	Mitali Katoch
	Alex Bigger-Allen
	Rosalyn M. Adam
	Katia Monastyrskaya
	Ali Hashemi Gheinani
**Order of Authors Secondary Information:**	
**Response to Reviewers:**	As per request we added a section titled "Data Availability" in addition to the "code availability" section.<br>We have also included software application in the bio.tools and SciCrunch.org databases tand biotoolsID identifiers, and include these in your manuscript. We added the RRID and biotool IDs in the "code availability" section.<br>Thanks
**Additional Information:**	
**Question**	**Response**
Are you submitting this manuscript to a special series or article collection?	No
**Experimental design and statistics**<br><br>Full details of the experimental design and statistical methods used should be given in the Methods section, as detailed in our Minimum Standards Reporting Checklist. Information essential to interpreting the data presented should be made available in the figure legends.<br><br>Have you included all the information requested in your manuscript?	Yes
**Resources**<br><br>A description of all resources used, including antibodies, cell lines, animals and software tools, with enough information to allow them to be uniquely identified, should be included in the Methods section. Authors are strongly encouraged to cite Research Resource Identifiers (RRIDs) for antibodies, model organisms and tools, where possible.<br><br>Have you included the information requested as detailed in our Minimum	Yes

Standards Reporting Checklist?	
**Availability of data and materials**<br><br>All datasets and code on which the conclusions of the paper rely must be either included in your submission or deposited in publicly available repositories (where available and ethically appropriate), referencing such data using a unique identifier in the references and in the "Availability of Data and Materials" section of your manuscript.<br><br>Have you have met the above requirement as detailed in our Minimum Standards Reporting Checklist?	Yes

Powered by Editorial Manager® and ProduXion Manager® from Aries Systems Corporation

Manuscript

Click here to access/download;Manuscript;MLcps_for_production.docx

MLcps: Machine Learning Cumulative Performance Score for Classification Problems

Akshay Akshay (ORCID: 0000-0003-3186-7478) [1,2], Masoud Abedi (0000-0003-3986-4028)[3], Navid Shekarchizadeh (0000-0002-5750-7801)[3,4], Fiona C. Burkhard (0000-0002-8271-014X)[1,5], Mitali Katoch (0000-0002-9248-6348)[6], Alex Bigger-Allen (0000-0001-6914-9135) [7,8,9,10], Rosalyn M. Adam (0000-0002-0943-6236) [8,9,10], Katia Monastyrskaya (0000-0003-2042-1139) [1, 5] and Ali Hashemi Gheinani (0000-0002-9625-6259) [1,5,8,9,10]*

[1] Functional Urology Research Group, Department for BioMedical Research DBMR, University of Bern, Switzerland

[2] Graduate School for Cellular and Biomedical Sciences, University of Bern, Switzerland

[3]Department of Medical Data Science, Leipzig University Medical Centre, 04107 Leipzig, Germany

[4]Center for Scalable Data Analytics and Artificial Intelligence (ScaDS.AI) Dresden/Leipzig, 04105 Leipzig, Germany

[5] Department of Urology, Inselspital University Hospital, 3010 Bern, Switzerland

[6] Institute of Neuropathology, Universitätsklinikum Erlangen, Friedrich-Alexander-Universität Erlangen-Nürnberg (FAU), Erlangen, Germany

[7] Biological & Biomedical Sciences Program, Division of Medical Sciences, Harvard Medical School, Boston, MA.

[8] Urological Diseases Research Center, Boston Children's Hospital, MA, USA

[9] Harvard Medical School, Boston, Department of Surgery MA, USA

[10] Broad Institute of MIT and Harvard, Cambridge, MA, USA

* Corresponding author:

Ali Hashemi Gheinani, Urological Diseases Research Center, Boston Children's Hospital, Harvard Medical School and Broad Institute of MIT and Harvard, Cambridge, MA, USA

e-mail: Ali.HashemiGheinani@childrens.harvard.edu

Keywords

- Machine Learning
- Classification Problems
- Model Evaluation
- Unified Evaluation Score
- Python Package

Key Points

- Evaluating machine learning models involves considering multiple metrics. Comparing scores of individual metrics to determine the best model can be time-consuming and subjective, potentially introducing bias.

- The proposed Machine Learning Cumulative Performance Score (MLcps) is a novel evaluation metric for classification problems. It integrates multiple evaluation metrics into a unified score, providing a holistic understanding of model performance.

- MLcps outperforms standard metric-based rankings, offering a more reliable and consistent assessment of model performance.

- MLcps is available as a Python package, making it easily accessible for researchers to incorporate into their evaluation pipelines.

Abstract

Background

Assessing the performance of machine learning (ML) models requires careful consideration of the evaluation metrics used. It is often necessary to utilize multiple metrics to gain a comprehensive understanding of a trained model's performance, as each metric focuses on a specific aspect. However, comparing the scores of these individual metrics for each model to determine the best-performing model can be time-consuming and susceptible to subjective user preferences, potentially introducing bias.

Results

We propose Machine Learning Cumulative Performance Score (MLcps), a novel evaluation metric for classification problems. MLcps integrates several pre-computed evaluation metrics into a unified score, enabling a comprehensive assessment of the trained model's strengths and weaknesses. We tested MLcps on four publicly available datasets, and the results demonstrate that MLcps provides a holistic evaluation of the model's robustness, ensuring a thorough understanding of its overall performance.

Conclusion

By utilizing MLcps, researchers and practitioners no longer need to individually examine and compare multiple metrics to identify the best-performing models. Instead, they can rely on a single MLcps value to assess the overall performance of their ML models. This streamlined evaluation process saves valuable time and effort, enhancing the efficiency of model evaluation. MLcps is available as a python package at https://pypi.org/project/MLcps/ and examples of its use can be found at https://mybinder.org/v2/gh/FunctionalUrology/MLcps.git/main.

Introduction

The evaluation of machine learning (ML) models is crucial in the ML workflow as it helps determine their effectiveness. However, it is essential to select the appropriate evaluation metric since the performance of a trained model is only as good as the metric used for evaluation[1–5]. Numerous metrics are available for assessing the performance of ML models, with each metric focusing on a specific aspect of the model's performance[6,7]. For example, the "recall" metric effectively measures a model's ability to predict positive class instances but does not provide insights into the negative class instances. This poses a significant challenge because a model that performs well according to one metric may not exhibit the same level of performance when evaluated using another metric[8–14]. Hence, relying solely on a single performance metric is inadequate in practical scenarios.

Furthermore, the characteristics and composition of the available dataset can influence the behaviour and outcomes of various metrics. For instance, when dealing with imbalanced datasets, accuracy becomes an inadequate metric, and relying solely on accuracy can lead to misleading interpretations[15]. Therefore, it is crucial to calculate multiple performance metrics for each model to evaluate its performance comprehensively[7]. By considering various evaluation metrics, we can gain a holistic view of a model's performance and make informed decisions about the best-performing model for a given task.

When calculating multiple metrics for a model, there is often an assumption that the best model will consistently achieve the highest scores across all metrics. However, this assumption is rarely true in practical scenarios, necessitating the comparison of the individual metrics of different models to identify the best-performing model. However, comparing metric scores for many models can be labour-intensive and susceptible to user preference bias[16]. As a result, the complexity of finding the best model increases exponentially when considering the comparison of different metrics.

Apart from these limitations, some methods prevent users from evaluating model performance with multiple metrics simultaneously. For example, in the field of biology, the wrapper-based feature selection method is commonly used to identify important features from a large set of original attributes. This method trains a model with different feature subsets and selects the subset that shows the best performance compared to the other subsets. Unfortunately, these methods are limited to evaluating model performance using only one metric at a time. This constraint can potentially lead to overfitting to a specific metric, resulting in the selection of suboptimal feature subsets that lack generalizability.

In the realm of Information Retrieval (IR), Chakrabarti et al. previously introduced novel algorithms designed to merge multiple ranking criteria into a unified approach, ultimately enhancing the optimization of search results[17]. Building upon this research, Geng et al. further investigated learning to rank, considering multiple evaluation metrics, and proposed the combination of multiple metrics to optimize IR metrics[18].

Here, we introduce a novel evaluation metric called the Machine Learning Cumulative Performance Score (MLcps) to address the challenges associated with model evaluation in the

field of machine learning. MLcps is a unified score that follows a similar methodology compared to the previously mentioned study related to IR. MLcps combines precomputed performance metrics into a single score while preserving their distinct characteristics. By leveraging multiple metrics, MLcps provides a more comprehensive evaluation of machine learning model performance. To enhance the accessibility of MLcps, we have implemented it as a Python package, enabling direct comparisons of trained ML models to assess their performance.

Results and Discussion

In this section, the results of the current study are showcased, with a specific focus on evaluating MLcps as a robust measure for assessing ML model performance. The primary objective of this analysis is to shed light on the effectiveness of MLcps in ranking models based on their consistency and excellence across multiple performance metrics. Furthermore, we explore the reliability of MLcps in selecting models that not only excel on training data but also demonstrate the ability to generalize well to unseen datasets.

Additionally, we emphasize the importance of employing a diverse set of performance metrics when evaluating machine learning models. By doing so, we aim to provide a comprehensive understanding of model performance beyond traditional measures and showcase the significance of considering various aspects of model behaviour in real-world applications.

Evaluating MLcps Robustness

Each performance metric represents a specific aspect of model performance, and for a model to be considered robust and superior, it should consistently excel across all these metrics. This consistency can be reflected by having the lowest standard deviation (SD) across performance metrics. Therefore, our analysis revolves around understanding the relationship between MLcps and SD. This evaluation helps determine the reliability of MLcps as a performance measure.

To assess MLcps' robustness as a model performance measure, we analyzed multiple models across five distinct datasets (Table 1). Our findings consistently revealed a strong correlation between the highest MLcps score and the lowest standard deviation (SD) in performance metric scores (Figures 1A-B and 2A-B). This correlation indicates that MLcps reliably identifies the best-performing model when it consistently excels across all metrics, validating its reliability as a performance measure.

However, there are important exceptions that require attention. For instance, in the Chronic Lymphocytic Leukemia (CLL) dataset, the GP model outperforms the Dummy model in terms of MLcps score, even though the Dummy model has a lower SD (Figure 1A). Similarly, in the cervical cancer dataset, the MLcps scores of the ETC, SVM, and RF models surpass that of the LDA model, despite the LDA model having a lower SD (Figure 1B). Similar exceptions were observed in the body signals dataset as well (Figure S4.A).

These exceptions can be attributed to the fact that while these models exhibit lower SD compared to others, they also perform poorly for each individual metric.

Consequently, their low MLcps scores accurately reflect their subpar performance across all metrics. This observation acknowledges that a model with poor performance metrics may still have a smaller SD when compared to other models. These exceptions underscore that MLcps takes into account not only the SD but also the overall magnitude of performance metric scores, thereby providing a comprehensive evaluation of ML models' performance.

Consistency in Model Performance across Training and Test Datasets

To evaluate the reliability of MLcps in selecting the best-performing models, we examined the consistency of model performance between the training and test datasets. Among the five datasets, the The Cancer Genome Atlas (TCGA) - Breast Invasive Carcinoma (BRCA) and Body Signals datasets offered a larger sample size, allowing us to create an independent test set comprising 30% of the data. When analyzing these three datasets, we found that the model identified as the best performer based on MLcps also demonstrated the best performance on the independent test set (Figures 2C-D).

Furthermore, it is noteworthy that if we solely relied on the SD to rank the models, the LR model would have been chosen as the best performer on the training dataset of TCGA-BRCA mRNA (Figure 2B). However, when evaluating its performance on the test dataset, LR did not even rank among the top two (Figure 2D). Similarly, in the Body Signal dataset, the Bagging Classifier model would have been considered the best performer based on the SD criteria (Figure S4.A). However, it's important to note that on the test dataset, this model ranked fourth in terms of performance (Figure S4.B).

In contrast, when sorting the model performance based on MLcps, the ranking remained consistent across both training and test datasets, providing a more robust measure of model performance (Figure S4.B). These findings indicate that MLcps effectively identifies models that not only perform well on the training data but also generalize well to unseen data, highlighting its comprehensive ability to assess model performance across different datasets.

Importance of Utilizing Multiple Performance Metrics

To emphasize the significance of using multiple performance metrics in evaluating ML model performance, we employed a visual representation of the metric scores using a two-dimensional polar coordinate system for each ML algorithm trained on different datasets. Our results demonstrated that both precision and average precision metrics consistently yielded high scores (>90%) for all the trained models in the TCGA miRNA (Figures S1 B-C) and mRNA datasets (Figure S2 B-C). However, relying solely on these metrics would have resulted in mistakenly selecting the dummy model as the best-performing one. This highlights the crucial importance of incorporating multiple performance metrics to obtain a more accurate assessment of ML model performance. Importantly, this phenomenon was not observed in the CLL and cervical cancer datasets (Figure S1.A, Figure S2.A), indicating that the interpretation of performance metrics is dataset dependent. By considering a diverse range of metrics, researchers and practitioners can make more informed decisions regarding the usefulness and reliability of ML models.

Material and Methods

MLcps Methodology

The MLcps algorithm requires an input table consisting of columns that hold various performance metrics, such as F1, Accuracy, and Recall. The rows in the table represent different machine learning methods, such as K-Nearest Neighbours (KNN) and Support-Vector Machine (SVM). Typically, this table is generated as the output of a standard machine learning pipeline (Figure 3.A - 3.C). In principle, MLcps can be calculated for any evaluation metric. However, it is highly recommended that all of them are on the same scale; for example, if accuracy ranges between 0 to 1, then the F1 metric should also be in the same range, not in percentage.

To calculate MLcps, the first step involves plotting the pre-calculated performance metrics on a two-dimensional polar coordinate system (Figure 3.D). In this polar coordinate system, each metric is represented as a ray, and the length of the ray corresponds to the metric value. This representation allows the polar plane to be divided into multiple triangles, with the number of triangles being equal to the available evaluation metrics. The combined area of these individual triangles represents the total area of the polar plane and serves as the MLcps (Figure 3.E).

Finally, the MLcps can be visually represented using a bar chart, as shown in Figure 3.F. It provides a clear and visually informative depiction of the relative performance of different machine learning methods. By examining the bar chart, one can easily identify the performance differences between various ML methods.

Area calculation of a two-dimensional polar plane

The projection of multiple evaluation metrics onto a two-dimensional polar coordinate system divides the polar plane into several triangles. Therefore, the total sum of the areas of these triangles is equal to the total area of the polar plane generated by the multiple performance scores. In order to calculate the area of each individual triangle, as described in Equation 1, we need to multiply half the length of base by the height drawn to that side (Figure 3.G-N).

Equation 1: $\text{Area}_{\Delta ABC} = \frac{1}{2}\, ah$

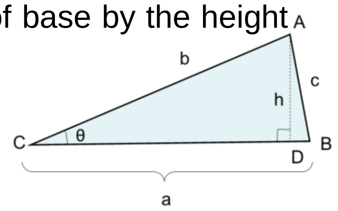

Where:

a = represents the side (base).

h = represents the height drawn to that side.

However, to apply this formula, we require the value for the height (h) variable, which cannot be controlled in a polar plane. Nonetheless, we do have control over the angles (θ) of all the triangles, which can be calculated by dividing 360 degrees by the number of performance metrics used, as described in Equation 2.

$$\text{Equation 2: Angle } \theta = \frac{360}{\text{Number of performance metrics}} \times \frac{\pi}{180} = \frac{2\pi}{\text{Number of performance metrics}}$$

Now, by employing trigonometry, as outlined in Equation 3, we can calculate the height (h) based on the known angles (θ). Therefore, the height of the triangle can be expressed as $h = b \sin\theta$.

$$\text{Equation 3: } \sin\theta = \frac{\mathrm{h}}{b}$$

By substituting the new expression for the height (h) variable into the general formula for the area of a triangle, we obtain a new formula, as shown in Equation 4, where values for all the required variables are available.

$$\text{Equation 4: } \mathrm{Area}_{\Delta ABC} = \frac{1}{2} ab\sin\theta \text{ or } 2\mathrm{Area}_{\Delta ABC} = absin\theta$$

In Equation 4, the parameters a and b represent any two sides of a triangle, while θ denotes the included angle. It is important to note that in this context, the values a and b correspond to the actual measurements for each performance metric.

Finally, by utilizing Equation 5, derived from Equation 4, the total area of the polar plane can be determined by summing the areas of all triangles formed within the polar coordinate system.

$$\text{Equation 5: } 2\mathrm{Area}_{\mathrm{total}} = \sin\theta \sum_{i=1}^{n} d_i d_{i+1} \rightarrow \mathrm{Area}_{\mathrm{total}} = \frac{1}{2}\sin\theta \sum_{i=1}^{n} d_i d_{i+1}$$

Where:

d_i = length of the ith ray (the value of ith metric score) (Figure 3.L)

n = number of triangles point of collapse (Figure 3.M)

Weighted MLcps

In specific situations, certain metrics hold more significance than others. For instance, when dealing with an imbalanced dataset, achieving a high F1 score may be prioritized over higher accuracy[19,20]. In such cases, users have the option to assign weight variables to the metrics of interest during the calculation of MLcps. A weight variable assigns a value (referred to as the weight) to each pre-computed metric, and the respective metric scores are adjusted using these weights in the following manner:

$$\text{Equation 6: } \mathrm{S}_{\mathrm{weightedmetric}} = \mathrm{S}_{\mathrm{metric}} \times \mathrm{W}_{\mathrm{metric}}$$

Where:

$\mathrm{S}_{\mathrm{weightedmetric}}$ = Weighted metric Score

$\mathrm{S}_{\mathrm{metric}}$ = Raw metric Score

$\mathrm{W}_{\mathrm{metric}}$ = Weight

It is essential to note that the assigned weight for a metric must always be greater than or equal to zero. A weight of zero indicates that the user intends to exclude that metric from the MLcps calculation. Metrics with higher weights have a more significant contribution to the MLcps compared to metrics with lower weights. In the case where no weights are assigned (unweighted MLcps), it is equivalent to conducting a weighted analysis where all weights are set to 1.

Datasets

In this study, four distinct datasets were employed to evaluate MLcps (Table 1). The initial dataset comprises mRNA data (n=136) derived from a CLL study, which examined transcriptome profiles in individuals affected by blood cancer[21]. Our objective was to develop a model capable of distinguishing between male and female patients using their transcriptomic profiles. To achieve this, we focused on the top 5,000 most variably expressed mRNAs, excluding genes from the Y chromosome.

The second set of data was obtained from a study on cervical cancer, where the expression levels of 714 miRNAs were measured in human samples (n=58)[22]. The third and fourth datasets were collected from TCGA and involved mRNA (n=1219) and miRNA (n=1207) sequencing of BRCA. The TCGAbiolinks package in R was used to retrieve these datasets[23]. For the BRCA mRNA dataset, we focused on genes that showed differential expression according to edgeR analysis (FDR <= 0.001 and logFC > ±2)[24]. Our objective was to develop a model capable of distinguishing between normal and tumor samples for both the cervical cancer and TCGA-BRCA datasets.

The fifth dataset in our study comprises body signal data collected from 100,000 individuals through the National Health Insurance Service in Korea[25]. This dataset includes 21 essential biological signals related to health, such as measurements of systolic blood pressure and total cholesterol levels. Our main goal with this dataset was to determine whether individuals consume alcohol based on the available biological signal information.

Among these datasets, two were relatively small (CLL and the cervical cancer study), while the other two (TCGA datasets) were imbalanced (Table 1). We utilized an in-house ML pipeline (Figure S5) to train and evaluate eight different models (Table S1) to identify the best-performing model for CLL, cervical cancer, and the TCGA datasets. For the biological signal dataset, we utilized the ‘customML’ feature from the Machine Learning Made Easy (MLme)[26] tool to train and evaluate six different models and identify the best-performing one for classifying alcohol consumers and non-consumers.

Implementation

MLcps is developed using Python[27] and R[28] programming languages. Pandas[29,30] is used to store and process the data. Plotly[31] is used to generate the figures. The radarchart[32] package in R was used for surface area calculation of the polar plane. The R packages tibble[33] and dplyr[34] were utilized for data wrangling in the computation of MLcps during the analysis.

Table 1: Example datasets used in this study.

Dataset	Data type	Number of Samples	Number of Features	Target Class ratio
CLL	mRNA	136	5000	Male (n=82): Female (n=54)
Cervical cancer	miRNA	58	714	Normal (n=29): Tumor (n=29)
TCGA-BRCA	miRNA	1207	1404	Normal (n=104): Tumor (n=1104)
TCGA-BRCA	mRNA	1219	5520	Normal (n=113): Tumor (n=1106)
Body signal	Body signal data (hemoglobin, triglyceride)	100,000	21	**Consume Alcohol** Yes (n=50173): No (n= 49827)

Conclusion

Our paper introduces MLcps, a novel evaluation metric implemented as a Python package. MLcps is a robust evaluation metric designed specifically for classification problems. Its ability to integrate multiple evaluation metrics into a single score makes it an efficient and reliable approach for evaluating model performance and selecting the most successful model. This is especially valuable when multiple evaluation metrics are necessary to fully comprehend a model's strengths and weaknesses.

However, it's essential to understand that the reliability of MLcps depends on the quality of the metrics used in its calculation. Therefore, it is of utmost importance to employ appropriate evaluation metrics, which depend on various factors such as the specific domain, stakeholder preferences, and data characteristics. Similarly, assigning weights to evaluation metrics in machine learning offers a valuable technique for prioritizing specific aspects of model performance, but it comes with potential drawbacks and complexities. For example, heavily weighting one metric can overshadow the overall evaluation, possibly resulting in suboptimal models. Additionally, the assignment of metric weights often depends on subjective judgments regarding their relative significance. Various stakeholders may hold differing perspectives on how much weight to allocate to each metric, potentially leading to evaluation bias.

While the allocation of weights to evaluation metrics can enhance the customization of the evaluation process for specific objectives, it must be executed judiciously, considering the possible downsides and challenges associated with this approach. Striking a balance between highlighting key metrics and maintaining a comprehensive view of model performance is paramount. Therefore, we strongly discourage relying on MLcps without considering the context in which it is applied.

Availability of supporting source code and requirements

Project name: Machine Learning cumulative performance score (MLcps)

Project home page: https://github.com/FunctionalUrology/MLcps

Operating system(s): Platform independent

Programming language: Python >=3.8 and R >=4.0

Other requirements: radarchart, tibble, and dplyr R packages.

License: GNU GPL

BioTool ID: mlcps

RRID: SCR_024716

Data availability

An archival copy of the code and supporting data is available via the GigaScience repository, GigaDB[35]. DOME-ML (Data, Optimisation, Model, and Evaluation in Machine Learning) annotations, supporting the current study, are available via the supporting data in GigaDB.

Authors' Contributions

K.M., A.H.G, and A.A. conceived the idea for the manuscript. A.A. and M.K. wrote the source code in addition to carrying out testing and debugging of the MLcps. K.M., F.C.B, and A.H.G tested the MLcps and provided scientific inputs throughout the development phase. F.C.B, R.M.A and A.B.A provided the feedback on biological application of the tool. N.S and M.A provided the mathematical support and did the testing and debugging. All authors contributed to writing, proofreading, and correcting the manuscript.

Funding

We gratefully acknowledge the financial support of the Swiss National Science Foundation (SNF Grant 310030_175773 to FCB and KM, 212298 to FCB and AHG) and the Wings for Life Spinal Cord Research Foundation (WFL-AT-06/19 to KM). AHG and RMA are supported by R01 DK 077195 and R01 DK127673. MK is supported by the Else Kröner-Fresenius-Stiftung (EKFS 2021_EKeA.33). The authors acknowledge the financial support from the Federal Ministry of Education and Research of Germany and by the Sächsische Staatsministerium für Wissenschaft Kultur und Tourismus in the program Center of Excellence for AI-research "Center for Scalable Data Analytics and Artificial Intelligence Dresden/Leipzig" (project identification number: ScaDS.AI).

Competing Interests

The authors have declared no competing interests.

Acknowledgment

We express our sincere gratitude to Dr. Nezhla Aghaei for their invaluable inspiration, assistance in guiding us through the mathematical formulation and providing expert consultation in the calculation of the planar surface area.

References

1. Sun, Y., Wong, A. K. C. & Kamel, M. S. Classification of imbalanced data: a review. *Int. J. Patt. Recogn. Artif. Intell.* **23**, 687–719 (2009).
2. Russo, D. P., Zorn, K. M., Clark, A. M., Zhu, H. & Ekins, S. Comparing Multiple Machine Learning Algorithms and Metrics for Estrogen Receptor Binding Prediction. *Mol. Pharmaceutics* **15**, 4361–4370 (2018).
3. Stevens, L. M., Mortazavi, B. J., Deo, R. C., Curtis, L. & Kao, D. P. Recommendations for Reporting Machine Learning Analyses in Clinical Research. *Circulation: Cardiovascular Quality and Outcomes* **13**, e006556 (2020).
4. Biswas, A., Saran, I. & Wilson, F. P. Introduction to Supervised Machine Learning. *Kidney360* **2**, 878 (2021).
5. Rashidi, H. H., Albahra, S., Robertson, S., Tran, N. K. & Hu, B. Common statistical concepts in the supervised Machine Learning arena. *Front Oncol* **13**, 1130229 (2023).
6. Hicks, S. A. *et al.* On evaluation metrics for medical applications of artificial intelligence. *Sci Rep* **12**, 5979 (2022).
7. Ahmadzadeh, A., Kempton, D. J., Martens, P. C. & Angryk, R. A. Contingency Space: A Semimetric Space for Classification Evaluation. *IEEE Transactions on Pattern Analysis and Machine Intelligence* **45**, 1501–1513 (2023).
8. Huang, J., Lu, J. & Ling, C. X. Comparing naive Bayes, decision trees, and SVM with AUC and accuracy. in *Third IEEE International Conference on Data Mining* 553–556 (2003). doi:10.1109/ICDM.2003.1250975.
9. Provost, F. & Domingos, P. Tree Induction for Probability-Based Ranking. *Machine Learning* **52**, 199–215 (2003).
10. Huang, J. & Ling, C. X. Using AUC and accuracy in evaluating learning algorithms. *IEEE Transactions on Knowledge and Data Engineering* **17**, 299–310 (2005).
11. Jeni, L. A., Cohn, J. F. & De La Torre, F. Facing Imbalanced Data–Recommendations for the Use of Performance Metrics. in *2013 Humaine Association Conference on Affective Computing and Intelligent Interaction* 245–251 (2013). doi:10.1109/ACII.2013.47.
12. Stafford, I. S. *et al.* A systematic review of the applications of artificial intelligence and machine learning in autoimmune diseases. *NPJ Digit Med* **3**, 30 (2020).
13. Zhou, J., Gandomi, A. H., Chen, F. & Holzinger, A. Evaluating the Quality of Machine Learning Explanations: A Survey on Methods and Metrics. *Electronics* **10**, 593 (2021).
14. Adhikari, S., Normand, S.-L., Bloom, J., Shahian, D. & Rose, S. Revisiting performance metrics for prediction with rare outcomes. *Stat Methods Med Res* **30**, 2352–2366 (2021).
15. Rácz, A., Bajusz, D. & Héberger, K. Multi-Level Comparison of Machine Learning Classifiers and Their Performance Metrics. *Molecules* **24**, 2811 (2019).
16. Branco, P., Torgo, L. & Ribeiro, R. P. A Survey of Predictive Modeling on Imbalanced Domains. *ACM Comput. Surv.* **49**, 31:1-31:50 (2016).
17. Chakrabarti, S., Khanna, R., Sawant, U. & Bhattacharyya, C. Structured learning for non-smooth ranking losses. in *Proceedings of the 14th ACM SIGKDD*

international conference on Knowledge discovery and data mining 88–96 (Association for Computing Machinery, 2008). doi:10.1145/1401890.1401906.

18. Geng, X. & Cheng, X.-Q. Learning multiple metrics for ranking. *Front. Comput. Sci. China* **5**, 259–267 (2011).
19. Galar, M., Fernandez, A., Barrenechea, E., Bustince, H. & Herrera, F. A Review on Ensembles for the Class Imbalance Problem: Bagging-, Boosting-, and Hybrid-Based Approaches. *IEEE Transactions on Systems, Man, and Cybernetics, Part C (Applications and Reviews)* **42**, 463–484 (2012).
20. Uzun Ozsahin, D., Onakpojeruo, E. P., Uzun, B., Mustapha, M. T. & Ozsahin, I. Mathematical Assessment of Machine Learning Models Used for Brain Tumor Diagnosis. *Diagnostics (Basel)* **13**, 618 (2023).
21. Dietrich, S. *et al.* Drug-perturbation-based stratification of blood cancer. *J Clin Invest* **128**, 427–445 (2018).
22. Witten, D., Tibshirani, R., Gu, S. G., Fire, A. & Lui, W.-O. Ultra-high throughput sequencing-based small RNA discovery and discrete statistical biomarker analysis in a collection of cervical tumours and matched controls. *BMC Biology* **8**, 58 (2010).
23. Colaprico, A. *et al.* TCGAbiolinks: an R/Bioconductor package for integrative analysis of TCGA data. *Nucleic Acids Research* **44**, e71 (2016).
24. Robinson, M. D., McCarthy, D. J. & Smyth, G. K. edgeR: a Bioconductor package for differential expression analysis of digital gene expression data. *Bioinformatics* **26**, 139–140 (2010).
25. Her, S. Smoking and Drinking Dataset with body signal. *Kaggle* https://www.kaggle.com/datasets/sooyoungher/smoking-drinking-dataset.
26. Akshay, A. *et al.* Machine Learning Made Easy (MLme): A Comprehensive Toolkit for Machine Learning-Driven Data Analysis. 2023.07.04.546825 Preprint at https://doi.org/10.1101/2023.07.04.546825 (2023).
27. van Rossum, G. Python reference manual. (1995).
28. R Core Team. *R: A Language and Environment for Statistical Computing*. (R Foundation for Statistical Computing, 2022).
29. McKinney, W. Data Structures for Statistical Computing in Python. *Proceedings of the 9th Python in Science Conference* 56–61 (2010) doi:10.25080/Majora-92bf1922-00a.
30. The pandas development team. pandas-dev/pandas: Pandas. (2020) doi:10.5281/zenodo.3509134.
31. Inc, P. T. Collaborative data science. https://plot.ly (2015).
32. Porter, D. A. S. radarchart: Radar Chart from 'Chart.js'. *R Package* **0.3.1**, (2016).
33. Müller, K. & Wickham, H. *tibble: Simple Data Frames*. (2023).
34. Wickham, H., François, R., Henry, L., Müller, K. & Vaughan, D. *dplyr: A Grammar of Data Manipulation*. (2023).
35. Akshay A, Abedi M, Shekarchizadeh N, Burkhard FC, Katoch M, Bigger-Allen A et al. Supporting data for 'MLcps: Machine Learning Cumulative Performance Score for Classification Problems'. GigaScience Database. 2023. http://dx.doi.org/10.5524/102471

Figure_Final

Click here to access/download;Figure;figure_main.pptx

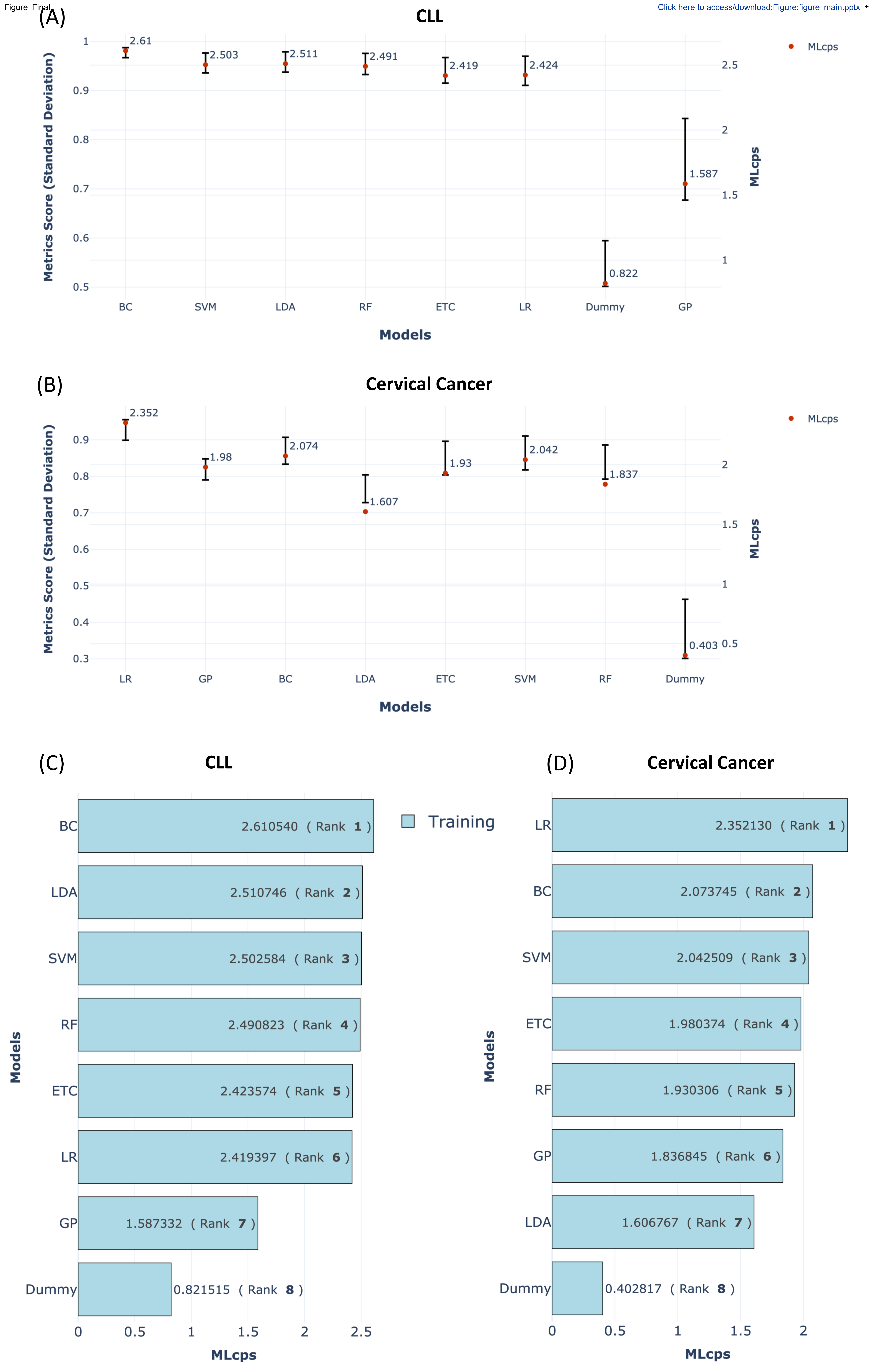

Figure 1. Standard deviation (SD) of Performance Metrics and MLcps Comparison for CLL and Cervical Cancer Datasets. Figure (A) and (B) illustrate the SD of performance metric scores for ML algorithms trained on the CLL and Cervical Cancer datasets, respectively. The bars in the plot represent the SD of performance metric scores and are displayed on the left y-axis. The bars are arranged from left to right, with smaller SD values on the left and larger SD values on the right. A red dot on the plot represents the MLcps which is displayed on the right y-axis. Figure (C) and (D), represent MLcps for training data from the CLL and Cervical Cancer datasets, respectively. The numerical MLcps values are indicated within each bar. Rankings, enclosed in brackets, reflect model performance based on MLcps.

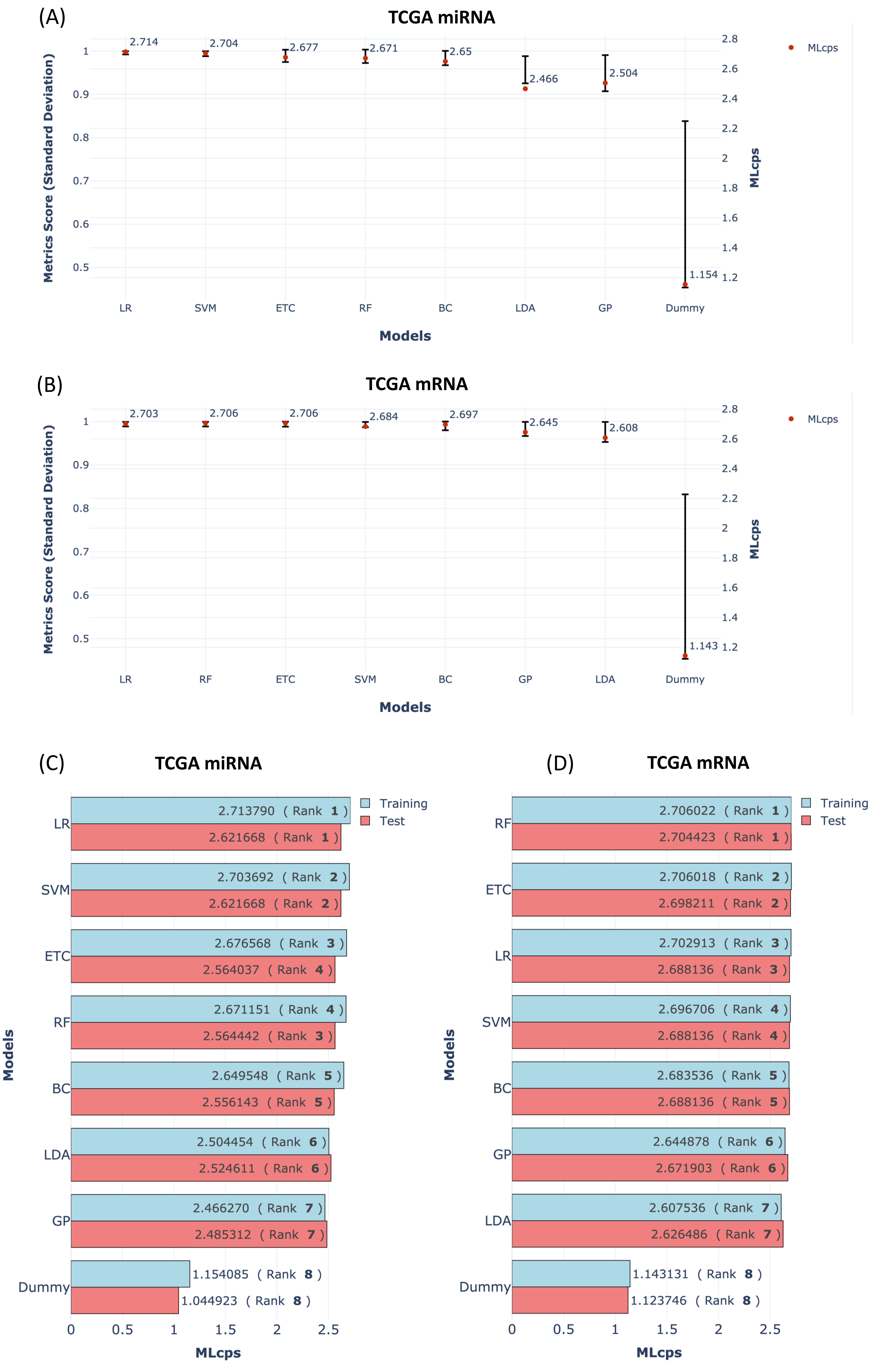

Figure 2. Standard deviation (SD) of Performance Metrics and MLcps Comparison for TCGA mRNA and miRNA Datasets. Figure (A) and (B) illustrate the SD of performance metric scores for ML algorithms trained on the mRNA and miRNA datasets, respectively. The bars in the plot represent the SD of performance metric scores and are displayed on the left y-axis. The bars are arranged from left to right, with smaller SD values on the left and larger SD values on the right. A red dot on the plot represents the MLcps which is displayed on the right y-axis. Figure (C) and (D), represent a comparison of MLcps for training and test data from the mRNA and miRNA datasets, respectively. The numerical MLcps values are indicated within each bar. Rankings, enclosed in brackets, reflect model performance based on MLcps, whether computed from the training or test data.

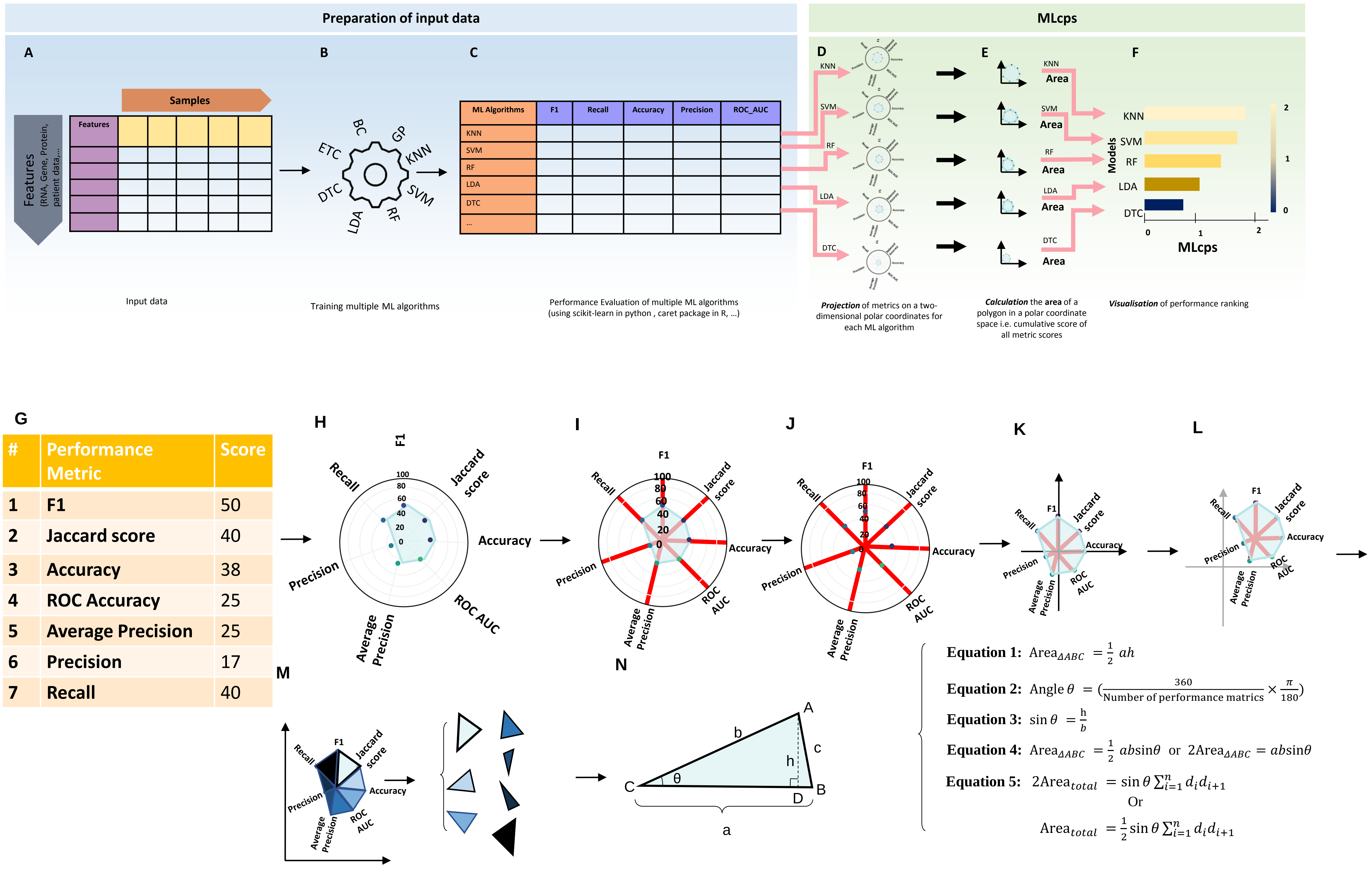

Figure 3 Schematic overview of the complete analysis process for MLcps Python package. Before using the MLcps Python package, one needs to prepare the raw data (A). This input table can be RNA sequencing, proteomics, patients' profile, molecular data, etc (normally this data is in txt or csv format). Next step is to perform multiple ML algorithms (B). Performing this step can be done by any package or programming language of choice. The next step is to evaluate the performance of the ML algorithms. We recommend the use of multiple metrics such as F1, Recall, etc (C). The performance metric scores then need to be arranged in a tabular format as depicted in (C). This table will be used as an input for the MLcps package. From here on the MLcps will process the data. MLcps involves three steps: projection, calculation, and visualization (PCV). To calculate the cumulative score of each ML algorithm in the input data, MLcps first projects the performance metric onto the two-dimensional polar coordinates system (D). Next, the projected polygon's area is calculated (E). Finally, the user can visualize this MLcps to rank the performance of given ML algorithms (F). The lower panel (G-N) visualises the procedure to calculate the surface area as cumulative score in detail. The names of the algorithms are just mentioned as example and other algorithm can be used too. ETC: Extra Trees Classifier, BC: Bagging Classifier, GP: Gaussian Process Classifier, KNN: K-Nearest Neighbors, SVM: Support Vector Machine, RF: Random Forest Classifier, LDA: Linear Discriminant Analysis, DTC: Decision Tree Classifier.

Click here to access/download
Supplementary Material
suppl.docx

Click here to access/download
Supplementary Material
figure_supp.pptx
