# Supplementary material for: MLcps: machine learning cumulative performance score for classification problems
Source: Gigascience. 2023 Dec 13;12:giad108. doi: 10.1093/gigascience/giad108 (PMC10716825; doi:10.1093/gigascience/giad108)
Supplement: giad108_Supplemental_Files [file giad108_supplemental_files.zip › figure_supp.pptx]

## Slide 1
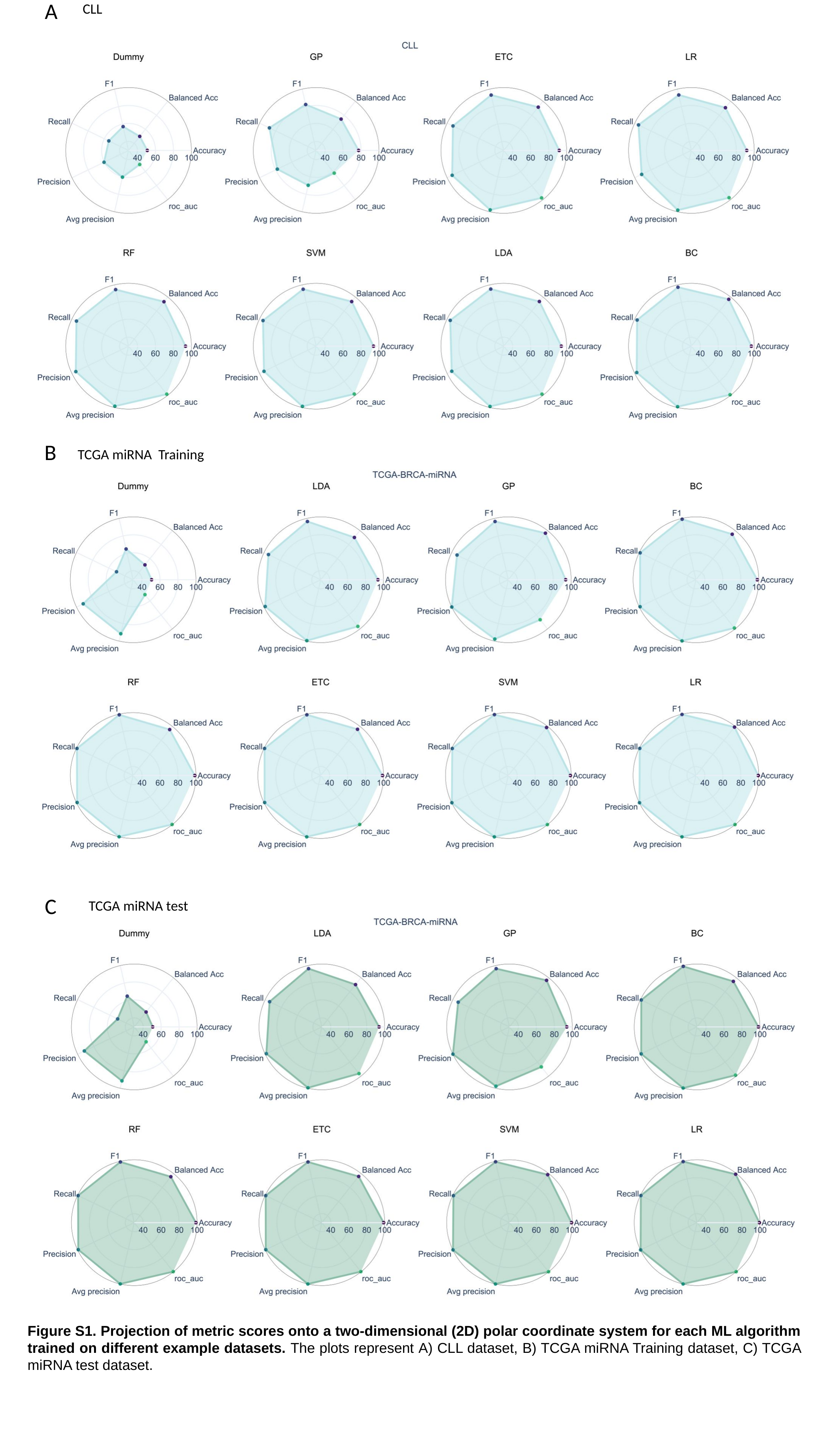

A
CLL
B
TCGA miRNA Training
C
TCGA miRNA test
Figure S1. Projection of metric scores onto a two-dimensional (2D) polar coordinate system for each ML algorithm trained on different example datasets. The plots represent A) CLL dataset, B) TCGA miRNA Training dataset, C) TCGA miRNA test dataset.

## Slide 2
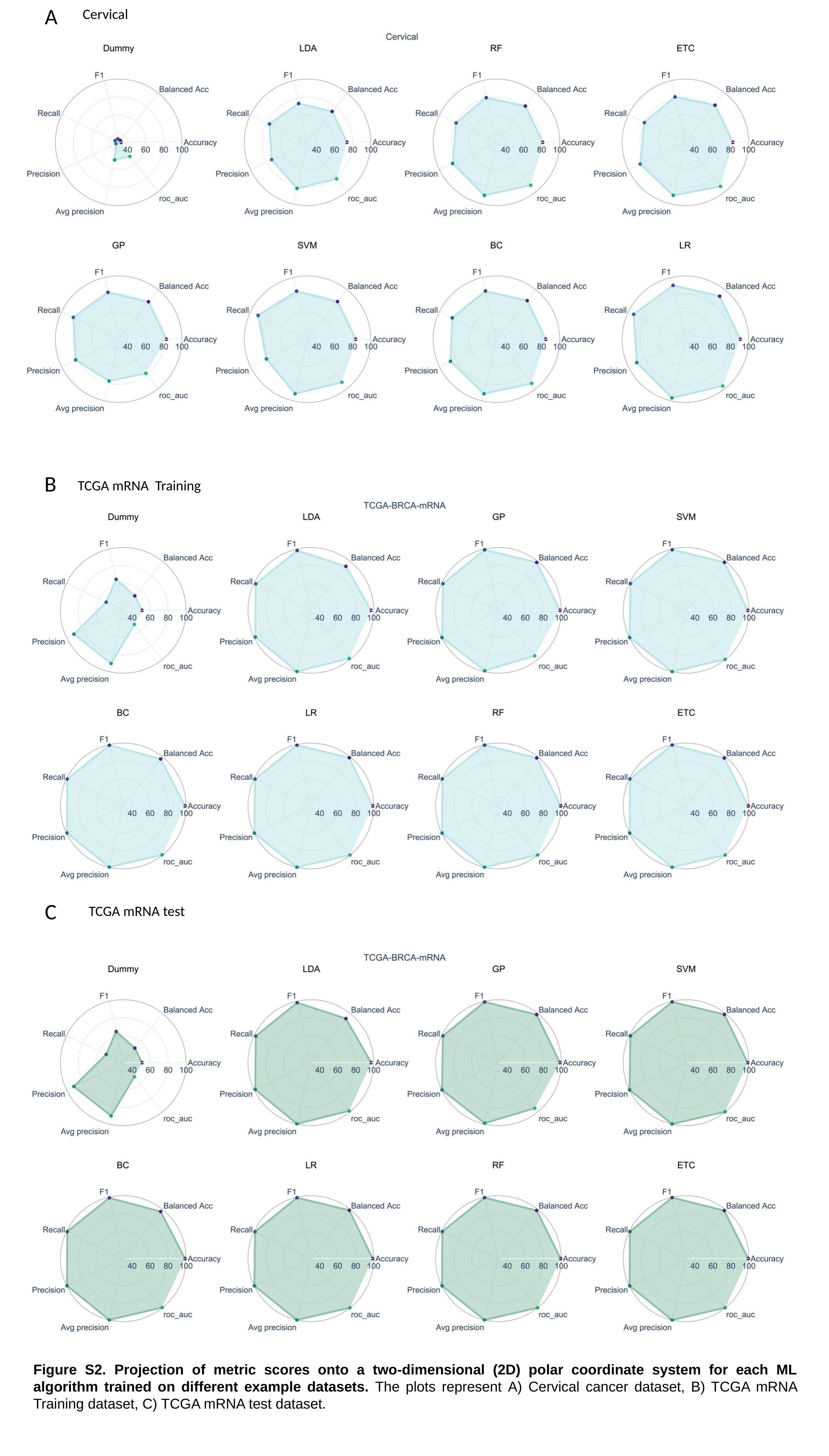

A
Cervical
B
TCGA mRNA Training
C
TCGA mRNA test
Figure S2. Projection of metric scores onto a two-dimensional (2D) polar coordinate system for each ML algorithm trained on different example datasets. The plots represent A) Cervical cancer dataset, B) TCGA mRNA Training dataset, C) TCGA mRNA test dataset.

## Slide 3
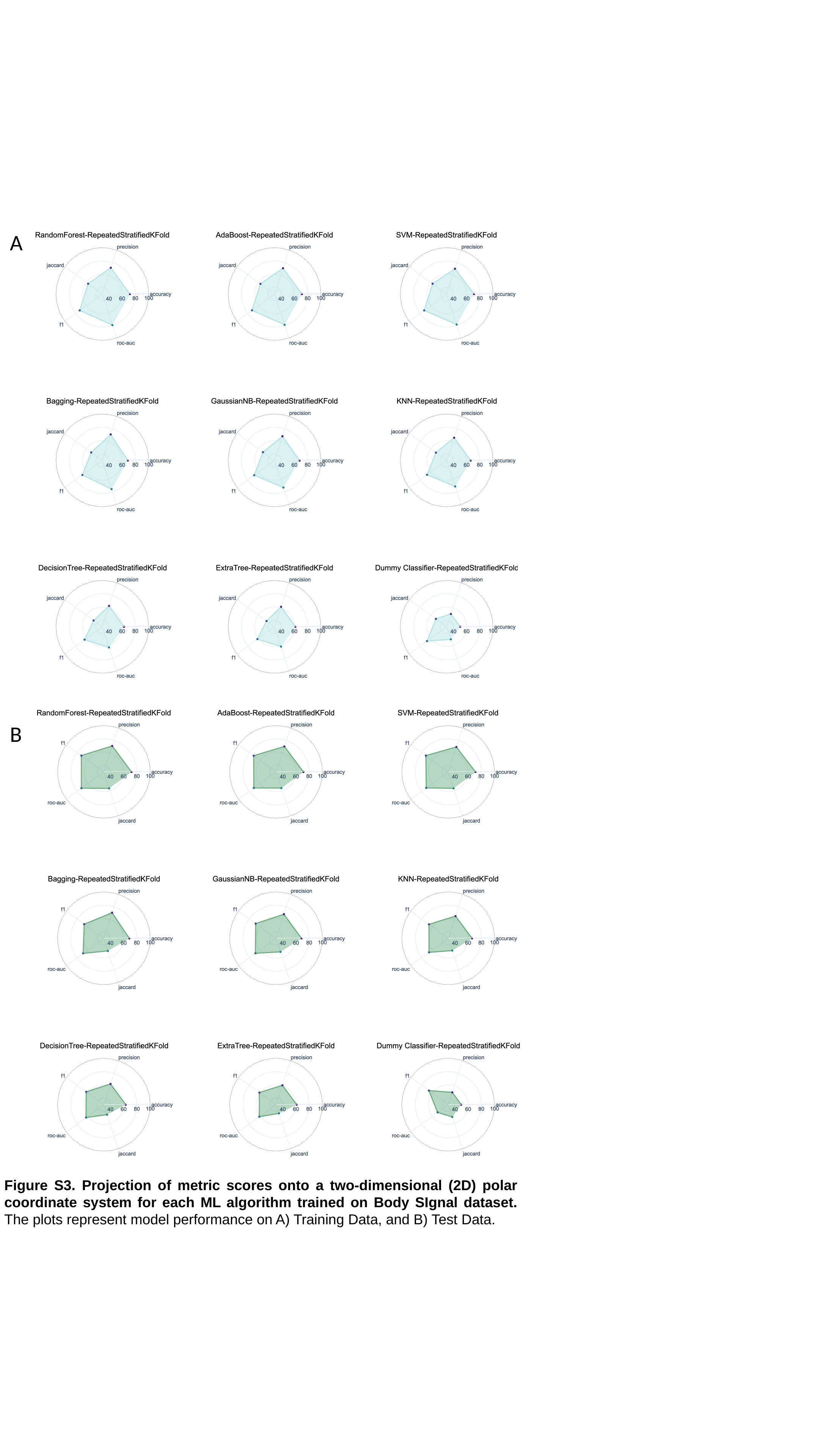

A
B
Figure S3. Projection of metric scores onto a two-dimensional (2D) polar coordinate system for each ML algorithm trained on Body SIgnal dataset. The plots represent model performance on A) Training Data, and B) Test Data.

## Slide 4
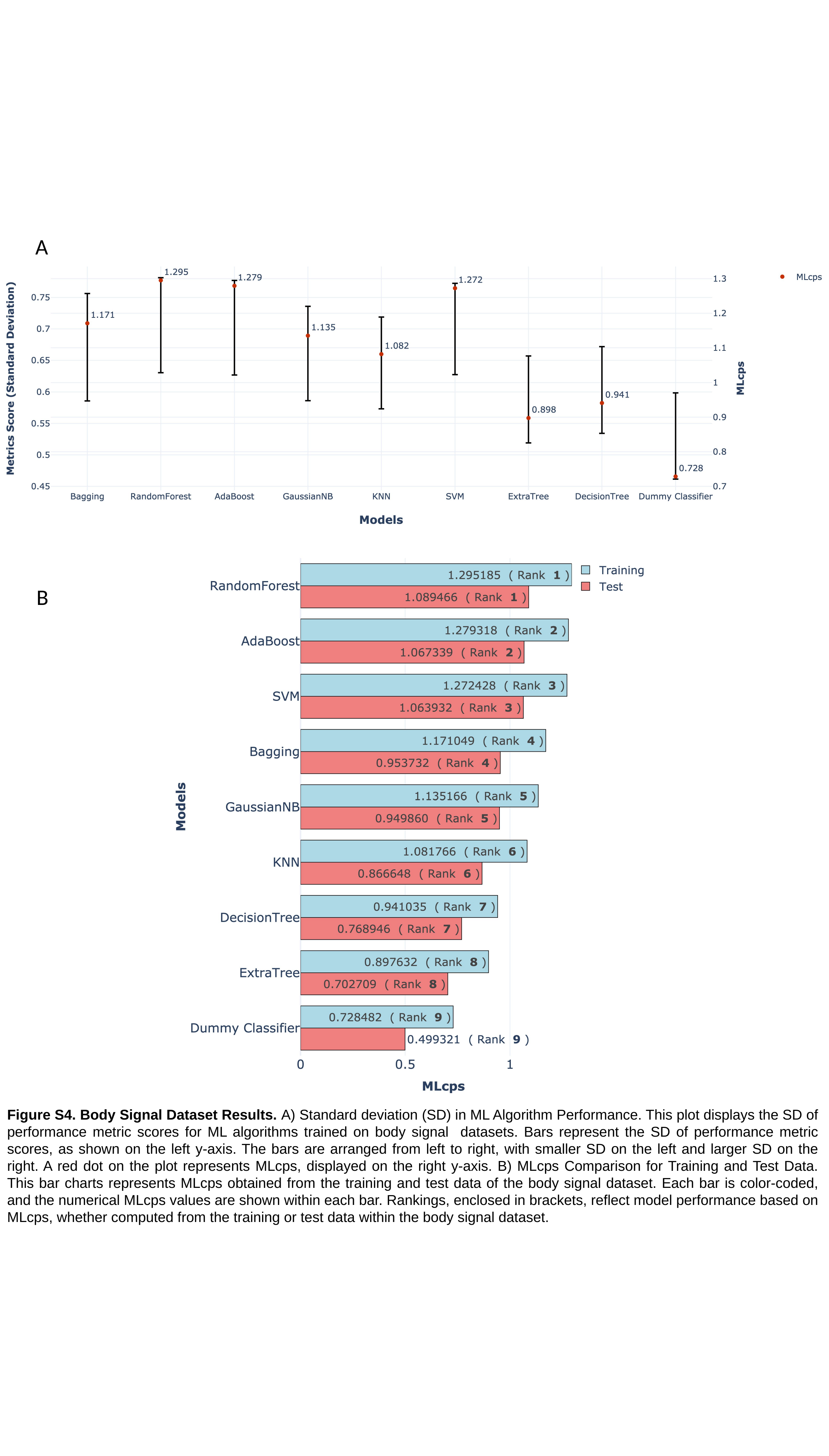

A
B
Figure S4. Body Signal Dataset Results. A) Standard deviation (SD) in ML Algorithm Performance. This plot displays the SD of performance metric scores for ML algorithms trained on body signal datasets. Bars represent the SD of performance metric scores, as shown on the left y-axis. The bars are arranged from left to right, with smaller SD on the left and larger SD on the right. A red dot on the plot represents MLcps, displayed on the right y-axis. B) MLcps Comparison for Training and Test Data. This bar charts represents MLcps obtained from the training and test data of the body signal dataset. Each bar is color-coded, and the numerical MLcps values are shown within each bar. Rankings, enclosed in brackets, reflect model performance based on MLcps, whether computed from the training or test data within the body signal dataset.

## Slide 5
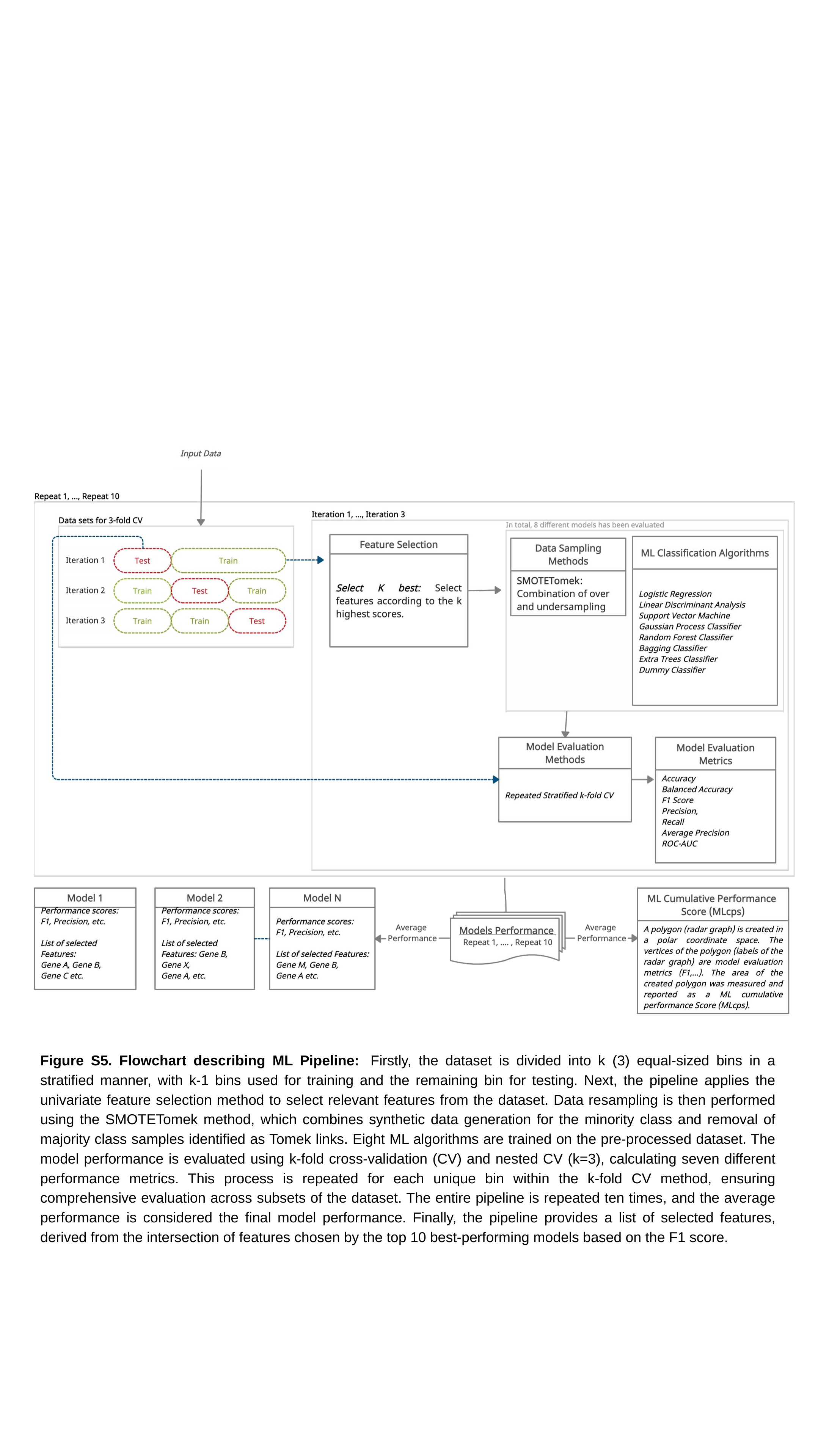

Figure S5. Flowchart describing ML Pipeline:  Firstly, the dataset is divided into k (3) equal-sized bins in a stratified manner, with k-1 bins used for training and the remaining bin for testing. Next, the pipeline applies the univariate feature selection method to select relevant features from the dataset. Data resampling is then performed using the SMOTETomek method, which combines synthetic data generation for the minority class and removal of majority class samples identified as Tomek links. Eight ML algorithms are trained on the pre-processed dataset. The model performance is evaluated using k-fold cross-validation (CV) and nested CV (k=3), calculating seven different performance metrics. This process is repeated for each unique bin within the k-fold CV method, ensuring comprehensive evaluation across subsets of the dataset. The entire pipeline is repeated ten times, and the average performance is considered the final model performance. Finally, the pipeline provides a list of selected features, derived from the intersection of features chosen by the top 10 best-performing models based on the F1 score.
