# Supplementary material for: MLcps: machine learning cumulative performance score for classification problems
Source: Gigascience. 2023 Dec 13;12:giad108. doi: 10.1093/gigascience/giad108 (PMC10716825; doi:10.1093/gigascience/giad108)
Supplement: giad108_Supplemental_Files [file giad108_supplemental_files.zip › suppl.docx]

**Supplementary**

**MLcps: Machine Learning Cumulative Performance Score for classification problems**

Akshay Akshay ^1,2^, Masoud Abedi^3^, Navid Shekarchizadeh^3,4^, Fiona C. Burkhard^1,5^, Mitali Katoch^6^, Alex Bigger-Allen ^7,8,9,10^, Rosalyn M. Adam ^8,9,10^, Katia Monastyrskaya ^1, 5^ and Ali Hashemi Gheinani ^1,5,8,9,10^*

^1^ Functional Urology Research Group, Department for BioMedical Research DBMR, University of Bern, Switzerland

^2^ Graduate School for Cellular and Biomedical Sciences, University of Bern, Switzerland

^3^Department of Medical Data Science, Leipzig University Medical Centre, 04107 Leipzig, Germany

^4^Center for Scalable Data Analytics and Artificial Intelligence (ScaDS.AI) Dresden/Leipzig, 04105 Leipzig, Germany

^5^ Department of Urology, Inselspital University Hospital, 3010 Bern, Switzerland

^6^ Institute of Neuropathology, Universitätsklinikum Erlangen, Friedrich-Alexander-Universität Erlangen-Nürnberg (FAU), Erlangen, Germany

^7^ Biological & Biomedical Sciences Program, Division of Medical Sciences, Harvard Medical School, Boston, MA.

^8^ Urological Diseases Research Center, Boston Children’s Hospital, MA, USA

^9^ Harvard Medical School, Boston, Department of Surgery MA, USA

^10^ Broad Institute of MIT and Harvard, Cambridge, MA, USA

* Corresponding author:

Ali Hashemi Gheinani, Urological Diseases Research Center, Boston Children’s Hospital, Harvard Medical School and Broad Institute of MIT and Harvard, Cambridge, MA, USA

e-mail: [Ali.HashemiGheinani@childrens.harvard.edu](mailto:Ali.HashemiGheinani@childrens.harvard.edu)

**Machine Learning Pipeline**

The proposed pipeline (Figure S3) begins by splitting the input datasets into k (3) equally sized bins in a stratified manner. Out of these, k-1 bins are used as training datasets, while the remaining bin serves as the test dataset. The pipeline then employs the SelectKBest method to select features from a given dataset. To address the issue of imbalanced datasets, the pipeline utilizes the SMOTETomek method, which combines both over- and under-sampling techniques [1]. SMOTETomek generates synthetic data for the minority class using SMOTE and removes data points from the majority class if they are identified as Tomek links. Tomek links refer to samples of data from the majority class that are in close proximity to the minority class data.

In the subsequent step, the pipeline proceeds to train multiple machine learning (ML) algorithms for the given problem. ML algorithms can be broadly classified into three categories: a) Linear Algorithms, b) Nonlinear Algorithms, and c) Ensemble Algorithms. For this study, we have selected at least one classification algorithm from each of these categories. Specifically, a total of seven different classification algorithms were trained for each dataset (Table S1). Additionally, a Dummy classifier, which generates random predictions, was used as a baseline for comparison with the other models.

*Table S1: ML algorithms used in this study.*

| **Classification Algorithm** | **Abbreviation** |
| --- | --- |
| Logistic Regression | LR |
| Linear Discriminant Analysis | LDA |
| Support Vector Machine | SVM |
| Gaussian Process Classifier | GP |
| Random Forest Classifier | RF |
| Bagging Classifier | BC |
| Extra Trees Classifier | ETC |

The performance of the trained ML models was assessed using the k-fold cross-validation (CV) method, where k was set to 3. This approach involves dividing the entire dataset into k non-overlapping subsets of equal size. For each fold, (k - 1) subsets were utilized as the training dataset for the model, while the remaining subset served as the test dataset for evaluating the model's performance. This process was repeated k times, resulting in the training of k different models. The final performance of the model was estimated by calculating the average of the evaluation metrics obtained from each iteration. To account for the potential variability in the estimated model performance from a single run of the k-fold cross-validation, the process was repeated n times. In this study, the k-fold CV was repeated 10 times to ensure robustness and obtain a more reliable estimate of the model's performance.

In addition to the k-fold cross-validation, larger TCGA-BRCA datasets provided the opportunity to create an independent test set, comprising 30% of the datasets. This independent test set allowed us to measure the performance of the trained model on unseen data specifically from the TCGA-BRCA datasets. Similar to ML algorithms, several different performance metrics that *are Accuracy, Balanced Accuracy, Precision, Recall, Average Precision, and ROC-AUC score,* were used to evaluate the performance of ML models. The complete pipeline was developed using the scikit-learn library [2], while the imblearn package [3] was utilized for handling imbalanced datasets. Pandas package was used to store and process the data.

**Python (ML pipeline) Session Information**

| ----- imblearn 0.9.1 ipykernel 6.13.0 joblib 1.1.0 matplotlib 3.5.2 numpy 1.22.3 pandas 1.4.2 plotly 5.8.0 session_info 1.0.0 sklearn 1.1.0 ----- IPython 8.3.0 jupyter_client 7.3.1 jupyter_core 4.10.0 ----- Python 3.10.4 \| packaged by conda-forge \| (main, Mar 24 2022, 17:39:04) [GCC 10.3.0] Linux-3.10.0-1160.62.1.el7.x86_64-x86_64-with-glibc2.17 ----- Session information updated at 2022-06-08 17:31 |
| --- |

**Packages and other dependencies used in MLcps**

**Python**

| ----- MLcps 0.0.5 numpy 1.22.3 pandas 1.4.2 pkg_resources NA plotly 5.8.0 rpy2 3.5.1 session_info 1.0.0 ----- IPython 7.33.0 jupyter_client 7.3.1 jupyter_core 4.10.0 ----- Python 3.10.4 \| packaged by conda-forge \| (main, Mar 24 2022, 17:45:10) [Clang 12.0.1 ] macOS-11.2.3-x86_64-i386-64bit ----- Session information updated at 2022-06-08 10:07 |
| --- |

**R**

| **Package** | **Version** | **Reference** |
| --- | --- | --- |
| **tidyselect** | 1.1.2 | Lionel Henry and Hadley Wickham (2022). tidyselect: Select from a Set of Strings. R package version 1.1.2. [https://CRAN.R-project.org/package=tidyselect](https://cran.r-project.org/package=tidyselect) |
| **purrr** | 0.3.4 | Lionel Henry and Hadley Wickham (2020). purrr: Functional Programming Tools. R package version 0.3.4. [https://CRAN.R-project.org/package=purrr](https://cran.r-project.org/package=purrr) |
| **haven** | 2.4.1 | Hadley Wickham and Evan Miller (2021). haven: Import and Export 'SPSS', 'Stata' and 'SAS' Files. R package version 2.4.1. [https://CRAN.R-project.org/package=haven](https://cran.r-project.org/package=haven) |
| **carData** | 3.0.4 | John Fox, Sanford Weisberg and Brad Price (2020). carData: Companion to Applied Regression Data Sets. R package version 3.0-4. [https://CRAN.R-project.org/package=carData](https://cran.r-project.org/package=carData) |
| **colorspace** | 2.0.2 | Not available |
| **vctrs** | 0.4.1 | Hadley Wickham, Lionel Henry and Davis Vaughan (2022). vctrs: Vector Helpers. R package version 0.4.1. [https://CRAN.R-project.org/package=vctrs](https://cran.r-project.org/package=vctrs) |
| **generics** | 0.1.2 | Hadley Wickham, Max Kuhn and Davis Vaughan (2022). generics: Common S3 Generics not Provided by Base R Methods Related to |
| **htmltools** | 0.5.1.1 | Joe Cheng, Carson Sievert, Winston Chang, Yihui Xie and Jeff Allen (2021). htmltools: Tools for HTML. R package version 0.5.1.1. [https://CRAN.R-project.org/package=htmltools](https://cran.r-project.org/package=htmltools) |
| **utf8** | 1.2.1 | Patrick O. Perry (2021). utf8: Unicode Text Processing. R package version 1.2.1. [https://CRAN.R-project.org/package=utf8](https://cran.r-project.org/package=utf8) |
| **rlang** | 1.0.2 | Lionel Henry and Hadley Wickham (2022). rlang: Functions for Base Types and Core R and 'Tidyverse' Features. R package version 1.0.2. [https://CRAN.R-project.org/package=rlang](https://cran.r-project.org/package=rlang) |
| **pillar** | 1.7.0 | Kirill Müller and Hadley Wickham (2022). pillar: Coloured Formatting for Columns. R package version 1.7.0. [https://CRAN.R-project.org/package=pillar](https://cran.r-project.org/package=pillar) |
| **foreign** | 0.8.81 | R Core Team (2020). foreign: Read Data Stored by 'Minitab', 'S', 'SAS', 'SPSS', 'Stata', |
| **glue** | 1.6.2 | Jim Hester and Jennifer Bryan (2022). glue: Interpreted String Literals. R package version 1.6.2. [https://CRAN.R-project.org/package=glue](https://cran.r-project.org/package=glue) |
| **withr** | 2.4.2 | Jim Hester, Kirill Müller, Kevin Ushey, Hadley Wickham and Winston Chang (2021). withr: Run Code 'With' Temporarily Modified Global State. R package version 2.4.2. [https://CRAN.R-project.org/package=withr](https://cran.r-project.org/package=withr) |
| **DBI** | 1.1.1 | R Special Interest Group on Databases (R-SIG-DB), Hadley Wickham and Kirill Müller (2021). DBI: R Database Interface. R package version 1.1.1. [https://CRAN.R-project.org/package=DBI](https://cran.r-project.org/package=DBI) |
| **RColorBrewer** | 1.1.2 | Erich Neuwirth (2014). RColorBrewer: ColorBrewer Palettes. R package version 1.1-2. [https://CRAN.R-project.org/package=RColorBrewer](https://cran.r-project.org/package=RColorBrewer) |
| **readxl** | 1.3.1 | Hadley Wickham and Jennifer Bryan (2019). readxl: Read Excel Files. R package version 1.3.1. [https://CRAN.R-project.org/package=readxl](https://cran.r-project.org/package=readxl) |
| **lifecycle** | 1.0.1 | Lionel Henry and Hadley Wickham (2021). lifecycle: Manage the Life Cycle of your Package Functions. R package version 1.0.1. [https://CRAN.R-project.org/package=lifecycle](https://cran.r-project.org/package=lifecycle) |
| **plyr** | 1.8.6 | Hadley Wickham (2011). The Split-Apply-Combine Strategy for Data Analysis. Journal of Statistical Software, 40(1), 1-29. URL<http://www.jstatsoft.org/v40/i01/>. |
| **cellranger** | 1.1.0 | Jennifer Bryan (2016). cellranger: Translate Spreadsheet Cell Ranges to Rows and Columns. R package version 1.1.0. [https://CRAN.R-project.org/package=cellranger](https://cran.r-project.org/package=cellranger) |
| **munsell** | 0.5.0 | Charlotte Wickham (2018). munsell: Utilities for Using Munsell Colours. R package version 0.5.0. [https://CRAN.R-project.org/package=munsell](https://cran.r-project.org/package=munsell) |
| **ggsignif** | 0.6.2 | Ahlmann-Eltze, C., & Patil, I. (2021). ggsignif: R Package for Displaying Significance Brackets for 'ggplot2'. PsyArxiv. doi:10.31234/[osf.io/7awm6](http://osf.io/7awm6) |
| **gtable** | 0.3.0 | Hadley Wickham and Thomas Lin Pedersen (2019). gtable: Arrange 'Grobs' in Tables. R package version 0.3.0. [https://CRAN.R-project.org/package=gtable](https://cran.r-project.org/package=gtable) |
| **zip** | 2.2.0 | Gábor Csárdi, Kuba Podgórski and Rich Geldreich (2021). zip: Cross-Platform 'zip' Compression. R package version 2.2.0. [https://CRAN.R-project.org/package=zip](https://cran.r-project.org/package=zip) |
| **htmlwidgets** | 1.5.3 | Ramnath Vaidyanathan, Yihui Xie, JJ Allaire, Joe Cheng, Carson Sievert and Kenton Russell (2020). htmlwidgets: HTML Widgets for R. R package version 1.5.3. [https://CRAN.R-project.org/package=htmlwidgets](https://cran.r-project.org/package=htmlwidgets) |
| **forcats** | 0.5.1 | Hadley Wickham (2021). forcats: Tools for Working with Categorical Variables (Factors). R package version 0.5.1. [https://CRAN.R-project.org/package=forcats](https://cran.r-project.org/package=forcats) |
| **rio** | 0.5.27 | Chung-hong Chan, Geoffrey CH Chan, Thomas J. Leeper, and Jason Becker (2021). rio: A Swiss-army knife for data file I/O. R package version 0.5.27. |
| **extrafont** | 0.17 | Winston Chang, (2014). extrafont: Tools for using fonts. R package version 0.17. [https://CRAN.R-project.org/package=extrafont](https://cran.r-project.org/package=extrafont) |
| **curl** | 4.3.2 | Jeroen Ooms (2021). curl: A Modern and Flexible Web Client for R. R package version 4.3.2. [https://CRAN.R-project.org/package=curl](https://cran.r-project.org/package=curl) |
| **fansi** | 0.5.0 | Brodie Gaslam (2021). fansi: ANSI Control Sequence Aware String Functions. R package version 0.5.0. [https://CRAN.R-project.org/package=fansi](https://cran.r-project.org/package=fansi) |
| **Rttf2pt1** | 1.3.8 | Winston Chang, Andrew Weeks, Frank M. Siegert, Mark Heath, Thomas Henlick, Sergey Babkin, Turgut Uyar, Rihardas Hepas, Szalay Tamas, Johan Vromans, Petr Titera, Lei Wang, Chen Xiangyang, Zvezdan Petkovic, Rigel and I. Lee Hetherington (2020). Rttf2pt1: 'ttf2pt1' Program. R package version 1.3.8. [https://CRAN.R-project.org/package=Rttf2pt1](https://cran.r-project.org/package=Rttf2pt1) |
| **broom** | 0.7.12 | David Robinson, Alex Hayes and Simon Couch (2022). broom: Convert Statistical Objects into Tidy Tibbles. R package version 0.7.12. [https://CRAN.R-project.org/package=broom](https://cran.r-project.org/package=broom) |
| **Rcpp** | 1.0.7 | Dirk Eddelbuettel and Romain Francois (2011). Rcpp: Seamless R and C++ Integration. Journal of Statistical Software, 40(8), 1-18. URL<https://www.jstatsoft.org/v40/i08/>. |
| **KernSmooth** | 2.23.18 | Matt Wand (2020). KernSmooth: Functions for Kernel Smoothing Supporting Wand & Jones (1995). R package version 2.23-18. [https://CRAN.R-project.org/package=KernSmooth](https://cran.r-project.org/package=KernSmooth) |
| **backports** | 1.2.1 | Michel Lang and R Core Team (2020). backports: Reimplementations of Functions Introduced Since R-3.0.0. R package version 1.2.1. [https://CRAN.R-project.org/package=backports](https://cran.r-project.org/package=backports) |
| **abind** | 1.4.5 | Tony Plate and Richard Heiberger (2016). abind: Combine Multidimensional Arrays. R package version 1.4-5. [https://CRAN.R-project.org/package=abind](https://cran.r-project.org/package=abind) |
| **proj4** | 1.0.10.1 | Simon Urbanek (2021). proj4: A simple interface to the PROJ.4 cartographic projections |
| **hms** | 1.1.0 | Kirill Müller (2021). hms: Pretty Time of Day. R package version 1.1.0. [https://CRAN.R-project.org/package=hms](https://cran.r-project.org/package=hms) |
| **digest** | 0.6.27 | Dirk Eddelbuettel with contributions by Antoine Lucas, Jarek Tuszynski, Henrik Bengtsson, Simon Urbanek, Mario Frasca, Bryan Lewis, Murray Stokely, Hannes Muehleisen, Duncan Murdoch, Jim Hester, Wush Wu, Qiang Kou, Thierry Onkelinx, Michel Lang, Viliam Simko, Kurt Hornik, Radford Neal, Kendon Bell, Matthew de Queljoe, Ion Suruceanu, Bill Denney, Dirk Schumacher and and Winston Chang. (2020). digest: Create Compact Hash Digests of R Objects. R package version 0.6.27. [https://CRAN.R-project.org/package=digest](https://cran.r-project.org/package=digest) |
| **openxlsx** | 4.2.4 | Philipp Schauberger and Alexander Walker (2021). openxlsx: Read, Write and Edit xlsx Files. R package version 4.2.4. [https://CRAN.R-project.org/package=openxlsx](https://cran.r-project.org/package=openxlsx) |
| **stringi** | 1.7.2 | Not available |
| **rstatix** | 0.7.0 | Alboukadel Kassambara (2021). rstatix: Pipe-Friendly Framework for Basic Statistical Tests. R package version 0.7.0. [https://CRAN.R-project.org/package=rstatix](https://cran.r-project.org/package=rstatix) |
| **ash** | 1.0.15 | S original by David W. Scott R port by Albrecht Gebhardt adopted to recent S-PLUS by Stephen Kaluzny <spk@insightful.com> (2015). ash: David Scott's ASH Routines. R package version 1.0-15. [https://CRAN.R-project.org/package=ash](https://cran.r-project.org/package=ash) |
| **grid** | 4.0.5 | R Core Team (2021). R: A language and environment for statistical computing. R Foundation for Statistical Computing, Vienna, Austria. URL [https://www.R-project.org/](https://www.r-project.org/). |
| **cli** | 3.2.0 | Gábor Csárdi (2022). cli: Helpers for Developing Command Line Interfaces. R package version 3.2.0. [https://CRAN.R-project.org/package=cli](https://cran.r-project.org/package=cli) |
| **tools** | 4.0.5 | R Core Team (2021). R: A language and environment for statistical computing. R Foundation for Statistical Computing, Vienna, Austria. URL [https://www.R-project.org/](https://www.r-project.org/). |
| **magrittr** | 2.0.1 | Stefan Milton Bache and Hadley Wickham (2020). magrittr: A Forward-Pipe Operator for R. R package version 2.0.1. [https://CRAN.R-project.org/package=magrittr](https://cran.r-project.org/package=magrittr) |
| **maps** | 3.3.0 | Original S code by Richard A. Becker, Allan R. Wilks. R version by Ray Brownrigg. Enhancements by Thomas P Minka and Alex Deckmyn. (2018). maps: Draw Geographical Maps. R package version 3.3.0. [https://CRAN.R-project.org/package=maps](https://cran.r-project.org/package=maps) |
| **crayon** | 1.4.1 | Gábor Csárdi (2021). crayon: Colored Terminal Output. R package version 1.4.1. [https://CRAN.R-project.org/package=crayon](https://cran.r-project.org/package=crayon) |
| **extrafontdb** | 1.0 | Winston Chang (2012). extrafontdb: Package for holding the database for the extrafont package. R package version 1.0. [https://CRAN.R-project.org/package=extrafontdb](https://cran.r-project.org/package=extrafontdb) |
| **car** | 3.0.11 | John Fox and Sanford Weisberg (2019). An [4] Companion to Applied Regression, Third Edition. Thousand Oaks CA: Sage. URL:<https://socialsciences.mcmaster.ca/jfox/Books/Companion/> |
| **pkgconfig** | 2.0.3 | Gábor Csárdi (2019). pkgconfig: Private Configuration for 'R' Packages. R package version 2.0.3. [https://CRAN.R-project.org/package=pkgconfig](https://cran.r-project.org/package=pkgconfig) |
| **ellipsis** | 0.3.2 | Hadley Wickham (2021). ellipsis: Tools for Working with .... R package version 0.3.2. [https://CRAN.R-project.org/package=ellipsis](https://cran.r-project.org/package=ellipsis) |
| **MASS** | 7.3.53.1 | Venables, W. N. & Ripley, B. D. (2002) Modern Applied Statistics with S. Fourth Edition. Springer, New York. ISBN 0-387-95457-0 |
| **data.table** | 1.14.0 | Matt Dowle and Arun Srinivasan (2021). data.table: Extension of `data.frame`. R package version 1.14.0. [https://CRAN.R-project.org/package=data.table](https://cran.r-project.org/package=data.table) |
| **assertthat** | 0.2.1 | Hadley Wickham (2019). assertthat: Easy Pre and Post Assertions. R package version 0.2.1. [https://CRAN.R-project.org/package=assertthat](https://cran.r-project.org/package=assertthat) |
| **R6** | 2.5.0 | Winston Chang (2020). R6: Encapsulated Classes with Reference Semantics. R package version 2.5.0. [https://CRAN.R-project.org/package=R6](https://cran.r-project.org/package=R6) |
| **compiler** | 4.0.5 | R Core Team (2021). R: A language and environment for statistical computing. R Foundation for Statistical Computing, Vienna, Austria. URL [https://www.R-project.org/](https://www.r-project.org/). |
| **dplyr** | 1.0.9 | Hadley Wickham, Romain François, Lionel Henry and Kirill Müller (2022). dplyr: A Grammar of Data Manipulation. R package version 1.0.9. [https://CRAN.R-project.org/package=dplyr](https://cran.r-project.org/package=dplyr) |
| **tidyr** | 1.2.0 | Hadley Wickham and Maximilian Girlich (2022). tidyr: Tidy Messy Data. R package version 1.2.0. [https://CRAN.R-project.org/package=tidyr](https://cran.r-project.org/package=tidyr) |
| **tibble** | 3.1.7 | Kirill Müller and Hadley Wickham (2022). tibble: Simple Data Frames. R package version 3.1.7. [https://CRAN.R-project.org/package=tibble](https://cran.r-project.org/package=tibble) |
| **ggpubr** | 0.4.0 | Alboukadel Kassambara (2020). ggpubr: 'ggplot2' Based Publication Ready Plots. R package version 0.4.0. [https://CRAN.R-project.org/package=ggpubr](https://cran.r-project.org/package=ggpubr) |
| **ggalt** | 0.4.0 | Bob Rudis, Ben Bolker and Jan Schulz (2017). ggalt: Extra Coordinate Systems, 'Geoms', Statistical Transformations, |
| **ggplot2** | 3.3.5 | H. Wickham. ggplot2: Elegant Graphics for Data Analysis. Springer-Verlag New York, 2016. |
| **ggridges** | 0.5.3 | Claus O. Wilke (2021). ggridges: Ridgeline Plots in 'ggplot2'. R package version 0.5.3. [https://CRAN.R-project.org/package=ggridges](https://cran.r-project.org/package=ggridges) |
| **stringr** | 1.4.0 | Hadley Wickham (2019). stringr: Simple, Consistent Wrappers for Common String Operations. R package version 1.4.0. [https://CRAN.R-project.org/package=stringr](https://cran.r-project.org/package=stringr) |
| **scales** | 1.2.0 | Hadley Wickham and Dana Seidel (2022). scales: Scale Functions for Visualization. R package version 1.2.0. [https://CRAN.R-project.org/package=scales](https://cran.r-project.org/package=scales) |
| **radarchart** | 0.3.1 | Doug Ashton and Shane Porter (2016). radarchart: Radar Chart from 'Chart.js'. R package version 0.3.1. [https://CRAN.R-project.org/package=radarchart](https://cran.r-project.org/package=radarchart) |

**References**

1. G. Batista, B.B., M. Monard, *Balancing Training Data for Automated Annotation of Keywords: a Case Study.* WOB, 2003.

2. Pedregosa, F., et al., *Scikit-learn: Machine Learning in Python.* Journal of Machine Learning Research, 2011. **12**: p. 2825-2830.

3. Lemaitre, G., F. Nogueira, and C.K. Aridas, *Imbalanced-learn: A Python Toolbox to Tackle the Curse of Imbalanced Datasets in Machine Learning.* Journal of Machine Learning Research, 2017. **18**.
