# Supplementary material for: MLcps: machine learning cumulative performance score for classification problems
Source: Gigascience. 2023 Dec 13;12:giad108. doi: 10.1093/gigascience/giad108 (PMC10716825; doi:10.1093/gigascience/giad108)
Supplement: giad108_Response_to_Reviewer_Comments_Original_Submission [file giad108_response_to_reviewer_comments_original_submission.pdf]

Author's Response To Reviewer Comments

######################
Reviewer #1
######################

<<---- Reviewer comment ---->>
Reviewer #1: Overall, I like the author's idea to combine metrics as another way to conduct model evaluation, in a way that takes both the magnitude and standard deviation of metrics scores into account. I think it's great that the authors have implemented this and shared it via gitub as an installable package. It's an interesting and potentially useful idea, but I believe this paper really needs to be expanded and improved before publication based on the comments laid out below.

! °°°° Response to Reviewer comment °°°°!
Thank you for taking the time to review our paper and for your valuable feedback. We appreciate your positive comments about MLcps. We understand your concerns about the need for further expansion and improvement before publication, and we are committed to addressing them to in the following text and manuscript to enhance the quality of our work. Your feedback is instrumental in helping us refine our manuscript. Below, you will find our response to the concerns you raised.

<<---- Reviewer comment ---->>
-This paper makes a lot of assumptions about how classification metrics are used to pick a best performing model. One being that minimizing standard deviation between metrics is always best, if this is the case, some citation or demonstration of this would be expected. In my experience, an model that maximizes some specific metric is often most desirable even at the expense of another metric not performing as well. This in itself is the foundation of using different decision thresholds in a model to (for example) maximize False Negatives for a given modeling task even at the expense of increased False Positives. Also while I agree that examinine multiple metrics is essential for picking a best model, creating a single meta-metric seems fundamentally risky and likely to lead to unforeseen issues in picking a best model under different use cases. This paper does not address this possibility.

! °°°° Response to Reviewer comment °°°°!
We appreciate your insightful feedback.
We'd like to clarify that we did not assert that minimizing standard deviation is the only optimal method for selecting the best-performing model. Rather the whole idea behind suggesting MLcps is to cautiously suggest another cumulative score that can compensate the shortcomings of SD. In this manuscript, we highlight that SD is one of the criteria commonly used in the field for such a purpose. We have also explicitly shown why it may not always be the most appropriate choice. We introduced the standard deviation criterion to emphasize how MLcps can identify the truly best performing model, even in situations, where relying solely on standard deviation criteria for model selection proves inadequate (please refer to lines 189-218).

We totally agree with your second comment, which underscores the standard practice of prioritizing specific metrics depending on the domain or stakeholders. Therefore, we have already integrated a weighting feature into MLcps, enabling users to specify which metric holds greater value for them. MLcps will then take this preference into account during the calculation process. For example, in some medical diagnosis tasks, sensitivity (true positive rate) might be more critical than specificity (true negative rate). Please refer to the lines 342-365.

Regarding the comment about the risks associated with combining metric scores, while there are valid concerns about creating a single meta-metric, the key is to use MLcps judiciously and in conjunction with domain knowledge. It's not a replacement for individual metrics or domain-specific considerations but rather a tool to aid in comprehensive model evaluation. When used wisely, it can provide a valuable additional perspective on model performance, especially in cases where multiple stakeholders with different metric preferences are involved.

We cannot envision a use case where MLcps inherently poses risks, as it combines precomputed metrics into a unified score. However, it's essential to understand that MLcps is only as good as the metrics used to calculate it. Recognizing which metrics are relevant to your models, a consideration influenced by factors such as domain, stakeholder preferences, and data characteristics, remains of paramount importance. Your comment has prompted us to emphasize this point to our readers and users. As a result, we have expanded the conclusion section to underscore the significance of selecting an appropriate evaluation metric based on specific domain or problem. Please refer to lines 425-436.

<<---- Reviewer comment ---->>
-Many of the figures are not using the space effectively to communicate the results. I'd suggest considering other ways to present/visualize these results more clearly. Figure 2 in particular is not a very effective way to clearly illustrate consistency in performance between training and testing sets on the individual datasets.

! °°°° Response to Reviewer comment °°°°!
We appreciate your input and have made significant changes based on your suggestions. We have revamped Figure 2 (previously) to improve clarity and space utilization. By combining the training and testing scores into a single plot, we have created a more concise representation of model performance. Additionally, we have incorporated model performance ranking information directly within the plot to enhance the comprehensibility of the results. We believe these modifications will better convey the consistency in performance between the training and testing sets across different datasets. Furthermore, we have updated the figure order. We have grouped all the plots related to CLL and Cervical datasets into figure 1, and the TCGA-related plots into figure 2. We believe these amendments will address your concerns effectively.

<<---- Reviewer comment ---->>
-The paper order of sections makes it difficult to understand the content presentation. I found it necessary to jump between sections to understand what the authors were doing and to try and understand their arguments.

! °°°° Response to Reviewer comment °°°°!
To address this issue, we have reorganized the order of the figures in the paper to enhance the logical flow of information. This adjustment should make it easier for readers to follow the sequence of our arguments and grasp the context. Additionally, we have incorporated introductory text into the results section. This introductory text is included to provide readers with a clear understanding of what to anticipate in the results section, thus facilitating a smoother transition between sections (lines 173-185).

We believe these changes will significantly enhance the readability and coherence of our paper, enabling readers to more effectively follow our arguments and gain a better understanding of the content.

<<---- Reviewer comment ---->>
-One of the points/observations of this paper is that low SD alone would not be a good meta metric, which seems like an obvious conclusion that could have been drawn without these experiments. I.e. a poor model with consistently low evaluation metric scores would have a low SD

! °°°° Response to Reviewer comment °°°°!
Thank you for your comment. We included this information to illustrate that MLcps, unlike SD criteria, can handle these kinds of situations and can help identify the truly best-performing model.

<<---- Reviewer comment ---->>
-The comparison of training and testing scores for the metric doesn't seem very useful given that training evaluations are largely not useful to begin with (as they really only indicate if an algorithm is learning anything or not from the data, whether it be signal or noise). Thus differences in metric values really only reflect potential overfitting which is a reflection on the algorithm rather than the metric.

! °°°° Response to Reviewer comment °°°°!

We agree about the limitations of relying solely on training results, and we appreciate your insight. In certain research domains, like the biological field with Proteomics/metabolomics/RNA-Seq data, obtaining a substantial amount of data can be particularly challenging due to sample scarcity. As a result, researchers often resort to using all available data for training and employ evaluation methods such as k-fold cross-validation to assess model performance.

In such situations, the luxury of having an independent test dataset to cross-check for overfitting may not be available. It's in these scenarios that we emphasize the importance of our findings. We showcased that MLcps tends to favor models that demonstrate robust generalization, not only excelling on the training data but also performing well on unseen datasets. This observation underscores the significance of using a comprehensive metric like MLcps, especially when researchers are compelled to rely on training data for their evaluations.

<<---- Reviewer comment ---->>
-Which metrics were included in MLcps, is not really addressed in the body of the paper and only appears to be laid out in Figure 3. It sounds like the authors are suggesting that the user can pick any number of initial metrics they want to include in MLcps, however it is not made explicitly clear which metrics were used in this papers's experiments (presumably the ones specified in Figure 3). This study would have benefitted from more specificity for reproducibility of these results, and further if the authors are suggesting that MLcps can be applied with whatever metrics a user wants to combine into the meta-metric, this paper should included evaluations examining different numbers and combinations of metrics into the MLcps calculation. How does the selection of different metrics and the diversity of what these metrics are good at capturing impact the MLcps scoring?

! °°°° Response to Reviewer comment °°°°!

Thank you for your comment. We would like to clarify that MLcps can be calculated using any evaluation metric. It is indeed a key aspect of MLcps that users have the flexibility to select metrics that align with their specific classification problem. However, it is highly recommended that all metrics are on the same scale (Normalised). For example, if accuracy ranges from 0 to 1, then the F1 metric should also be within the same range, rather than in percentage format. We have also included this information in the manuscript. Please refer to lines 271-274 for the details. Figure 3 is using a Schematic to describe the complete analysis process for MLcps Python package and in panel G we mentioned that for example F1, Jaccard score, Accuracy, ROC Accuracy, Average Precision, Precision, Recall can be used.

Regarding the exploration of "different numbers and combinations of metrics into the MLcps calculation," please refer to Figure S2, which showcases the use of a comprehensive set of seven distinct metrics, and Figure S3, where MLcps calculation is performed using five different metrics.

Figure S2: This figure presents the projection of metric scores onto a two-dimensional (2D) polar coordinate system for multiple machine learning algorithms trained on different example datasets, incorporating a total of seven metrics.

Figure S3: Similarly, Figure S3 provides a visual representation of metric scores projected onto a two-dimensional (2D) polar coordinate system, this time utilizing a set of five different metrics. These figures not only demonstrate the flexibility of MLcps but also highlight its adaptability to diverse metric combinations, ensuring robust performance evaluation across various datasets and scenarios.

<<---- Reviewer comment ---->>
-Furthermore, in Figure 3, it doesn't make any sense to include both accuracy and balanced accuracy into this cumulative score (as it is effectively weighting accuracy twice, since accuracy = balanced accuracy when the class balance is equal, and standard accuracy is a biased metric to begin with in contrast with balanced accuracy when the class balance is not equal.

! °°°° Response to Reviewer comment °°°°!

Thank you for your comment. Figure 3 is intended to illustrate the methodology of MLcps, and the metrics used are merely examples. However, to alleviate any potential confusion and address your concern, we have implemented your suggestion by replacing balanced accuracy with the Jaccard score in Figure 3.

<<---- Reviewer comment ---->>
-Metric weighting is discussed in this paper, but no evaluations or discussion of the risks of weighting is given.

! °°°° Response to Reviewer comment °°°°!
Thank you for your comment. We have extended the conclusion section to discuss the potential drawbacks associated with weighting. Please refer to lines 438-443 .

<<---- Reviewer comment ---->>
-Given the potential generalizability of this topic to machine learning problems in all sorts of application domains, it would be much more convincing to see analyses conducted on a much broader range of target problems/datasets (i.e. outside of the just the 4 RNA datasets presented).

! °°°° Response to Reviewer comment °°°°!

To demonstrate the generalizability of MLcps, we have included another dataset consisting of biometric information from 100,000 individuals, gathered from the National Health Insurance Service in Korea (line 385-397). Our objective was to predict alcohol consumption status using this extensive biometric dataset. We have observed a consistent trend in the results with this dataset as well (line 208,235-242).

<<---- Reviewer comment ---->>
-I believe there must be other related work dealing with combining evaluation metrics that the authors should cite and contrast their approach with. It would also be interesting for them to discuss metrics that evaluate a model using the same decision threshold vs. metrics that consider a range of decision thresholds (e.g. ROC AUC). Can this approach combine both? What would the implication of this be?

! °°°° Response to Reviewer comment °°°°!
Thank you for your valuable feedback.

We have conducted a thorough literature review and identified two studies as the most closely related work to our approach. In their studies, they employed a similar methodology to enhance Information Retrieval (IR) metrics by combining multiple evaluation measures (lines 155-159). It's worth noting that their work primarily outlined the methodology without providing a direct implementation, which limits our ability to make a direct comparison with MLcps. Nevertheless, apart from this study, we have not encountered any other research that introduces analogous methods for metric combination within classification domain.

Regarding your second comment, we appreciate your suggestion to incorporate both single-threshold and multi-threshold metrics MLcps calculation. While it is feasible to include both types of metrics in the calculation of MLcps, it is important to note that, as it stands, MLcps currently does not involve threshold criteria during its computation and does not provide control over thresholds. This limitation is primarily due to the nature of MLcps, which relies on precomputed metrics. Adjusting thresholds is typically a part of model training, and MLcps operates on the basis of these precomputed metrics. Although the inclusion of threshold control is an intriguing idea, it may fall outside the scope of our current manuscript, as MLcps is specifically designed to work with precomputed metrics and does not engage in threshold manipulation during its calculation. We appreciate your thought-provoking suggestion and will consider it for future research endeavors.

######################
Reviewer #2
######################

<<---- Reviewer comment ---->>
Reviewer #2: This paper presents machine learning cumulative performance score for classification problems. The authors proposed an evaluation metric used in classification. The authors integrated several pre-computed evaluation metrics in to an unified score. The authors tested their proposed system on four types of publicly available datasets. The authors used 15 reference papers considering the years 2003-2018. The authors should increse the number of references to be used for comparison purposes and the authors should consider recent year publications.

! °°°° Response to Reviewer comment °°°°!
We sincerely appreciate your time and effort in reviewing our paper, and we value your constructive feedback. In response to your suggestion, we have expanded our reference list to include recent studies that address various aspects of model evaluation, such as the use of multiple evaluation metrics and the impact of subject bias.

Regarding comparing MLcps, we have conducted a thorough literature review and identified two studies as the most closely related work to our approach. In their studies, they employed a similar methodology to enhance Information Retrieval (IR) metrics by combining multiple evaluation measures (lines 155-159). It's worth noting that their work primarily outlined the methodology without providing a direct implementation, which limits our ability to make a direct comparison with MLcps. Nevertheless, apart from this study, we have not encountered any other research that introduces analogous methods for metric combination within classification domain.
