# Supplementary material for: MLcps: machine learning cumulative performance score for classification problems
Source: Gigascience. 2023 Dec 13;12:giad108. doi: 10.1093/gigascience/giad108 (PMC10716825; doi:10.1093/gigascience/giad108)
Supplement: giad108_Response_to_Reviewer_Comments_Revision_1 [file giad108_response_to_reviewer_comments_revision_1.pdf]

Author's Response To Reviewer Comments

As per request we added a section titled "Data Availability" in addition to the "code availability" section. We have also included software application in the bio.tools and SciCrunch.org databases tand biotoolsID identifiers, and include these in your manuscript. We added the RRID and biotool IDs in the "code availability" section.
Thanks
