# Supplementary material for: MLcps: machine learning cumulative performance score for classification problems
Source: Gigascience. 2023 Dec 13;12:giad108. doi: 10.1093/gigascience/giad108 (PMC10716825; doi:10.1093/gigascience/giad108)
Supplement: giad108_Reviewer_1_Report_Original_Submission — Ryan J. Urbanowicz -- 7/24/2023 Reviewed [file giad108_reviewer_1_report_original_submission.pdf]

Reviewer Report

Title: MLcps: Machine Learning Cumulative Performance Score for Classification Problems

Version: Original Submission **Date:** 7/24/2023

Reviewer name: Ryan J. Urbanowicz

Reviewer Comments to Author:

Overall, I like the author's idea to combine metrics as another way to conduct model evaluation, in a way that takes both the magnitude and standard deviation of metrics scores into account. I think it's great that the authors have implemented this and shared it via gitub as an installable package. It's an interesting and potentially useful idea, but I believe this paper really needs to be expanded and improved before publication based on the comments laid out below.-This paper makes a lot of assumptions about how classification metrics are used to pick a best performing model. One being that minimizing standard deviation between metrics is always best, if this is the case, some citation or demonstration of this would be expected. In my experience, an model that maximizes some specific metric is often most desirable even at the expense of another metric not performing as well. This in itself is the foundation of using different decision thresholds in a model to (for example) maximize False Negatives for a given modeling task even at the expense of increased False Positives. Also while I agree that examinine multiple metrics is essential for picking a best model, creating a single meta-metric seems fundamentally risky and likely to lead to unforeseen issues in picking a best model under different use cases. This paper does not address this possibility.-The paper order of sections makes it difficult to understand the content presentation. I found it necessary to jump between sections to understand what the authors were doing and to try and understand their arguments.-Many of the figures are not using the space effectively to communicate the results. I'd suggest considering other ways to present/visualize these results more clearly. Figure 2 in particular is not a very effective way to clearly illustrate consistency in performance between training and testing sets on the individual datasets.-One of the points/observations of this paper is that low SD alone would not be a good meta metric, which seems like an obvious conclusion that could have been drawn without these experiments. I.e. a poor model with consistently low evaluation metric scores would have a low SD-The comparison of training and testing scores for the metric doesn't seem very useful given that training evaluations are largely not useful to begin with (as they really only indicate if an algorithm is learning anything or not from the data, whether it be signal or noise). Thus differences in metric values really only reflect potential overfitting which is a reflection on the algorithm rather than the metric.-Which metrics were included in MLcps, is not really addressed in the body of the paper and only appears to be laid out in Figure 3. It sounds like the authors are suggesting that the user can pick any number of initial metrics they want to include in MLcps, however it is not made explicitly clear which metrics were used in this papers's experiments (presumably the ones specified in Figure 3). This study would have benefitted from more specificity for reproducibility of these results, and further if the authors are suggesting that MLcps can be applied with whatever metrics a user wants to combine into the meta-metric, this paper should included evaluations examining different numbers and combinations of metrics into the MLcps calculation. How does the

selection of different metrics and the diversity of what these metrics are good at capturing impact the MLcps scoring?-Furthermore, in Figure 3, it doesn't make any sense to include both accuracy and balanced accuracy into this cumulative score (as it is effectively weighting accuracy twice, since accuracy = balanced accuracy when the class balance is equal, and standard accuracy is a biased metric to begin with in contrast with balanced accuracy when the class balance is not equal.-Metric weighting is discussed in this paper, but no evaluations or discussion of the risks of weighting is given.-Given the potential generalizability of this topic to machine learning problems in all sorts of application domains, it would be much more convincing to see analyses conducted on a much broader range of target problems/datasets (i.e. outside of the just the 4 RNA datasets presented).-I believe there must be other related work dealing with combining evaluation metrics that the authors should cite and contrast their approach with. It would also be interesting for them to discuss metrics that evaluate a model using the same decision threshold vs. metrics that consider a range of decision thresholds (e.g. ROC AUC). Can this approach combine both? What would the implication of this be?

Level of Interest

Please indicate how interesting you found the manuscript: Choose an item.

Quality of Written English

Please indicate the quality of language in the manuscript: Choose an item.

Declaration of Competing Interests

Please complete a declaration of competing interests, considering the following questions:

- Have you in the past five years received reimbursements, fees, funding, or salary from an organisation that may in any way gain or lose financially from the publication of this manuscript, either now or in the future?
- Do you hold any stocks or shares in an organisation that may in any way gain or lose financially from the publication of this manuscript, either now or in the future?
- Do you hold or are you currently applying for any patents relating to the content of the manuscript?
- Have you received reimbursements, fees, funding, or salary from an organization that holds or has applied for patents relating to the content of the manuscript?
- Do you have any other financial competing interests?
- Do you have any non-financial competing interests in relation to this paper?

If you can answer no to all of the above, write 'I declare that I have no competing interests' below. If your reply is yes to any, please give details below.

I declare that I have no competing interrests.

I agree to the open peer review policy of the journal. I understand that my name will be included on my report to the authors and, if the manuscript is accepted for publication, my named report including any attachments I upload will be posted on the website along with the authors' responses. I agree for my report to be made available under an Open Access Creative Commons CC-BY license (http://creativecommons.org/licenses/by/4.0/). I understand that any comments which I do not wish to be included in my named report can be included as confidential comments to the editors, which will not be published.

Choose an item.

To further support our reviewers, we have joined with Publons, where you can gain additional credit to further highlight your hard work (see: https://publons.com/journal/530/gigascience). On publication of this paper, your review will be automatically added to Publons, you can then choose whether or not to claim your Publons credit. I understand this statement.

Yes Choose an item.
